# Genome-wide characterization and expression profiling of *Eucalyptus grandis* HD-Zip gene family in response to salt and temperature stress

**DOI:** 10.1186/s12870-020-02677-w

**Published:** 2020-10-01

**Authors:** Jiashuo Zhang, Jinzhang Wu, Mingliang Guo, Mohammad Aslam, Qi Wang, Huayan Ma, Shubin Li, Xingtan Zhang, Shijiang Cao

**Affiliations:** 1 College of Forestry, Fujian Agriculture and Forestry University, Fuzhou, 350002 Fujian China; 2 College of Plant Protection, Fujian Agriculture and Forestry University, Fuzhou, 350002 Fujian China; 3 Center for Genomics and Biotechnology, Fujian Provincial Key Laboratory of Haixia Applied Plant Systems Biology, Key Laboratory of Genetics, Breeding and Multiple Utilization of Corps, Ministry of Education, Fujian Agriculture and Forestry University, Fuzhou, 350002 Fujian China; 4 State Key Laboratory for Conservation and Utilization of Subtropical Agro Bioresources, Guangxi Key Lab of Sugarcane Biology, College of Agriculture, Guangxi University, Nanning, 530004 Guangxi China

**Keywords:** HD-zip, *Eucalyptus grandis*, Transcription factors, Bioinformatic analysis

## Abstract

**Background:**

The HD-Zip transcription factors are unique to plants and play an essential role in plant growth, development and stress responses. The HD-Zip transcription factor family consists of a highly conserved homeodomain (HD) and a leucine zipper domain (LZ) domain. Although the HD-Zip gene family has been extensively studied in many plant species, a systematic study of the *Eucalyptus* HD-Zip family has not been reported until today. Here, we systematically identified 40 HD-Zip genes in *Eucalyptus* (*Eucalyptus grandis*). Besides, we comprehensively analyzed the HD-Zips of *Eucalyptus* by studying the homology, conserved protein regions, gene structure, 3D structure of the protein, location of the genes on the chromosomes and the expression level of the genes in different tissues.

**Results:**

The HD-Zip family in *Eucalyptus* has four subfamilies, which is consistent with other plants such as *Arabidopsis* and rice. Moreover, genes that are in the same group tend to have similar exon-intron structures, motifs, and protein structures. Under salt stress and temperature stress, the *Eucalyptus* HD-Zip transcription factors show a differential expression pattern.

**Conclusions:**

Our findings reveal the response of HD-Zip transcription factors under salt and temperature stresses, laying a foundation for future analysis of *Eucalyptus* HD-Zip transcription factors.

## Background

Transcription factors (TFs) are essential proteins that bind to a specific *cis*-acting element of a gene’s promoter region to activate or inhibit its transcription, thus play crucial functions in the signaling pathway. Transcription factors are also an essential participant in the processes of eukaryotic growth and differentiation [1, 2]. By forming complex networks, TFs can regulate the expression of various genes in both dimensions of time and space. Therefore, they possess the potential to become a useful tool for improving traits of economic and ecological importance [3, 4]. Till today, several TFs genes have been cloned that participate in abiotic stress responses such as AP2/EREBP, NAC, WRKY, MYB, HSF, ZFP and bHLH [5, 6].

The homologous domain leucine zipper (HD-Zip) gene family is reported only in the plants and regulates plant-specific growth and development processes [7, 8]. HD-Zip possesses a highly conserved homeodomain (HD) composed of 61 amino acids and a Leucine zipper (LZ) domain. The HD motif can specifically bind to DNA, whereas LZ serves as a dimerization motif [9]. HD-Zips have been identified and analyzed in various plant species, such as *Medicago truncatula*, grape (*Vitis vinifera*), rice (*Oryza sativa*), maize (*Zea mays*), potato (*Solanum tuberosum*), wheat (*Triticum aestivum*) and banana [10–17]. The HD-Zips are divided into four subfamilies: HD-Zip I, HD-Zip II, HD- Zip III, HD-Zip IV based on their gene structure, conserved sequences, *cis*-elements and biological functions [18, 19]. Similarly, *Eucalyptus* also has four subfamilies, HD-Zip I to HD-Zip IV. Among them, the HD-Zip I contains both of the two basic motifs; however, the HD-Zip II, III and IV families possess other motifs in addition to HD and LZ motifs. 1) CPSCE (consisting of five conserved amino acid sequences of Cys, Pro, Ser, Cys, and Glu) exist in the HD-Zip II subfamily [20, 21]. 2) In the HD-Zip III and IV subfamilies, a steroidogenic acute regulatory protein motif START (star-related lipid transfer), which is associated with lipid transfer, is found [22, 23]. HD-Zip I and HD-Zip II proteins recognize a similar pseudopalindromic sequence CAAT(C/G)ATTG [7, 20, 21], while HD-Zip III and HD-Zip IV proteins recognize the sequences GTAAT(G/C)ATTAC and TAAATG(C/T)A, respectively [22, 24].

HD-Zip I proteins are reported for their function in plant light signal transduction [5], leaf and seed development [20, 24], plant growth, de-yellowing and plant response to stress [1, 21, 25]. In *Arabidopsis*, when overexpressed, an HD-Zip I member ATHB12, results in big leaves and enlarged cells, displaying its role in leaf growth. While another HD-Zip I member, ATHB1 contributes to cell wall composition and elongation [18, 26]. Besides, Federico et al. proved that *Medicago truncatula* HD-Zip I TF HB1 is required for lateral root development [16, 18]. Moreover, HD-Zip I proteins also play a vital role during abiotic stress responses. For example, drought and abscisic acid strongly upregulate ATHB7 and ATHB12, that act as positive regulators of PP2C in *Arabidopsis* [27]. In sunflower, HaHB4 regulates tolerance against drought conditions through ethylene-mediated aging [28]. Besides, overexpression of ZmHDZip10 and TaHDZipI-5 can improve plant tolerance to low temperature, drought, or salinity stress [29–31]. HD-Zip I family members also have a defensive role in combating biotic stresses. For example, the pepper HD-Zip I protein has a positive effect on increased tolerance against *Ralstonia solanacearum* [32].

HD-Zip II subfamily possesses a conserved domain CPSCE, which plays an essential role in mediating plant response to changes in light quality and shading [33, 34], and abiotic stress responses [35]. Photochemical conditions mainly regulate their expression [36, 37].

The structure of the HD-Zip III proteins is most complex among HD-Zips. The HD-Zip III family possesses the START domain, homeodomain-START associated domain (HD-SAD) and Met-Glu-Lys-Hi-Leu-Ala (MEKHLA) domain. The START domain is ABA-responsive, and the function of the HD-SAD domain is currently unknown. It is worth noting that the HD domain of HD-Zip III has two altered amino acid residues compared to the other subfamilies, and this change may be related to the unique MEKHLA domain [38]. The MEKHLA domain is a PAS-like domain associated with several chemical and physical stimuli [39]. HD-Zip III proteins not only participate in plant-specific photosynthetic processes but also inhibit the transcription [40], implying that HD-Zip III genes might play a role in transcription [40]. HD-Zip III proteins are also believed to be involved in plant embryo development [41], meristem formation [39], vascular development [42], and polar transport of auxin during plant development [10].

microRNAs (miRNAs) are small non-coding RNAs that post-transcriptionally regulate the gene expression [43]. miRNAs bind to complementary mRNA molecules and negatively regulate the expression of targets through slicing or translational repression [44]. The class III HD–Zip genes are reported to be post-transcriptionally regulated by the microRNAs miR165/166 [45, 46]. In *Arabidopsis* developing roots, cross-talk of at least six different phytohormones dynamically regulates the spatio-temporal expression pattern of miR165/166 and HD-Zip III. Besides, HD-Zip III mediated root development is modulated transcriptionally through phytohormones and KANs, and post-transcriptionally by miR165/166 [47]. Class II and class III HD-Zips determine the correct patterning of upper and lower leaf tissues, and together repress miRNAs miR165/166, that ultimately regulates class III HD-Zips function. This three-way interaction maintains tissue identity balance during development, which helps in the development of a flat leaf in *Arabidopsis* [48, 49]. Also, miR166g over-expression in *Arabidopsis jabba*-1D (*jba*-1D) mutant plant affects the transcripts of class III homeodomain-leucine zipper family genes [50].

The HD-Zip IV subfamily contains four conserved domains identical to the HD-Zip III subfamily, the HD, Zip, START and HD-SAD domains. However, HD-Zip IV lacks the MEKHLA domain [51, 52]. The genes of this subfamily are expressed explicitly in the outer cell, epidermal, sub-epidermal cells of multiple species during biotic and abiotic stresses [53, 54]. HD-Zip IV also plays a vital role in trichome formation, anthocyanin accumulation, lipid biosynthesis and transport [53–56].

*Eucalyptus,* a native plant of the southeastern coast of Australia, is a member of the genus *Myrtaceae*. In China, *Eucalyptus* is mainly planted in Guangxi province. It enjoys moist and fertile river loam soil and red soil weathered by basalt. *Eucalyptus* has high economic value due to its short growth cycle and other advantages. The leaves of *Eucalyptus* can be used to extract aromatic oils [57]. Besides, it can also be used as medicine due to its anti-inflammatory, sterilizing and expectorant effects [58]. *Eucalyptus* wood has corrosion resistance characteristics, and it is widely used in such construction, papermaking and fuel industries. Also, it is commonly used in artificial afforestation around the world, and as a unique commercial tree species, *Eucalyptus* is planted on a large scale. However, *Eucalyptus* is often affected by abiotic stresses such as drought, low temperature, and salt stress during its growth, resulting in a decline in the yield [59–61]. Therefore, it is crucial to study and analyze the stress response-related genes of *Eucalyptus* to improve the excellent traits and breeding.

Here, we identified and classified the HD-Zip proteins of *Eucalyptus*, analyzed the relationship between the HD-Zip family and HD-Zips responses to temperature and salinity stress. These results provide the necessary basis for the functional characterization of the *Eucalyptus* HD-Zip family and a theoretical basis for the subsequent improvement of *Eucalyptus* varieties against stress.

## Results

### Phylogenetic and evolutionary analysis of HD-Zip proteins in *Eucalyptus*

**Fig. 1:**
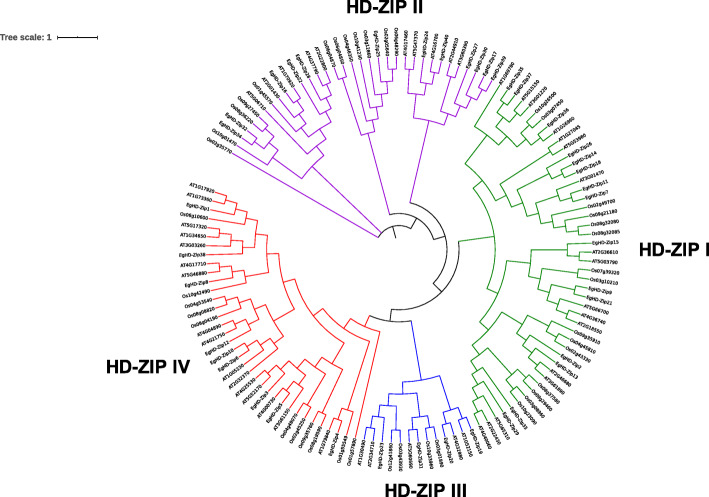
Phylogenetic classification of HD-ZIP gene in *Eucalyptus grandis*, *Arabidopsis thaliana* and *Oryza sativa*. The phylogenetic tree was constructed using iQTREE software. The topological structure of the ML tree shows that the HD-Zip genes of *Eucalyptus*, Arabidopsis and rice can be divided into four subfamilies, namely EgHD-Zip I, EgHD-Zip II, EgHD-Zip III, EgHD-Zip IV. In the figure, yellow represents the HD-Zip I subfamily of *Arabidopsis*, rice and *Eucalyptus*, blue represents the HD-Zip II subfamily of *Arabidopsis*, rice and *Eucalyptus*, and green represents the HD-Zip III subtype of *Arabidopsis*, rice and *Eucalyptus*. The orange represents the HD-ZipI IV subfamily of *Arabidopsis*, rice and *Eucalyptus*

A total of 40 candidate HD-Zip genes were identified in *Eucalyptus* and named as EgHD-Zip 1–40. To further analyze the selected sequences, a phylogenetic tree consisting of the 40 sequences was constructed. Also, to better highlight the difference between *Eucalyptus* and other plants, *Arabidopsis* and rice were included in the evolutionary analysis. The results showed that *Eucalyptus*, like *Arabidopsis* and rice, also had 4 subfamilies of HD-Zip genes. Among them, the HD-Zip I subfamily has the maximum members (15 Nos), and the HD-Zip III subfamily has the least members (4 Nos) (Fig. 1, Supplementary Fig. S1, S2).

In the II subfamily, EgHD-Zip16 and AT1G70920 (ATHB18), EgHD-Zip40 and AT4G16780 (ATHB2) showed a high homology. Besides, in the subfamily I, EgHD-Zip36 had high homology with AT1G26960 (ATHB23). In the subfamily III, EgHD-Zip19 and AT1G52150 (ATHB15) were highly homologous, so it can be speculated that EgHD-Zip19 may also have a similar role in inducing the development of vascular bundles. Similarly, EgHD-Zip20 and AT4G32880 (ATHB8) were on the same branch, so it can be speculated that EgHD-Zip20 could also induce the development of vascular bundles. In subfamily IV, EgHD-Zip4 had high homology with AT1G79840 (ATHB10), EgHD-Zip5 and AT4G00730 (ANL2), suggesting that EgHD-Zip4 may be involved in the epidermal cell differentiation process, and EgHD-Zip5 can affect flower development. The accumulation of glucoside in the leaf epidermis, and when the mutation occurs, it will inhibit the accumulation of anthocyanin. At the same time, it may also be involved in determining the identity of cells in the root [7].

As shown in Table 1, the isoelectric points of proteins from the second subfamily are more than 7, suggesting these proteins contain more basic amino acids. While the isoelectric point for the genes from other subfamilies, is generally less than 7, suggesting each protein contains more acidic amino acids. Besides, EgHD-Zip proteins are 91 to 848 amino acids long, with an average length of 430 amino acids, a molecular weight of 10.39 kDa to 93.01 kDa, and an average of 47.51 kDa. Subcellular localization analysis indicates that the *Eucalyptus* HD-Zip genes are all localized in the nucleus.

**Table 1:** List of identified HD-Zip genes in *Eucalyptus* and their properties

ID	Gene name	Subfamily	Length	PI	MW	Localization
Eucgr.H00609.1.p	EgHD-Zip1	IV	712	6.16	78,575.15	Nucleus
Eucgr.A01421.1.p	EgHD-Zip2	I	218	5.12	25,294.18	Nucleus
Eucgr.K00234.1.p	EgHD-Zip3	IV	757	6.03	83,309.37	Nucleus
Eucgr.F03040.1.p	EgHD-Zip4	IV	758	6.03	83,837.86	Nucleus
Eucgr.D02248.1.p	EgHD-Zip5	IV	840	5.9	89,950.16	Nucleus
Eucgr.A01715.1.p	EgHD-Zip6	IV	772	5.58	83,947.74	Nucleus
Eucgr.I01817.1.p	EgHD-Zip7	I	267	4.6	30,744.19	Nucleus
Eucgr.D02223.1.p	EgHD-Zip8	IV	793	5.55	87,360.15	Nucleus
Eucgr.H01254.1.p	EgHD-Zip9	I	184	5.9	20,705.33	Nucleus
Eucgr.K00800.1.p	EgHD-Zip10	IV	726	5.62	79,852.39	Nucleus
Eucgr.J02186.1.p	EgHD-Zip11	I	244	4.88	27,526.4	Nucleus
Eucgr.E04108.1.p	EgHD-Zip12	IV	738	5.67	81,384.11	Nucleus
Eucgr.D02645.1.p	EgHD-Zip13	I	234	4.97	26,926.97	Nucleus
Eucgr.B00423.1.p	EgHD-Zip14	I	315	4.8	35,758.36	Nucleus
Eucgr.A01575.1.p	EgHD-Zip15	I	242	7.2	27,489.85	Nucleus
Eucgr.G02186.1.p	EgHD-Zip16	II	228	8.98	25,157.36	Nucleus
Eucgr.E02798.1.p	EgHD-Zip17	II	245	8.68	27,127.48	Nucleus
Eucgr.K02448.1.p	EgHD-Zip18	I	308	4.83	34,624.15	Nucleus
Eucgr.F03066.1.p	EgHD-Zip19	III	844	6.07	92,950.33	Nucleus
Eucgr.C00605.1.p	EgHD-Zip20	III	836	5.81	91,942.23	Nucleus
Eucgr.I02464.1.p	EgHD-Zip21	I	214	6.56	24,942.18	Nucleus
Eucgr.I00660.1.p	EgHD-Zip22	II	264	8.87	29,626.54	Nucleus
Eucgr.D00184.1.p	EgHD-Zip23	III	848	6.09	93,008.23	Nucleus
Eucgr.D02105.1.p	EgHD-Zip24	II	287	7.01	31,824.69	Nucleus
Eucgr.C00074.1.p	EgHD-Zip25	II	234	7.54	26,098.06	Nucleus
Eucgr.B03409.1.p	EgHD-Zip26	I	317	4.64	35,629.04	Nucleus
Eucgr.E01577.1.p	EgHD-Zip27	II	297	7.03	32,686.62	Nucleus
Eucgr.F02206.1.p	EgHD-Zip28	II	252	8.64	27,926.63	Nucleus
Eucgr.H04742.1.p	EgHD-Zip29	I	343	5.47	38,171.11	Nucleus
Eucgr.L00494.1.p	EgHD-Zip30	II	91	7.78	10,392.55	Nucleus
Eucgr.B02504.1.p	EgHD-Zip31	III	843	5.66	92,361.44	Nucleus
Eucgr.A02462.1.p	EgHD-Zip32	II	326	8.37	35,949.34	Nucleus
Eucgr.F00378.2.p	EgHD-Zip33	I	277	5.76	31,099.67	Nucleus
Eucgr.K01847.1.p	EgHD-Zip34	II	365	8.42	40,259.87	Nucleus
Eucgr.I01934.1.p	EgHD-Zip35	I	239	7.92	27,035.21	Nucleus
Eucgr.G02520.1.p	EgHD-Zip36	I	293	5.9	33,219.03	Nucleus
Eucgr.I02255.1.p	EgHD-Zip37	I	188	9.38	21,505.07	Nucleus
Eucgr.F02903.1.p	EgHD-Zip38	IV	719	7.62	79,902.89	Nucleus
Eucgr.H02817.1.p	EgHD-Zip39	II	237	8.17	26,483.64	Nucleus
Eucgr.E00400.1.p	EgHD-Zip40	II	318	8.46	35,158.26	Nucleus

### Analysis of EgHD-Zip gene structure and motif structure

**Fig. 2:**
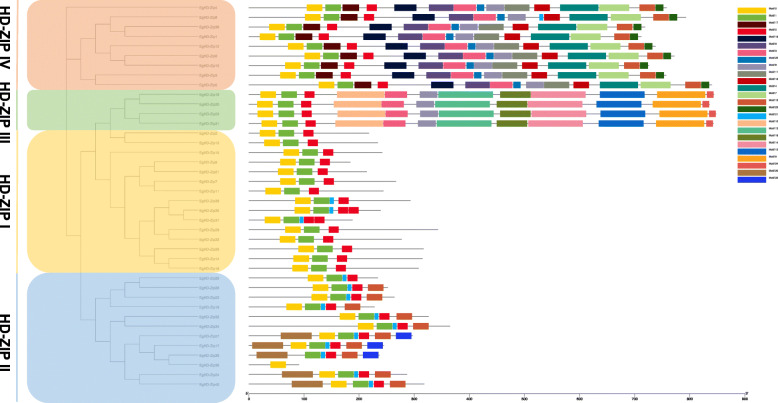
Phylogenetic relationship and sequence composition of HD-Zip family in *Eucalyptus*. In the figure, yellow represents the HD-Zip I subfamily of *Eucalyptus*, blue represents the HD-Zip II subfamily of *Eucalyptus*, and green represents the HD-Zip III subfamily of *Eucalyptus*. The orange represents the HD-ZipI IV subfamily of *Eucalyptus*

Multiple Em for Motif Elicitation (MEME) and Gene Structure Display Server (GSDS) were used to understand the relationship between EgHD-Zip proteins and their structure. It is worth noting that all of the EgHD-Zip proteins possessed the conserved motif encoding the HD and LZ domains. Highly homologous members were composed of the same motif, indicating that HD-Zip proteins of the same subfamily could have similar functions. The results also suggest that all EgHD-Zip proteins except EgHD-Zip30 have motifs 1, 2 and 5. Motifs 1 and 2 represent the conserved motifs encoding the HD domain, and motif 5 represents the encoding LZ conserved motifs of the domain (Fig. 2). The motif 12 encoding the CPSCE domain is found in all members of the HD-Zip II subfamily. However, HD-Zip-N is less conservative in the HD-Zip II subfamily because it was not found in EgHD-Zip16, − 22, − 25, − 28, − 30, − 32, and − 34. Also, the motif of the START domain was found in subfamilies III and IV. The MEKHLA domain encoded by motif 8 and motif 24 is unique to the subfamily III and found only in 4 members (Supplementary Fig. S3, S4).

**Fig. 3:**
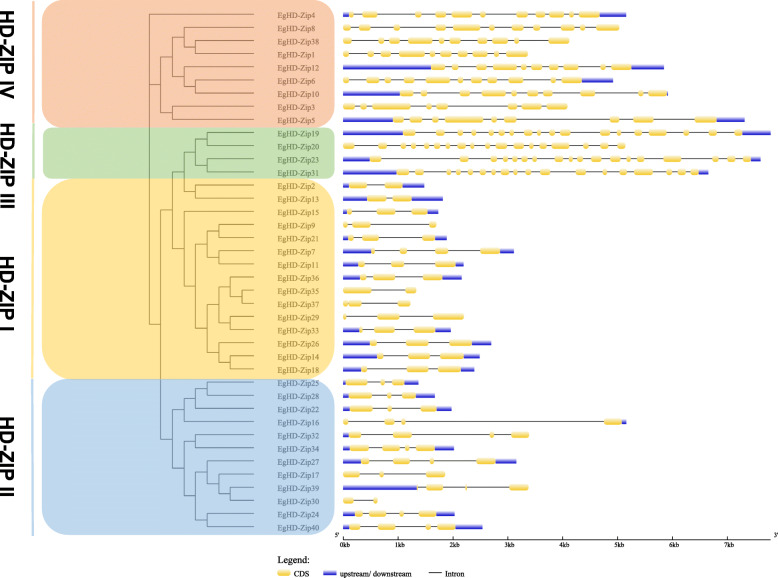
Genetic structure of *Eucalyptus* HD-Zip. Exon-intron gene structures of EgHD-Zip. Gene structure analysis was performed using GSDS. The length of exons and introns for each EgHD-Zip gene are proportionally displayed

The Gene Structure Display Server (GSDS) was used to analyze the structural diversity of the HD-Zip genes in *Eucalyptus* (Fig. 3). The results showed that the genetic structure of the 40 HD-Zip genes differed significantly in exon/intron arrangement and the number of introns. At the same time, the most relevant members in the same subfamily had similar exons/introns. The intron structure and the number of introns were consistent with the characteristics defined in the phylogenetic analysis described above. Also, the structure of the subfamily III genes was most sophisticated among the HD-Zip gene family, consistent with the motif analysis results. For example, the subfamily I and subfamily II HD-Zip genes contain 2 to 4 exons, the subfamily IV HD-Zip genes contain 8 to 11 exons, and the subfamily III HD-Zip genes contain 18 exons.

**Fig. 4:**
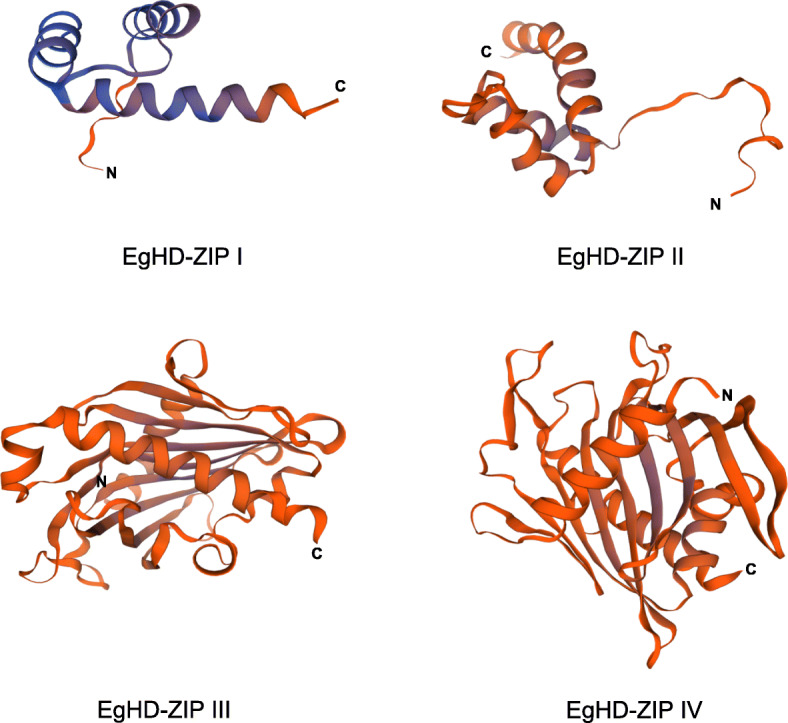
Homology modeling of HD-Zip protein in *Eucalyptus*. In the figure, the 3D structures of the four subfamily proteins were selected from the representative genes of each family. Among them, EgHD-Zip I is the structure of EgHD-Zip 33, EgHD-Zip II is the structure of EgHD-Zip 40, EgHD-Zip III is EgHD-Zip 23, EgHD-Zip IV is the structure of EgHD-Zip 38

To obtain intron gain/loss information for all sister pairs, we also compared the intron/exon structure of genes clustered at the end branches of the phylogenetic tree. Among them, 5 pairs showed intron/exon structural changes, including HD-Zip6/− 10, HD-Zip3/− 5, HD-Zip7/− 11, HD-Zip35/− 37, and HD-Zip39/− 30 (Fig. 4), these changes only occurred in the subfamilies I, II, and IV. By comparing with neighboring genes, we found that HD-Zip7 and HD-Zip6 have obtained an intron during the evolutionary process, while HD-Zip35 has lost an intron (Fig. 4).

### 3D structure analysis of EgHD-Zip

Using the protein homology modeling method based on the *Eucalyptus* HD-Zip structure of the Swissmodel database, the structures of the members from four subfamilies of *Eucalyptus* HD-Zip were predicted. The N- and C-termini of the 3D structure of EgHD-Zip protein are shown in Fig. 4. The structure with the highest score was chosen as the optimal structure for the EgHD-Zip protein. The 3D structure of each subfamily has a significant difference, and the 3D structure of HD-Zip III and HD-Zip IV was most complicated. Consistent with previous studies, HD-Zip I has some conserved structures at the carboxyl-terminal region (CTR) and amino-terminal region (NTR) [62]. As the simulation analysis revealed, the four subfamilies have significant differences in genes at the protein structure level.

### Chromosome localization of EgHD-Zip genes

**Fig. 5:**
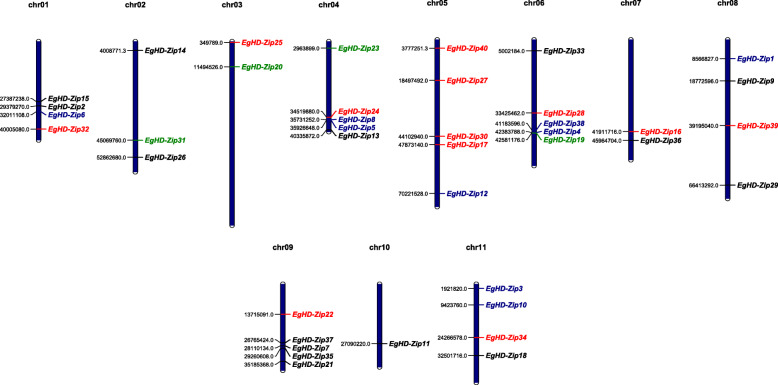
Chromosomal localization of EgHD-Zip genes on the 11 *Eucalyptus* chromosomes. The number of chromosomes is displayed at the top of each vertical bar. The position data is displayed on the right side of the chromosome, and a short line connects the corresponding EgHD-Zip gene names on the left side, and different colors represent different subfamilies. Black is the EgHD-Zip I subfamily, red is the EgHD-Zip II subfamily, green is the EgHD-Zip III subfamily, blue is the EgHD-Zip IV subfamily. Chr, chromosome

MapChart software was used to determine the distribution of EgHD-Zip gene based on their position on 11 chromosomes in *Eucalyptus*. We found that the distribution of HD-Zip genes in *Eucalyptus* was uneven. Chromosome 10 had only one HD-Zip gene, while the remaining chromosomes had two to five genes (Fig. 5). There were four genes of the first subfamily on chromosome 9, and subfamily II was mainly distributed on chromosome 5. The genes of subfamily III and IV were scattered in the *Eucalyptus* chromosome, and subfamily IV was on number 1. There were 1 or 2 genes on chromosomes 4, 5, 6, and 8 (Fig. 5).

### Expression profiles of *Eucalyptus* HD-Zip genes in various tissues

**Fig. 6:**
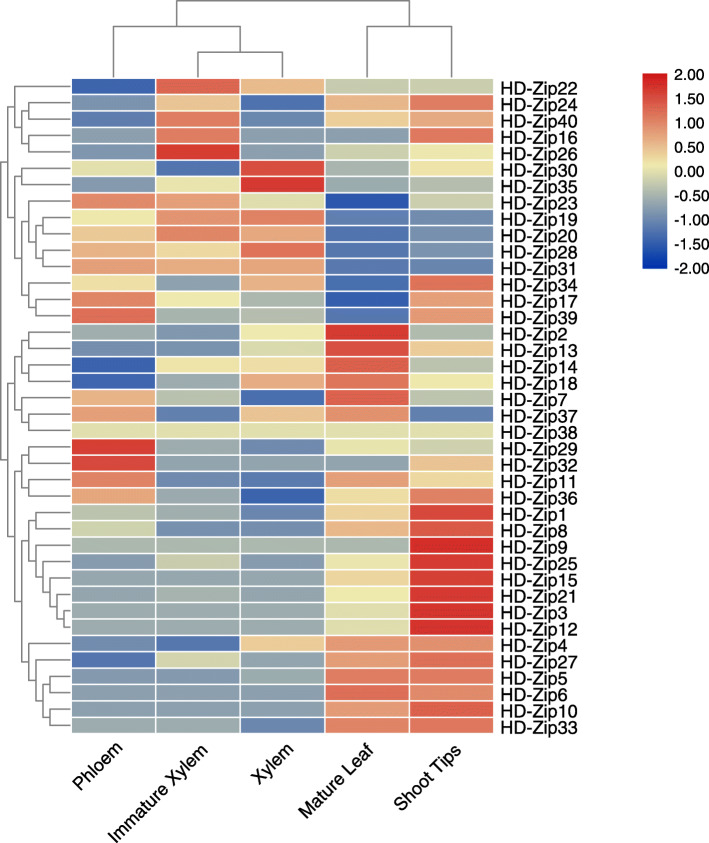
Relative expression levels of EgHD-Zip genes in various tissues. Expression profiles of EgHD-Zip genes in young leaf, xylem, shoot tips, phloem, mature leaf and immature xylem of *Eucalyptus*. Red indicates that the gene is highly expressed in this tissue, and blue indicates low expression. The color scale is shown on the right side of the graph. The prefix “Eg” stands for *Eucalyptus grandis*

For this experiment, samples from mature leaves, phloem, young xylem, mature xylem, and shoot tip of *Eucalyptus* were selected, and an expression heat map of EgHD-Zip genes was generated using TBtools (Fig. 6, Supplementary Fig. S5, S6, S7, S8). The analysis showed that in subfamily IV, except for EgHD-Zip4 and EgHD-Zip38, the expression levels of other genes in xylem and phloem were lower. In comparison, the expression level in stem tip and leaves were higher, indicating that this family may play a role in the growth and development of apical meristems and leaves. Also, the four genes of subfamily III were highly expressed in xylem and phloem, suggesting that the genes from subfamily III could be involved in the development of vascular bundles, which may be related to the transport of plant hormones. In subfamily I, the expression levels of 8 genes EgHD-Zip2, − 7, − 11, − 13, − 14, − 18, − 33 and − 37 were higher in mature leaves. The expression levels of EgHD-Zip9, − 15, − 21, − 33, and − 36 were higher in the shoot tip, which may be related to the growth and development of leaves and the development of apical meristems. It is worth noting that three genes were highly expressed in vascular tissue. Among them, EgHD-Zip26 was highly expressed in young xylem tissues and may play a unique role in the formation and development of xylem. Besides, EgHD-Zip35 was highly expressed in mature xylem, and it could be related to the transport of hormones, water and nutrients in plants. Similarly, EgHD-Zip29 was also highly expressed in the phloem, suggesting that it may play an important role in the development of the phloem. In subfamily II, 67% of genes are related to the growth and development of vascular bundles and the transport of plant hormones. Interestingly, some genes had low or no expression in the selected samples, which does not mean that these genes are not essential. Instead, it may be expressed in other developmental stages. In general, we found each subfamily has its unique expression characteristics and thus plays a different role.

### EgHD-Zip gene expression profiles in response to salt

**Fig. 7:**
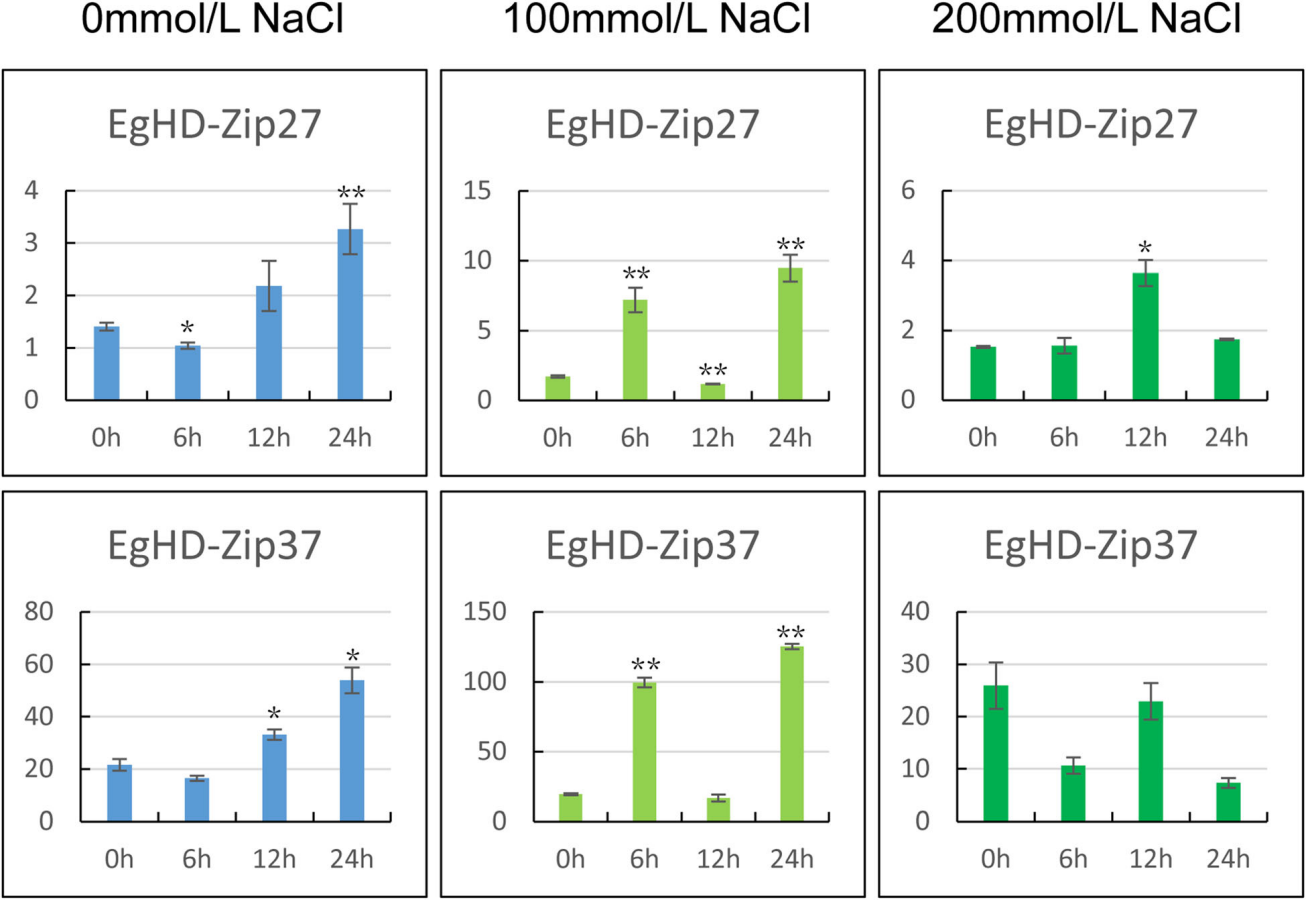
RT-PCR expression profiles of five subfamilies of genes in HD-Zip in response to salt stress. The RNA was isolated from the young leaves of the treated *Eucalyptus* annual seedlings. The low salinity was treated with 100 mmol/L NaCl, the high salinity was treated with 200 mmol/L NaCl, and the control group was treated with distilled water. The processing time of each group was 0 h, 6 h, 12 h and 24 h

The response of *Eucalyptus* to temperature and salt stress is more prominent. We found that the response trend of EgHD-Zip27 and EgHD-Zip37 was the same under 0 mmol/L NaCl treatment. At 0 mmol/L NaCl EgHD-Zip27 and EgHD-Zip37 showed a decrease after 6 h followed by an increase suggesting that the plant’s biological clock could regulate the expression levels of EgHD-Zip27 and EgHD-Zip37. At 100 mmol/L NaCl treatment, the expression levels of EgHD-Zip27 and EgHD-Zip37 were much higher compared to 0 mmol/L NaCl treatment, and first showed an increasing trend, then a decrease followed by an increase. Though the expression levels of EgHD-Zip27 and EgHD-Zip37 were significantly decreased at 200 mmol/L NaCl treatment, yet it was higher compared to 0 mmol/L NaCl treatment. Taken together, it can be inferred that EgHD-Zip27 and EgHD-Zip37 have essential roles in coping with NaCl stress (Fig. 7).

### EgHD-Zip gene expression profiles in response to temperature

**Fig. 8:**
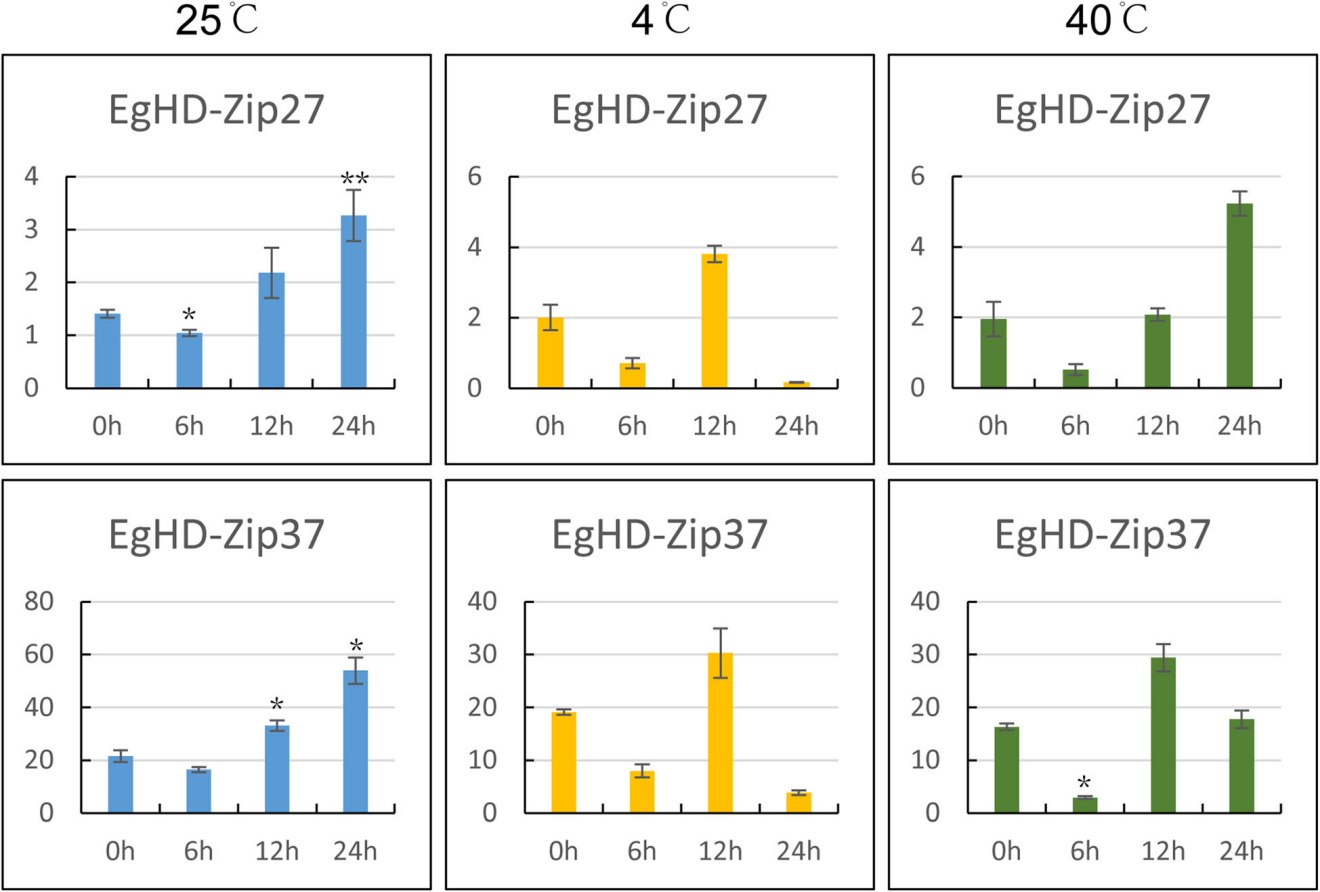
RT-PCR expression profiles of five subfamilies of genes in HD-Zip in response to temperature stress. The RNA was isolated from the young leaves of the treated *Eucalyptus* annual seedlings. The low temperature was treated with 4 °C, the high temperature was treated with 40 °C, and the control group was treated with 25 °C. The processing time of each group was 0 h, 6 h, 12 h and 24 h

At 4 °C, the expression of both EgHD-Zip27 and EgHD-Zip37 genes was lower than that at 25 °C, especially the expression of EgHD-Zip37 gene decreased by half at 4 °C. At 40 °C, the expression of EgHD-Zip37 was also reduced by half, so it can be speculated that EgHD-Zip37 is inhibited by high temperature and low temperature. The expression pattern of EgHD-Zip27 at 40 °C was the same as at 25 °C (Fig. 8).

## Discussion

The growth and development of plants are an extremely complicated process. In this process, plants are subjected to various biotic and abiotic stresses. To deal with these stresses, plants show physiological and biochemical regulation mechanisms. Plants sense and transmit the stress signals which regulate the expression of genes [63, 64]. HD-Zip proteins are plant-specific transcription factors that play a significant role in plant development and response against various stresses [8, 18, 24, 65]. In this study, a comprehensive identification and analysis of EgHD-Zip genes from *Eucalyptus* were performed. The results suggest that the HD-Zip family can be divided into four subfamilies, HD-ZipI-IV [24]. Multiple sequence alignments of and phylogenetic tree showed a high homology among these genes.

Conserved domain analysis suggests that all EgHD-Zip proteins except EgHD-Zip30 have motifs 1, 2, and 5. SMART analysis indicates that these three motifs represent the HD domain and LZ domain. The study of subfamily III and subfamily IV showed that both have motifs encoding HD, LZ, START, and SAD domains. In contrast, subfamily IV does not have motifs 8 and 4 that encode MEKHLA domains. Previous studies have shown that subfamily III is found in terrestrial plants. It is also worth noting that the MEKHLA domain was formed before the emergence of terrestrial plants, which could be the “preliminary preparation” for plants to adapt the terrestrial living conditions. Therefore, it can be speculated that subfamilies III and IV have the most recent comment ancestor. The analysis of gene structure showed that the exon-intron arrangement of different subfamilies has significant differences, while the same subfamily has similar structures and exon-intron numbers. But in the evolution process, exons would be lost or increased, and the exon length of each gene may also change. We found that HD-Zip35 lost an intron and HD-Zip7 and HD-Zip6 obtained an intron during the evolution, which could be one of the factors affecting the different functions of homologous genes.

The expression heat map analysis showed that the subfamily I has highly expressed, or even the highest expressed genes in the five tissues studied. It is speculated that they may be playing a significant role in the growth and development of *Eucalyptus*. In addition to the low expression level of subfamily II in mature leaves, the other four tissues have high expression genes, indicating that this family plays an essential role in the growth and transport of *Eucalyptus*. In the III subfamily, all genes are expressed in vascular bundle tissues, with high expression levels, but low expression in leaf and stem tips. Therefore, it can be speculated that they play an important role in *Eucalyptus* auxin transport and vascular tissue formation.

Interestingly, the expressions of the IV and III subfamilies were opposite in the five selected tissues. It is worth noting that HD-Zip38 was not expressed in the five selected samples, which does not mean that it is not important in the growth and development of *Eucalyptus*. On the contrary, it may be expressed explicitly in other tissues, thereby affecting the growth and development of *Eucalyptus* (Fig. S3, S4, S5, S6, S7).

The expression of HD-Zip in the young leaves treated with distilled water showed a trend of first decreasing and then increasing. After 6 h under salt stress, the EgHD-Zip gene showed some physiological activity, the expression first increased, then it dropped, followed by an increase again with the lowest expression level after 12 h of stress. It can be speculated that under moderate salt stress, the expression of HD-Zip gene is suppressed in young leaves of *Eucalyptus.*

**Fig. 9:**
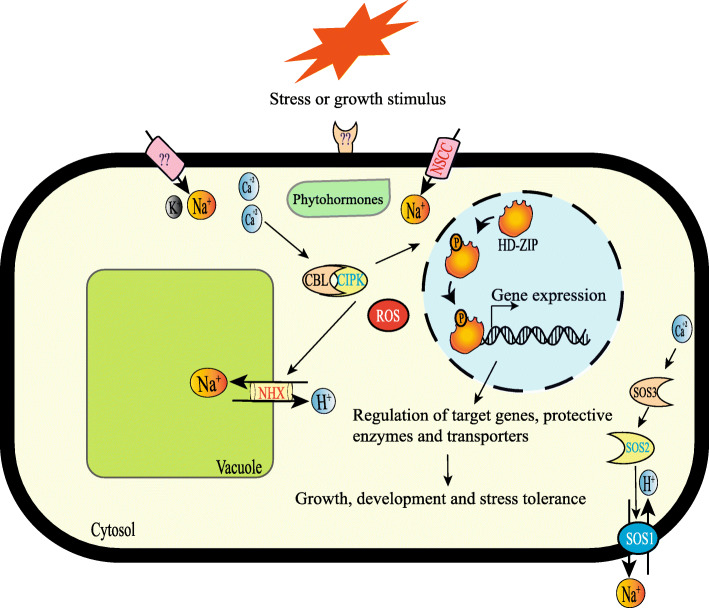
Working model of HD-Zip during developmental and stress (salt) response. The unknown receptor present on the plasma membrane perceives the stress or growth signal. Phytohormone, stress, or a change in the concentration of cytosolic Ca^2+^ may serve as the signal for the expression of HD-Zip transcription factors, which in turn regulate the expression of target genes resulting in growth, development and stress tolerance. In the case of salt exposure, the Na^+^ enters inside non-selective cation channels (NSCC) and some other transporters. Change in the concentration of cytosolic Ca^2+^ activates the CBL-CIPK signaling pathway leading to the increased activity of Na^+^/H^+^ antiporter SOS1 (salt overly sensitive1), which transport the Na^+^ in the vacuole or outside the cell. Phytohormone ABA levels also regulate the Na^+^/H^+^ antiporter NHX1 and other ion transporters to overcome with stress

The expression pattern from two selected genes, EgHD-Zip27 from HD-ZipII subfamily and EgHD-Zip37 from HD-ZipI subfamily, suggest that both the subfamilies play an essential role in coping with salt stress, and play a role in the growth and development of leaves (Fig. 9).

Under low-temperature stress, the expression pattern of HD-Zip gene mostly decreased first, then increased and decreased again. Under high-temperature stress, the expression of HD-Zip27 was the same as that under 25 °C treatment. At the same time, HD-Zip37 shows a pattern of first decreasing, then increasing and then decreasing again, suggesting its regulation by temperature change.

## Conclusion

HD-Zip family plays a vital role in the growth and development of plants. Here, a total of 40 EgHD-Zip genes were identified in *Eucalyptus*. Based on the evolutionary analysis, the EgHD-Zip family of *Eucalyptus* was divided into four groups. Moreover, genes that are in the same group tend to have similar exon-intron structures, motifs, and protein structures. During exposure to salt and temperature stresses, the expression of HD-Zip genes in *Eucalyptus* show differential expression. We here show that HD-Zip may play a unique role under different stress conditions. This study provides a basis for further research on the functional characterization of the *Eucalyptus* HD-Zip genes. It also provides a theoretical basis for other scholars to study the response of the *Eucalyptus* HD-Zip transcription factor during abiotic stress. As the land salinization is becoming a severe threat, further research for the improvement of *Eucalyptus* varieties could help in the expansion of *Eucalyptus* planting zones.

## Methods

### *Eucalyptus* HD-zip transcription factor family member: identification and chromosomal localization

HD-Zip protein sequences from *Arabidopsis* and rice were used as query in the TAIR database (http://www.arabidopsis.org) and PlantTFDB v4.0 database (Plant Transcription Factor Database). Also, the *Eucalyptus* HD-Zip HMM search [66] was carried out to integrate all the protein sequences obtained, in conjunction with SMART (Simple Molecular Agricultural Research Tool, http://smart.embl-heidelberg.de/) and EMBL pfam (https://pfam.xfam.org/) database was used to detect the conserved domains. Finally, the wrong sequence was removed by manual screening. The 40 candidate sequences after the screenings were considered to be the *Eucalyptus* HD-Zip gene (EgHD-Zip). The chromosomal locations of the EgHD-Zip genes were obtained from the genomic annotation information of phytozome v12.1.6 (https://phytozome.jgi.doe.gov/pz/portal.html), and using the Mapchart software it was visualized.

### Analysis of phylogeny and gene duplication of *Eucalyptus* HD-Zips

Multiple sequence alignments of *Eucalyptus*, rice and *Arabidopsis* HD-Zip protein sequences were performed using MUSCLE v3.8.31 [67]. The IQ-Tree software was built using the Maximum Likelihood (ML) method to construct a phylogenetic tree with the bootstrap option *n* = 1000. After that, the results were imported to iTOL (https://itol.embl.de/) online software for processing.

### Protein conserved motif and gene structure analysis

The Gene Structure Display Server (GSDS) [68, 69] was used to display the exon-intron of the *Eucalyptus* HD-Zip gene. We downloaded the annotation file in the format of GFF from Phytozome and retrieved the annotation information of the HD-Zip gene. The annotation information was uploaded to GSDS according to the default parameters of the exon-intron structure of the HD-Zip gene. Multiple Em for Motif Elicitation (MEME) was used to further determine the composition of motifs that might not be recorded in the public database [70, 71]. The width was kept 100aa, the number of motifs was 25, and other parameters were set to default values. Finally, based on the protein homology model in the swiss model database, we also inferred the 3D structure of the HD-Zip protein of *Eucalyptus*.

### Analysis of EgHD-Zip expression pattern

RNA-Seq data were downloaded from public websites and Phytozome [72]. Among them, the *Eucalyptus* RNA-Seq data included the following tissues: immature xylem, mature leaf, phloem, shoot tips, xylem. The heat maps of EgHD-Zip expression profiles from different tissues were obtained using TBtools software [73].

### Plant materials and growth conditions

The experimental material, *Eucalyptus grandis* clone Eg5, was collected from the College of Forestry, Fujian Agriculture and Forestry University. *Eucalyptus grandis* plants were grown on local soil for ten months under outdoor conditions. The annual average temperature in the growing area ranged from 16 to 20 °C, the yearly average rainfall from 900 to 2100 mm, and the annual relative humidity was about 77%. The red soil, which has the organic matter content from 2.57 to 6.07%, and the pH value 5, was used for cultivation.

### Expression profile of EgHD-Zip gene under temperature and salt stress

Annual *Eucalyptus* seedlings were treated with 100 mmol/L sodium chloride and 200 mmol/L sodium chloride for 0, 6, 12, and 24 h for salt stress, and the control seedlings were treated with distilled water. For the temperature stress, *Eucalyptus* seedlings were placed at 4 °C and 40 °C, respectively, while and the control group was kept at room temperature (at 25 °C) for 0, 6, 12, and 24 h. After the stress, the samples were stored at − 80 °C for the subsequent analysis. For each treatment, five different seedlings were used, and the experiment was repeated at least three times.

### Quantitative RT-PCR (qRT-PCR)

Total RNA was extracted using RNA extraction Kit (Omega Bio-Tek, Shanghai, China) from control and stress treated samples. cDNA was synthesized with the *EasyScript*® One-Step gDNA Removal and cDNA Synthesis SuperMix following the manufacturer’s protocol (Transgen, Beijing, China).and qRT-qPCR was conducted using TransStart® Top Green qPCR SuperMix (Transgen, Beijing, China). As a reference gene actin was used. Relative transcript abundance was calculated using the comparative 2^–ΔΔC^_T_ method [74]. All experiments were performed using three biological replicates and three technical replicates. The primers used in the study are listed in the supplementary Table S1.

### Statistical analysis

A two-tailed Student’s t-test was applied to find statistical significance. Results are depicted as the mean values ± SE of three biological replicates.

## Supplementary information

**Additional file 1: Figure S1.** Phylogenetic classification of HD-Zip gene in *Eucalyptus grandis*, *Arabidopsis thaliana* and *Oryza sativa*.

**Additional file 2: Figure S2.** Phylogenetic classification of HD-Zip gene in *Eucalyptus grandis*

**Additional file 3: Figure S3.** The motif composition of HD-Zip proteins.

**Additional file 4: Figure S4.** Sequence information of each motif identified by MEME.

**Additional file 5: Figure S5.** Relative expression levels of EgHD-ZipI genes in various tissues.

**Additional file 6: Figure S6.** Relative expression levels of EgHD-Zip II genes in various tissues.

**Additional file 7: Figure S7.** Relative expression levels of EgHD-Zip III genes in various tissues.

**Additional file 8: Figure S8.** Relative expression levels of EgHD-Zip IV genes in various tissues.

**Additional file 9: Table S1.** Details of primers used in this study.

**Additional file 10: Table S2.** The expression profile of pineapple bHLH genes in different tissue and developmental stages.

**Additional file 11: Table S3.** Eucalyptus HD-Zip sequences used in current study.

## Data Availability

All the data and materials that are required to reproduce these findings can be shared by contacting the corresponding author. All data generated or analysed during this study are included in this published article as supplementary file S10 and S11. The datasets analysed during the current study are available in the NCBI SRA database with the SRA accession code: PRJNA30415 and PRJNA223526. The SRA record is accessible with the following link: https://www.ncbi.nlm.nih.gov/sra/PRJNA30415 and https://www.ncbi.nlm.nih.gov/sra/SRX367258.

## References

[1] Franco-Zorrilla JM, Solano R (2017). Identification of plant transcription factor target sequences. Bba-Gene Regul Mech.

[2] Qiang L, Guiyou Z, Shouyi C (2001). Structure and regulatory function of plant transcription factors. Chin Sci Bull.

[3] Golldack D, Luking I, Yang O (2011). Plant tolerance to drought and salinity: stress regulating transcription factors and their functional significance in the cellular transcriptional network. Plant Cell Rep.

[4] Nakashima K, Ito Y, Yamaguchi-Shinozaki K (2009). Transcriptional regulatory networks in response to abiotic stresses in Arabidopsis and grasses. Plant Physiol.

[5] Li YY, Bai BC, Wen F, Zhao M, Xia QY, Yang DH, Wang GH. Genome-Wide Identification and Expression Analysis of HD-ZIP I Gene Subfamily in *Nicotiana tabacum*. Genes-Basel. 2019;10(8):575. 10.3390/genes10080575 PMC6723700 31366162

[6] Wang HY, Wang HL, Shao HB, Tang XL (2016). Recent advances in utilizing transcription factors to improve plant abiotic stress tolerance by transgenic technology. Front Plant Sci.

[7] Ariel FD, Manavella PA, Dezar CA, Chan RL (2007). The true story of the HD-zip family. Trends Plant Sci.

[8] Henriksson E, Olsson AS, Johannesson H, Johansson H, Hanson J, Engstrom P, Soderman E (2005). Homeodomain leucine zipper class I genes in *Arabidopsis*. Expression patterns and phylogenetic relationships. Plant Physiol.

[9] Viola IL, Gonzalez DH: Chapter 6 - Structure and Evolution of Plant Homeobox Genes. In: Plant Transcription Factors. Edited by Gonzalez DH. Boston: Academic Press; 2016. p. 101–12.

[10] Agalou A, Purwantomo S, Overnas E, Johannesson H, Zhu X, Estiati A, de Kam RJ, Engstrom P, Slamet-Loedin IH, Zhu Z (2008). A genome-wide survey of HD-zip genes in rice and analysis of drought-responsive family members. Plant Mol Biol.

[11] Chen X, Chen Z, Zhao HL, Zhao Y, Cheng BJ, Xiang Y (2014). Genome-wide analysis of soybean hd-zip gene family and expression profiling under salinity and drought treatments. PLoS One.

[12] Li ZQ, Zhang C, Guo YR, Niu WL, Wang Y, Xu Y (2017). Evolution and expression analysis reveal the potential role of the HD-Zip gene family in regulation of embryo abortion in grapes (*Vitis vinifera* L.). BMC Genomics.

[13] Mao HD, Yu LJ, Li ZJ, Liu H, Han R (2016). Molecular evolution and gene expression differences within the HD-zip transcription factor family of Zea mays L. Genetica.

[14] Yue H, Shu D, Wang M, Xing G, Zhan H, Du X, Song W, Nie X (2018). Genome-Wide Identification and Expression Analysis of the HD-Zip Gene Family in Wheat (*Triticum aestivum* L.). Genes (Basel).

[15] Zhang Z, Chen X, Guan X, Liu Y, Chen H, Wang T, Mouekouba LD, Li J, Wang A (2014). A genome-wide survey of homeodomain-leucine zipper genes and analysis of cold-responsive HD-zip I members' expression in tomato. Biosci Biotechnol Biochem.

[16] Li Z, Gao Z, Li R, Xu Y, Hu R (2020). Genome-wide identification and expression profiling of HD-ZIP gene family in *Medicago truncatula*. Genomics.

[17] Yang Y-Y, Shan W, Kuang J-F, Chen J-Y, Lu W-J (2019). Four HD-ZIPs are involved in banana fruit ripening by activating the transcription of ethylene biosynthetic and cell wall-modifying genes. Plant Cell Rep.

[18] Ariel F, Diet A, Verdenaud M, Gruber V, Frugier F, Chan R, Crespi M (2010). Environmental regulation of lateral root emergence in Medicago truncatula requires the HD-zip I transcription factor HB1. Plant Cell.

[19] Aoyama T, Dong CH, Wu Y, Carabelli M, Sessa G, Ruberti I, Morelli G, Chua NH (1995). Ectopic expression of the *Arabidopsis* transcriptional activator Athb-1 alters leaf cell fate in tobacco. Plant Cell.

[20] Harris JC, Hrmova M, Lopato S, Langridge P (2011). Modulation of plant growth by HD-zip class I and II transcription factors in response to environmental stimuli. New Phytol.

[21] Meijer AH, de Kam RJ, d'Erfurth I, Shen W, Hoge JH (2000). HD-zip proteins of families I and II from rice: interactions and functional properties. Mol Gen Genet.

[22] Cote CL, Boileau F, Roy V, Ouellet M, Levasseur C, Morency MJ, Cooke JEK, Seguin A, MacKay JJ (2010). Gene family structure, expression and functional analysis of HD-zip III genes in angiosperm and gymnosperm forest trees. BMC Plant Biol.

[23] Pandey A, Misra P, Alok A, Kaur N, Sharma S, Lakhwani D, Asif MH, Tiwari S, Trivedi PK (2016). Genome-wide identification and expression analysis of Homeodomain Leucine zipper subfamily IV (HDZ IV) gene family from *Musa accuminata*. Front Plant Sci.

[24] Elhiti M, Stasolla C (2009). Structure and function of homodomain-leucine zipper (HD-zip) proteins. Plant Signal Behav.

[25] Yang Y, HHJ A, Harris J, Riboni M, Kovalchuk N (2020). DREB/CBF expression in wheat and barley using the stress-inducible promoters of HD-zip I genes: impact on plant development, stress tolerance and yield. Plant Biotechnol J.

[26] Capella M, Ribone PA, Arce AL, Chan RL (2015). Arabidopsis thaliana HomeoBox 1 (AtHB1), a Homedomain-Leucine zipper I (HD-zip I) transcription factor, is regulated by PHYTOCHROME-INTERACTING FACTOR 1 to promote hypocotyl elongation. New Phytol.

[27] Valdes AE, Overnas E, Johansson H, Rada-Iglesias A, Engstrom P (2012). The homeodomain-leucine zipper (HD-zip) class I transcription factors ATHB7 and ATHB12 modulate abscisic acid signalling by regulating protein phosphatase 2C and abscisic acid receptor gene activities. Plant Mol Biol.

[28] Manavella PA, Arce AL, Dezar CA, Bitton F, Renou JP, Crespi M, Chan RL (2006). Cross-talk between ethylene and drought signalling pathways is mediated by the sunflower Hahb-4 transcription factor. Plant J.

[29] Yang YF, Luang S, Harris J, Riboni M, Li Y, Bazanova N, Hrmova M, Haefele S, Kovalchuk N, Lopato S (2018). Overexpression of the class I homeodomain transcription factor TaHDZipI-5 increases drought and frost tolerance in transgenic wheat. Plant Biotechnol J.

[30] Zhao Y, Ma Q, Jin X, Peng X, Liu J, Deng L, Yan H, Sheng L, Jiang H, Cheng B (2014). A novel maize homeodomain-leucine zipper (HD-zip) I gene, Zmhdz10, positively regulates drought and salt tolerance in both rice and Arabidopsis. Plant Cell Physiol.

[31] Li S, Chen N, Li F, Mei F, Mao H (2020). Characterization of wheat homeodomain-leucine zipper family genes and functional analysis of TaHDZ5-6A in drought tolerance in transgenic Arabidopsis. BMC Plant Biol.

[32] Mou S, Liu Z, Gao F, Yang S, Su M, Shen L, Wu Y, He S (2017). CaHDZ27, a Homeodomain-Leucine zipper I protein, positively regulates the resistance to *Ralstonia solanacearum* infection in pepper. Mol Plant-Microbe Interact.

[33] Franklin KA, Praekelt U, Stoddart WM, Billingham OE, Halliday KJ, Whitelam GC (2003). Phytochromes B, D, and E act redundantly to control multiple physiological responses in *Arabidopsis*. Plant Physiol.

[34] Carabelli M, Turchi L, Ruzza V, Morelli G, Ruberti I (2013). Homeodomain-Leucine zipper II family of transcription factors to the limelight: central regulators of plant development. Plant Signal Behav.

[35] Park MY, Kim SA, Lee SJ, Kim SY (2013). ATHB17 is a positive regulator of abscisic acid response during early seedling growth. Mol Cells.

[36] Soderman E, Hjellstrom M, Fahleson J, Engstrom P (1999). The HD-zip gene ATHB6 in Arabidopsis is expressed in developing leaves, roots and carpels and up-regulated by water deficit conditions. Plant Mol Biol.

[37] Rueda EC, Dezar CA, Gonzalez DH, Chan RL (2005). Hahb-10, a sunflower homeobox-leucine zipper gene, is regulated by light quality and quantity, and promotes early flowering when expressed in *Arabidopsis*. Plant Cell Physiol.

[38] Sessa G, Steindler C, Morelli G, Ruberti I (1998). The *Arabidopsis* Athb-8, −9 and −14 genes are members of a small gene family coding for highly related HD-ZIP proteins. Plant Mol Biol.

[39] Zhu YY, Song DL, Xu P, Sun JY, Li LG (2018). A HD-ZIP III gene, PtrHB4, is required for interfascicular cambium development in Populus. Plant Biotechnol J.

[40] Hawker NP, Bowman JL (2004). Roles for class III HD-zip and KANADI genes in Arabidopsis root development. Plant Physiol.

[41] Prigge MJ, Otsuga D, Alonso JM, Ecker JR, Drews GN, Clark SE (2005). Class III homeodomain-leucine zipper gene family members have overlapping, antagonistic, and distinct roles in *Arabidopsis* development. Plant Cell.

[42] Robischon M, Du J, Miura E, Groover A (2011). The Populus class III HD ZIP, popREVOLUTA, influences cambium initiation and patterning of woody stems. Plant Physiol.

[43] Bartel DP (2004). MicroRNAs: genomics, biogenesis, mechanism, and function. Cell.

[44] Voinnet O (2009). Origin, biogenesis, and activity of plant microRNAs. Cell.

[45] Carlsbecker A, Lee JY, Roberts CJ, Dettmer J, Lehesranta S, Zhou J, Lindgren O, Moreno-Risueno MA, Vaten A, Thitamadee S (2010). Cell signalling by microRNA165/6 directs gene dose-dependent root cell fate. Nature.

[46] Emery JF, Floyd SK, Alvarez J, Eshed Y, Hawker NP, Izhaki A, Baum SF, Bowman JL (2003). Radial patterning of *Arabidopsis* shoots by class III HD-ZIP and KANADI genes. Curr Biol.

[47] Singh A, Roy S, Singh S, Das SS, Gautam V, Yadav S, Kumar A, Singh A, Samantha S, Sarkar AK (2017). Phytohormonal crosstalk modulates the expression of miR166/165s, target class III HD-ZIPs, and KANADI genes during root growth in *Arabidopsis thaliana*. Sci Rep.

[48] Merelo P, Ram H, Pia Caggiano M, Ohno C, Ott F, Straub D, Graeff M, Cho SK, Yang SW, Wenkel S (2016). Regulation of MIR165/166 by class II and class III homeodomain leucine zipper proteins establishes leaf polarity. Proc Natl Acad Sci U S A.

[49] Singh A, Singh S, Panigrahi KC, Reski R, Sarkar AK (2014). Balanced activity of microRNA166/165 and its target transcripts from the class III homeodomain-leucine zipper family regulates root growth in Arabidopsis thaliana. Plant Cell Rep.

[50] Williams L, Grigg SP, Xie M, Christensen S, Fletcher JC (2005). Regulation of Arabidopsis shoot apical meristem and lateral organ formation by microRNA miR166g and its AtHD-ZIP target genes. Development.

[51] Tron AE, Bertoncini CW, Palena CM, Chan RL, Gonzalez DH (2001). Combinatorial interactions of two amino acids with a single base pair define target site specificity in plant dimeric homeodomain proteins. Nucleic Acids Res.

[52] Chew W, Hrmova M, Lopato S (2013). Role of Homeodomain Leucine zipper (HD-zip) IV transcription factors in plant development and plant protection from deleterious environmental factors. Int J Mol Sci.

[53] Ingram GC, Boisnard-Lorig C, Dumas C, Rogowsky PM (2000). Expression patterns of genes encoding HD-ZipIV homeo domain proteins define specific domains in maize embryos and meristems. Plant J.

[54] Nakamura M, Katsumata H, Abe M, Yabe N, Komeda Y, Yamamoto KT, Takahashi T (2006). Characterization of the class IV homeodomain-Leucine zipper gene family in Arabidopsis. Plant Physiol.

[55] Ito M, Sentoku N, Nishimura A, Hong SK, Sato Y, Matsuoka M (2002). Position dependent expression of GL2-type homeobox gene, Roc1: significance for protoderm differentiation and radial pattern formation in early rice embryogenesis. Plant J.

[56] Ingouff M, Farbos I, Lagercrantz U, von Arnold S (2001). PaHB1 is an evolutionary conserved HD-GL2 homeobox gene expressed in the protoderm during Norway spruce embryo development. Genesis.

[57] Medbouhi A, Benbelaid F, Djabou N, Beaufay C, Bendahou M, Quetin-Leclercq J, Tintaru A, Costa J, Muselli A (2019). Essential oil of Algerian Eryngium campestre: chemical variability and evaluation of biological activities. Molecules.

[58] Silva J, Abebe W, Sousa SM, Duarte VG, Machado MI, Matos FJ (2003). Analgesic and anti-inflammatory effects of essential oils of Eucalyptus. J Ethnopharmacol.

[59] de Sá-Martins R, Cleiton-José A, Rocha-Faria JM, de Melo LA (2019). Effect of water and salt stress on seeds germination and vigor of different eucalyptus species. J Trop For Sci.

[60] Coscolin RBS, Broetto F, Marchese JA, Campohermoso MC, Paladini MV (2011). Effects of hydric deficiency on gas exchange parameters and metabolism of Eucalyptus grandis clones. Braz J Plant Physiol.

[61] Myburg AA, Grattapaglia D, Tuskan GA, Hellsten U, Hayes RD, Grimwood J, Jenkins J, Lindquist E, Tice H, Bauer D (2014). The genome of Eucalyptus grandis. Nature.

[62] Arce AL, Raineri J, Capella M, Cabello JV, Chan RL (2011). Uncharacterized conserved motifs outside the HD-zip domain in HD-zip subfamily I transcription factors; a potential source of functional diversity. BMC Plant Biol.

[63] Verslues PE, Agarwal M, Katiyar-Agarwal S, Zhu J, Zhu J-K (2006). Methods and concepts in quantifying resistance to drought, salt and freezing, abiotic stresses that affect plant water status. Plant J.

[64] Yamaguchi-Shinozaki K, Shinozaki K (2006). Transcriptional regulatory networks in cellular responses and tolerance to dehydration and cold stresses. Annu Rev Plant Biol.

[65] Wan L, Jieya D, Minxuan C, Xianxian G, Dongdong W (2019). Genome-wide identification and characterization of HD-ZIP genes in potato. Gene.

[66] Eddy SR (1998). Profile hidden Markov models. Bioinformatics.

[67] Edgar RC (2004). MUSCLE: a multiple sequence alignment method with reduced time and space complexity. BMC Bioinform.

[68] Guo AY, Zhu QH, Chen X, Luo JC (2007). GSDS: a gene structure display server. Yi Chuan.

[69] Hu B, Jin J, Guo AY, Zhang H, Luo J, Gao G (2015). GSDS 2.0: an upgraded gene feature visualization server. Bioinformatics.

[70] Bailey TL, Boden M, Buske FA, Frith M, Grant CE, Clementi L, Ren J, Li WW, Noble WS: MEME SUITE: tools for motif discovery and searching. Nucleic Acids Res 2009, 37(Web Server issue):W202–W208. 10.1093/nar/gkp335 PMC2703892 19458158

[71] Bailey TL, Elkan C (1994). Fitting a mixture model by expectation maximization to discover motifs in biopolymers. Proc Int Conf Intell Syst Mol Biol.

[72] Goodstein DM, Shu S, Howson R, Neupane R, Hayes RD, Fazo J, Mitros T, Dirks W, Hellsten U, Putnam N (2012). Phytozome: a comparative platform for green plant genomics. Nucleic Acids Res.

[73] Chen C, Chen H, Zhang Y, Thomas HR, Frank MH, He Y, Xia R (2020). TBtools: an integrative toolkit developed for interactive analyses of big biological data. Mol Plant.

[74] Livak KJ, Schmittgen TD (2001). Analysis of relative gene expression data using real-time quantitative PCR and the 2^−ΔΔ*C*^_T_ method. Methods.

