# Supplementary figures and images for: Genome-wide characterization and expression profiling of Eucalyptus grandis HD-Zip gene family in response to salt and temperature stress
Source: BMC Plant Biol. 2020 Oct 1;20:451. doi: 10.1186/s12870-020-02677-w (PMC7528242; doi:10.1186/s12870-020-02677-w)

**HD-ZIP I**

**HD-ZIP II**

**HD-ZIP III**

**HD-ZIP IV**

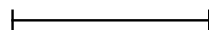

1

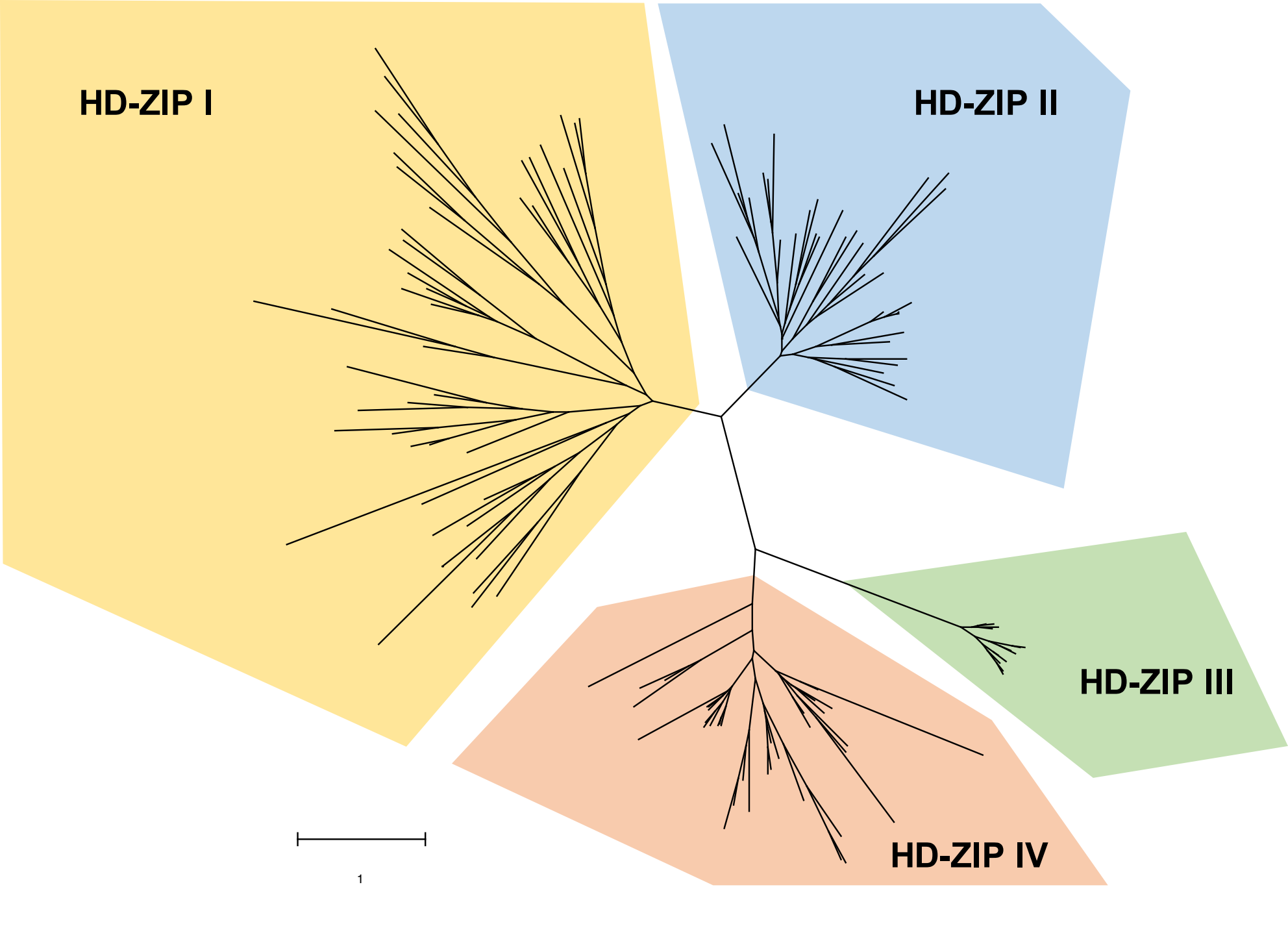

Supplement: Supplementary file 1 — Additional file 1: Figure S1. Phylogenetic classification of HD-Zip gene in Eucalyptus grandis, Arabidopsis thaliana and Oryza sativa. [file 12870_2020_2677_MOESM1_ESM.pdf]

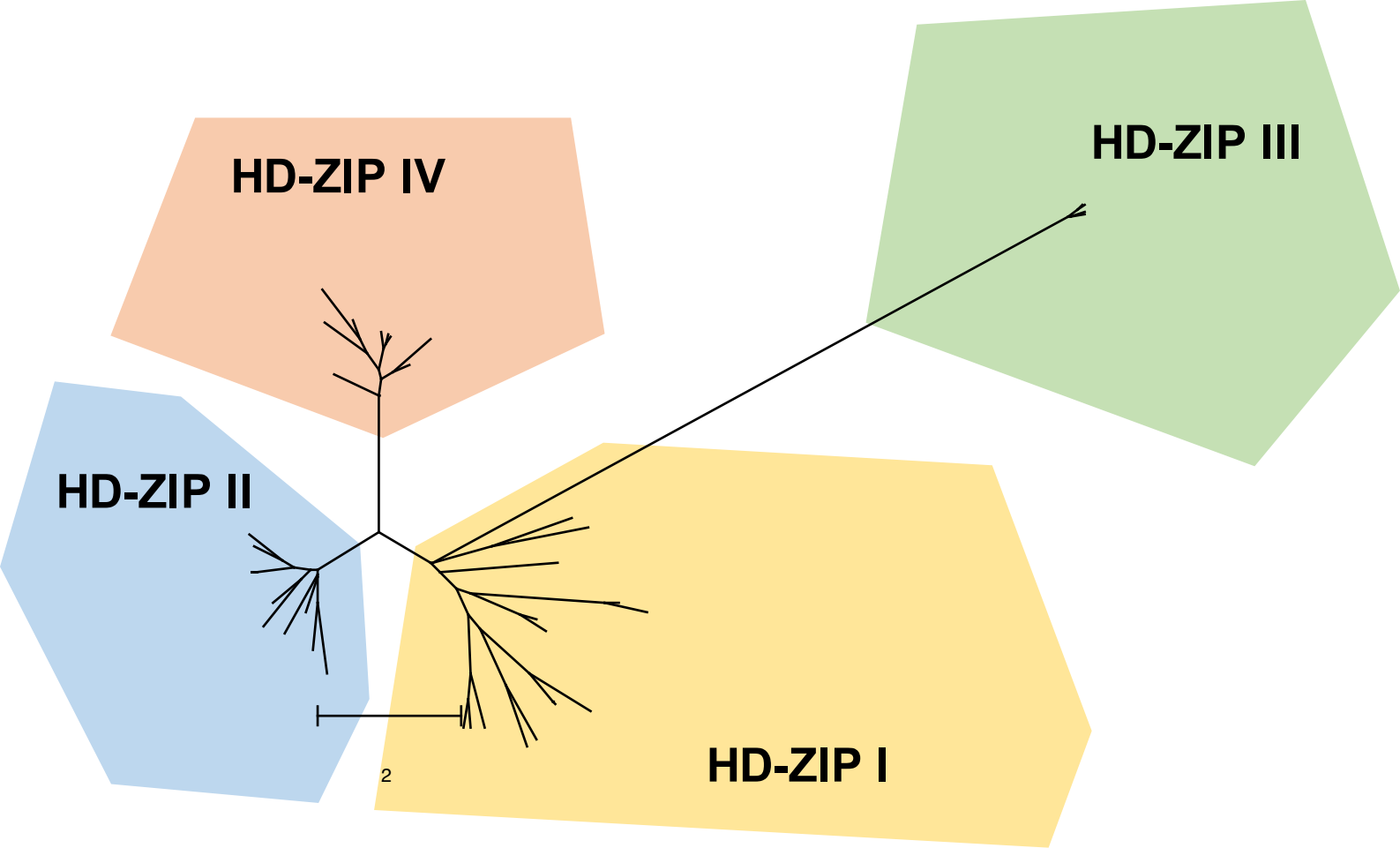

Supplement: Supplementary file 2 — Additional file 2: Figure S2. Phylogenetic classification of HD-Zip gene in Eucalyptus grandis [file 12870_2020_2677_MOESM2_ESM.pdf]

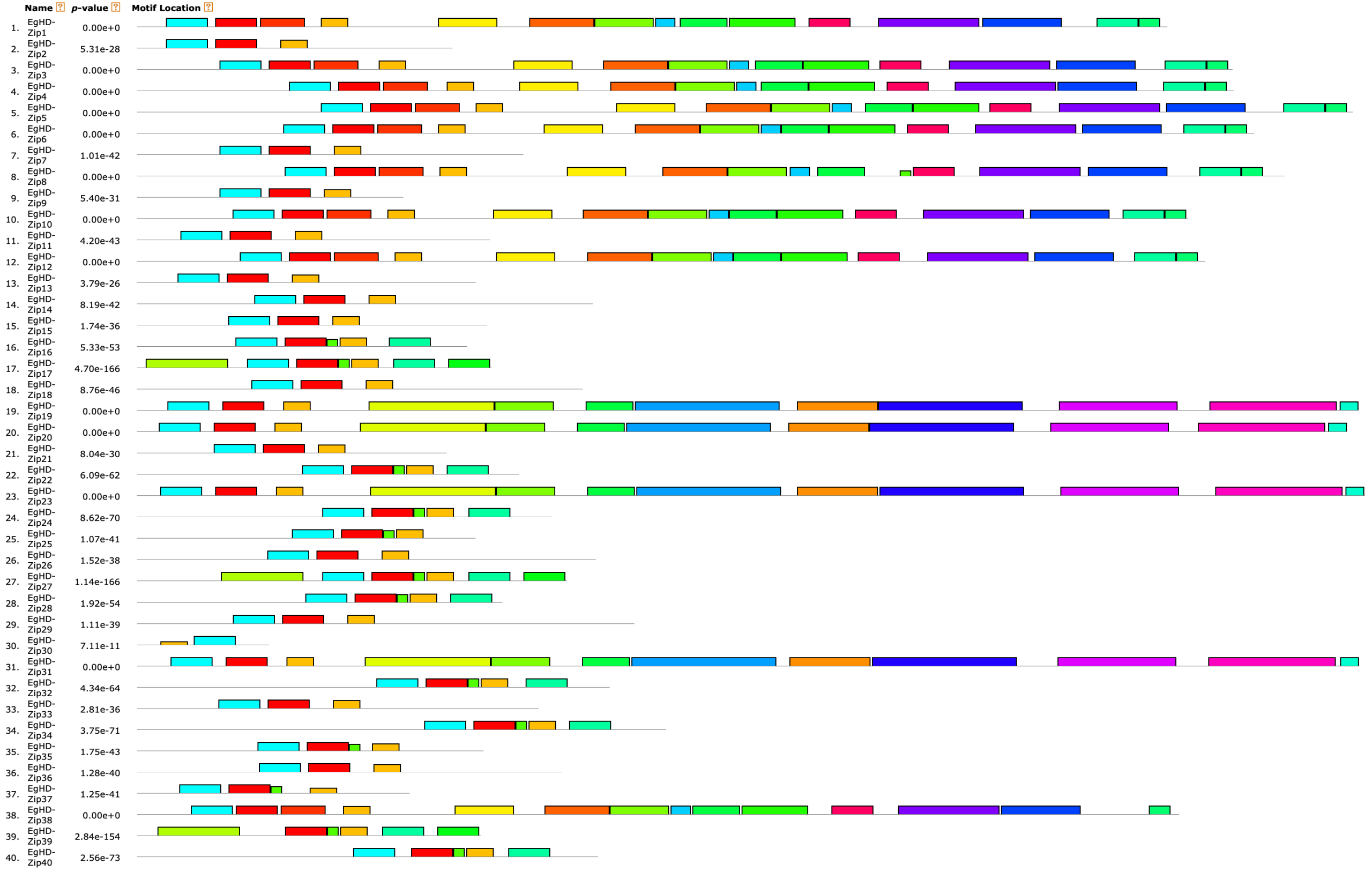

Supplement: Supplementary file 3 — Additional file 3: Figure S3. The motif composition of HD-Zip proteins. [file 12870_2020_2677_MOESM3_ESM.pdf]

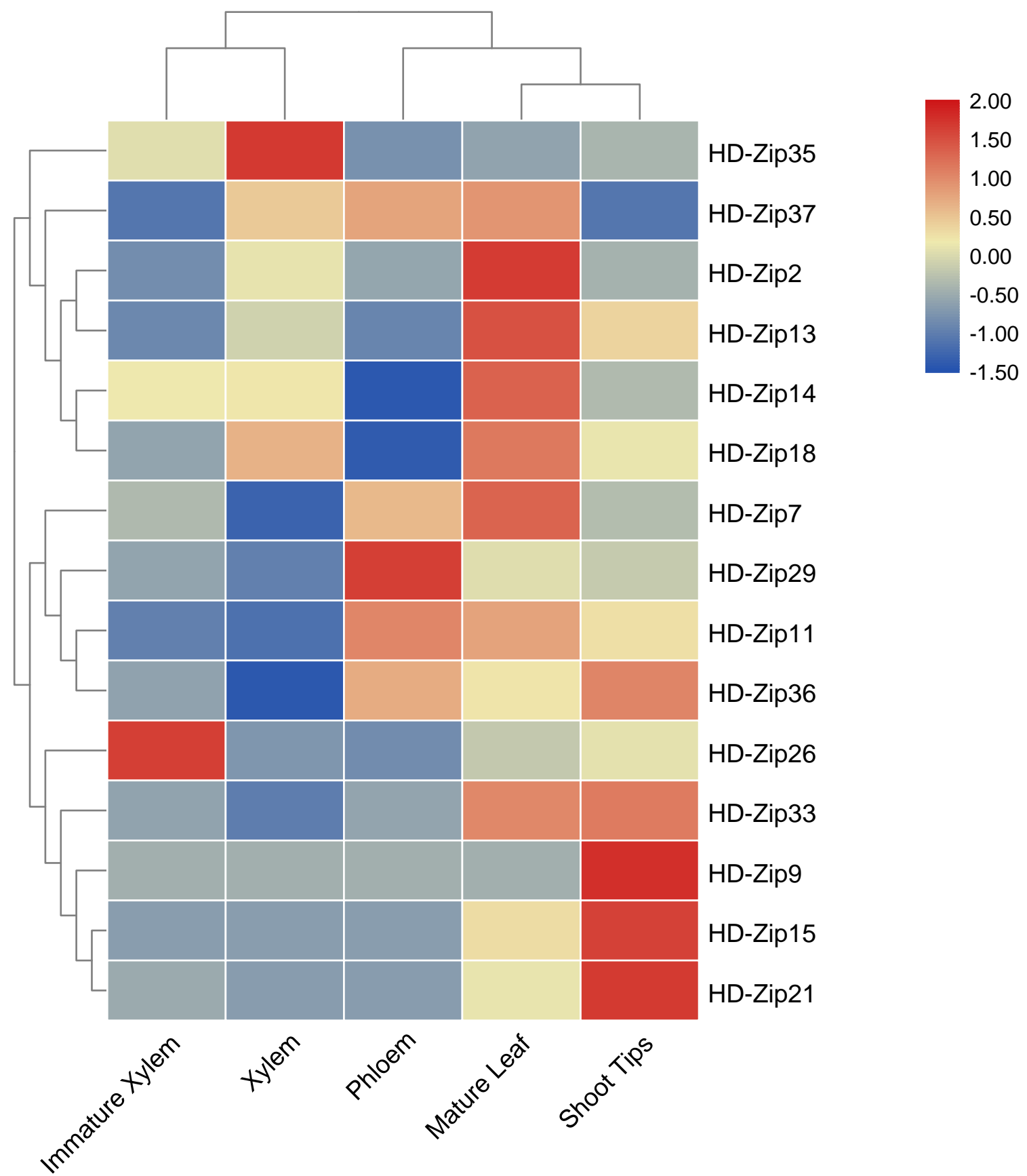

Supplement: Supplementary file 5 — Additional file 5: Figure S5. Relative expression levels of EgHD-ZipI genes in various tissues. [file 12870_2020_2677_MOESM5_ESM.pdf]

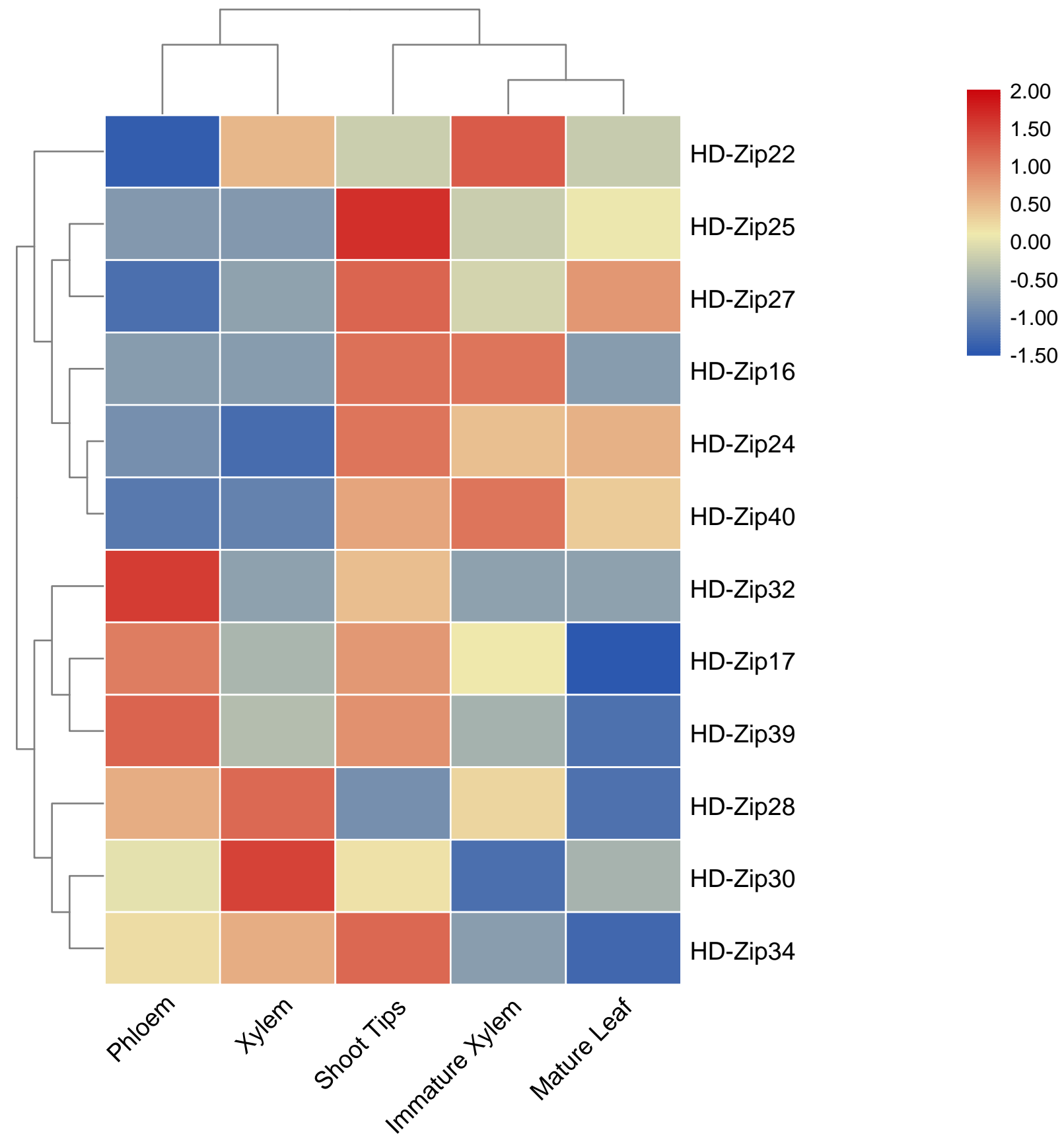

Supplement: Supplementary file 6 — Additional file 6: Figure S6. Relative expression levels of EgHD-Zip II genes in various tissues. [file 12870_2020_2677_MOESM6_ESM.pdf]

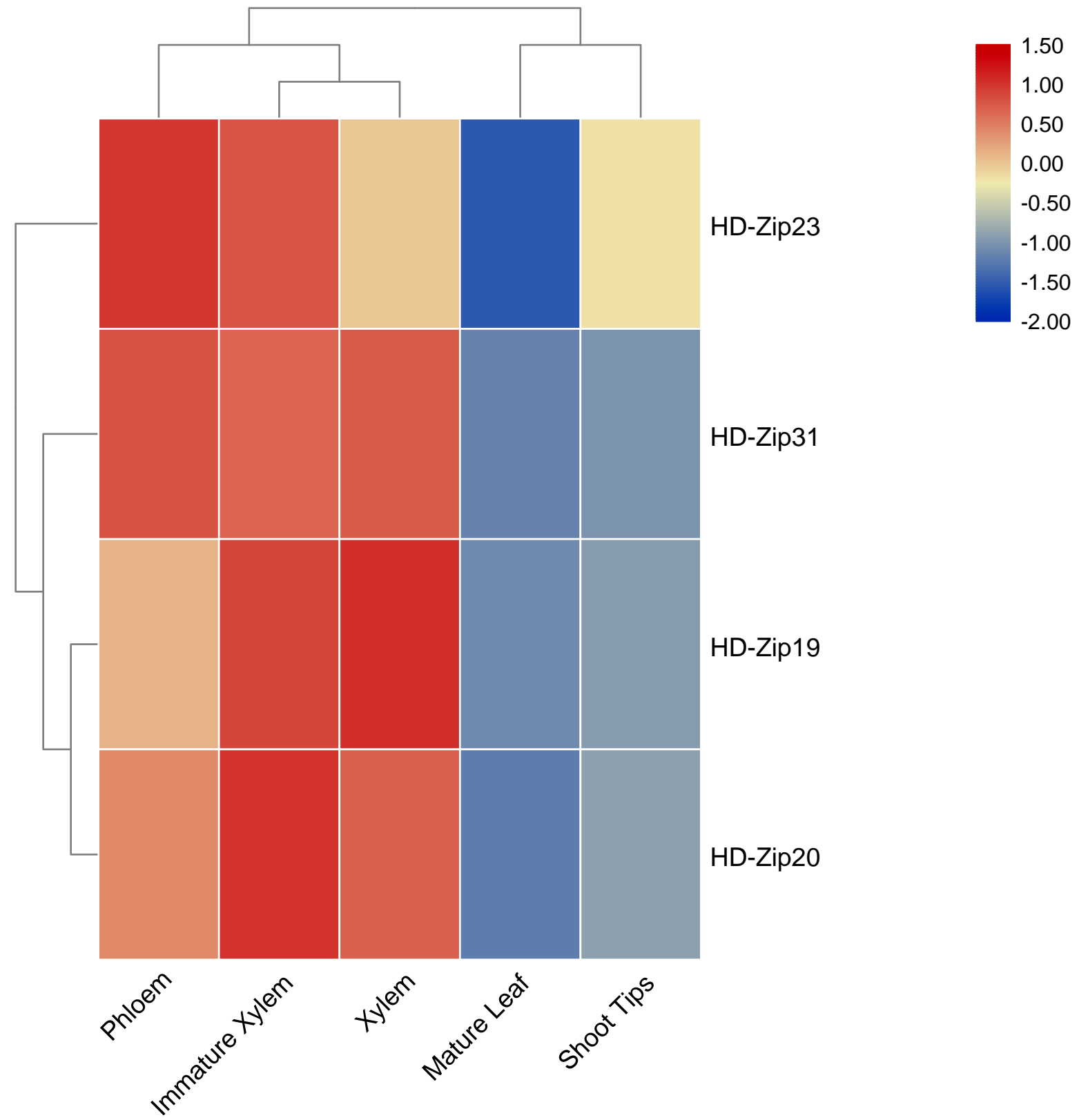

Supplement: Supplementary file 7 — Additional file 7: Figure S7. Relative expression levels of EgHD-Zip III genes in various tissues. [file 12870_2020_2677_MOESM7_ESM.pdf]

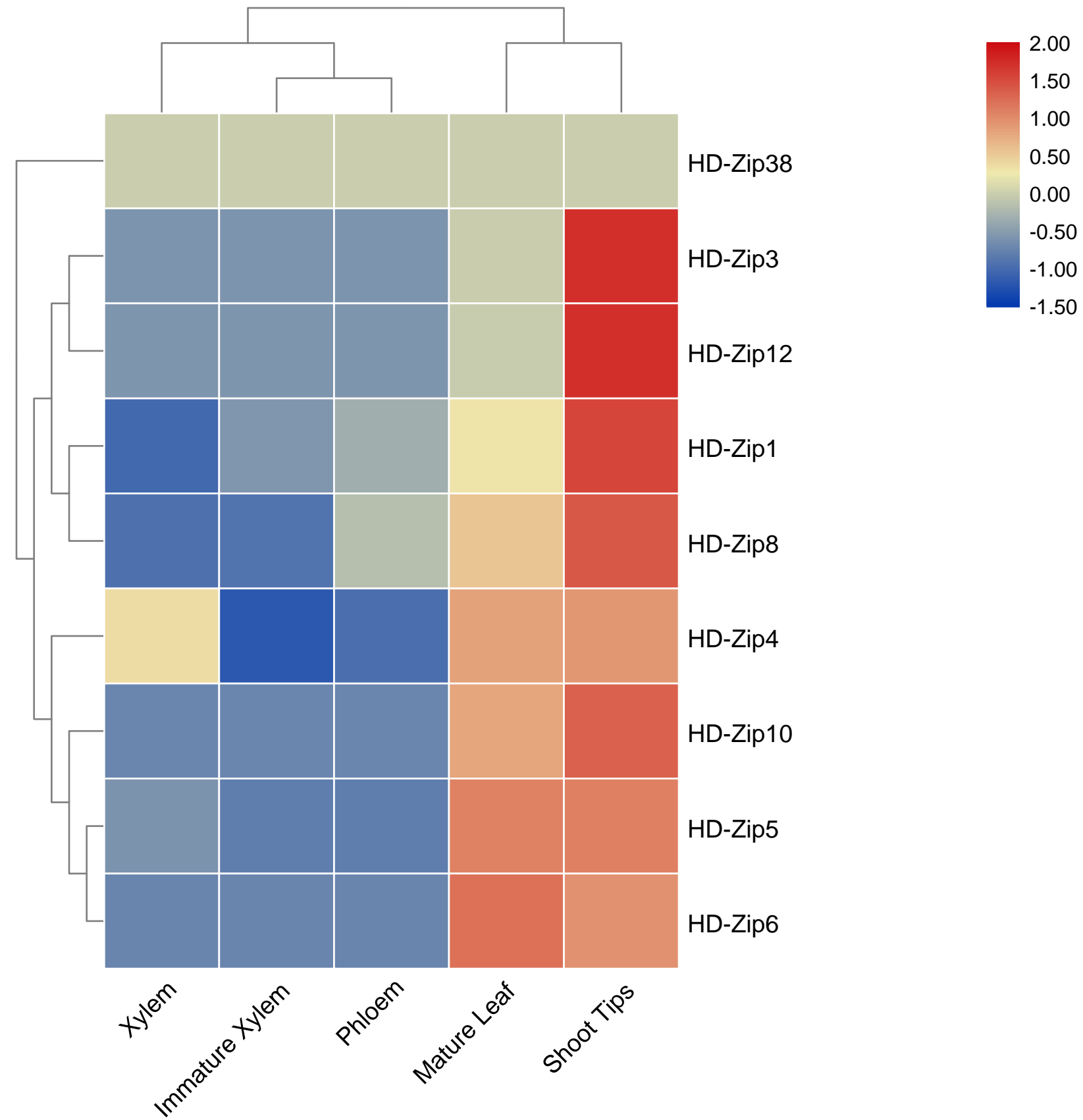

Supplement: Supplementary file 8 — Additional file 8: Figure S8. Relative expression levels of EgHD-Zip IV genes in various tissues. [file 12870_2020_2677_MOESM8_ESM.pdf]
