# Supplementary material for: Genome-wide characterization and expression profiling of Eucalyptus grandis HD-Zip gene family in response to salt and temperature stress
Source: BMC Plant Biol. 2020 Oct 1;20:451. doi: 10.1186/s12870-020-02677-w (PMC7528242; doi:10.1186/s12870-020-02677-w)
Supplement: Supplementary file 4 — Additional file 4: Figure S4. Sequence information of each motif identified by MEME. [file 12870_2020_2677_MOESM4_ESM.pdf]

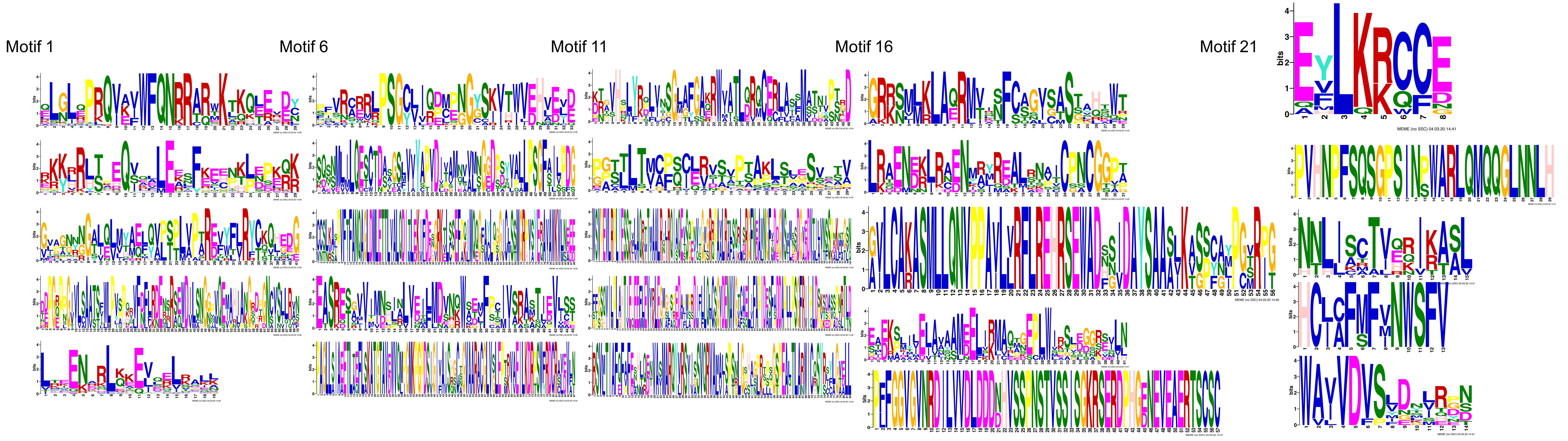

Motif 1
Motif 6
Motif 11
Motif 16
Motif 21
