# Supplementary material for: Genome-wide characterization and expression profiling of Eucalyptus grandis HD-Zip gene family in response to salt and temperature stress
Source: BMC Plant Biol. 2020 Oct 1;20:451. doi: 10.1186/s12870-020-02677-w (PMC7528242; doi:10.1186/s12870-020-02677-w)
Supplement: Supplementary file 9 — Additional file 9: Table S1. Details of primers used in this study. [file 12870_2020_2677_MOESM9_ESM.docx]

| Name | Sequence (5’-3’) |
| --- | --- |
| HD-Zip27-F | TCTTAGTCCCAGACAGGTAGAG |
| HD-Zip27-R | GATTGTCGCAGCACCTCTTA |
| HD-Zip37-F | GGACTACGAGGTGTTGAAGAAG |
| HD-Zip37-R | CTTCATTTGTGTCCCTTCCTTTG |
| Actin-F | TTACCGCGATCTGGATCTTTAC |
| Actin-R | CCTGCATAAGCCTCCTTCAA |

**Table S1: Details of primers used in this study.**
