# Supplementary material for: Genome-wide characterization and expression profiling of Eucalyptus grandis HD-Zip gene family in response to salt and temperature stress
Source: BMC Plant Biol. 2020 Oct 1;20:451. doi: 10.1186/s12870-020-02677-w (PMC7528242; doi:10.1186/s12870-020-02677-w)
Supplement: Supplementary file 11 — Additional file 11: Table S3. Eucalyptus HD-Zip sequences used in current study. [file 12870_2020_2677_MOESM11_ESM.docx]

**Table S3:** Eucalyptus HD-Zip sequences used in current study.

a) HD-Zip nucleotide sequences used in current study.

>EgHD-Zip1

ATGGATTTCGGTGGCGGTTCCGGCGGGGACCACGACGGTTCCGACCACCAGAAGAGGAAGAAGCGGTACCATCGCCACAC

TGCCCACCAGATTCAGAGGCTTGAAGCGATGTTCAGGGAATGCCCTCATCCAGATGAGAAGCAGCGGATGGAGTTGAGCC

GGGAATTGGGATTGGCTCCGAGACAGATCAAGTTTTGGTTCCAAAATAGGAGAACCCAGATGAAGGCCCAGCATGAAAGG

GCTGATAACTGTGCATTGAGGGCAGAAAACGATAGGATTCGATGCGAAAACATTGTGATAAGAGAGGCCTTGAGAAACGT

CATTTGCCCGAGTTGTGGCACCGTGCCGTCAGGCGAAGATTCCTACTTCGATGAACAAAAACTGCGGATGGAGAATGCAC

ATTTGAAAGAGGAGCTCGATCGAGTTTCTAGCATTGCGGCCAAGTACATCGGAAGGCCAATGTCGCAACTCCCACCTGTT

CAACCAATTCATATCGCTTCTCTGGATTTGACGATGAGTAGTCTTGGTGCTCATGGACTGGCAGGCCCTTCTCTTGACCT

TGATCTACTTCCGGGCAGCTCATCTTCTGTGCCACATTTGCCTTTTCAAGCAGTTGTTTTCTCGGACATGGATAAGTCCC

TAATGGCAGATATAGCAGCTAATGCTCTGGATGAATTTCTTAGGCTTGCTCAGACAGATGAACCTTTATGGATGAAATCT

ACTACCGATGGGAGGGACAATCTCAATCTCGAAAGTTACGAGAGAATGTTTCCTAGGGCTAGTAGTCACCTCAAAAATCC

AGATGTCCGTGTTGAGGCATCGAGAGAATCACGTGTTGTGATGATGAATGGTTTGGCATTGATAGACATGTTTATGAACT

CGAACAAATGGGCGGAGCTCTTTCCTGCAATTGTTTCAGCTGCAAAAACAATCGAAGTGATATCGCCGGGACTGTTAACT

AGTCAAAATGGCTCTTTGCAACTGATGTATGAAGAATTGCAAGTTCTTTCACCCCTTATACCAACTCGAGAGTTCTACTT

CCTCCGATACTGCCAACAGATCGAGCCAGGCTTGTGGGCAATCGTGGATGTTTCTTTCGACCTTTCAAGATATGACCAAT

TTGCTTTTCAAAGTAGATCTCAGAGACTCCCTTCCGGTTGCTTGATACAAGACCTTCCTAATGGGTATTCCAAGGTCACT

TGGGTGGAACATGTGGAAATAGAAGACAAGACCCCGGTTCATCGGCTCTACAGAGACCTCATTTACAGTGGCTTAGCATT

TGGAGCTGAACGATGGATTGCCACTTTGGAGAGAATGTGTGAGAGGATTGCCTGTCTAATGGTGACGGGTAGCTCGACTC

GTGATACCAGAGGAGTAATTCCGTCTCCCGATGGAAAGAGGAGCATGATGAAACTAGCGCAGAGGATGGTGAACAATTTC

TGCAGCAGCATTAGCACTTCCTCTAGCAGGCAACAGCGTTGGACGACACTCTCTGGCTCGAGCGAGGTTGGGGTCCGGGT

GGCTCTTCATAAGAGCACAGATCCGGGCCAACCCAACGGTGTTGTTCTTAGTGCAGCCACTACCATTTGGCTCCCGCTCT

CCCCACAAAACGTCTTTAATTTCTTCAAAGACGAAAGAACTCGAGCTCAGTGGGATGTTCTCTCCAATGGTAATGCAGTT

CAGGAGGTTGCGCACCTAGCAAATGGATCACATCCTGGAAATTGCATATCTGTTCTAAGGGCCTTCAACACGAGTCAAAA

CAACATGCTGATACTCCAAGAAAGCTGCATTGACTCATCGGGGTCTCTCATCGTGTACTGCCCGGTCGACCTGCCGGCAA

TCAACCTTGCAATGAGCGGGGAGGACCCCTCCTACATCCCCCTGCTCCCGTCGGGGTTCACCATCTCCCCCGATGGAGGG

CCTGACCCTGGGGACGGGGCGTCGTCCAGCTCTGCCGCGGCAGCTGGGCAGGGGTCAACCAGCCGCTCGGCAGGGTCGCT

GATGACTGTGGCGTTCCAGATACTGGTGAGCAGCCTGCCGTCGGCGAAGCTGAACCTCGAGTCGGTGACCACTGTAAACA

ATCTTATCTCTACGACTGTGCACCAAATAAAGGCTGCCCTCAACTGTCCTGGCTCTTGA

>EgHD-Zip2

ATGGAGAAGAGTGAGAATTGCACGGCCGGGAACATGAAGAAGAAGAAGGGCAAGAGCAAGAACATGAAGAAGGGGTTCAG

CGACGAGCAGATCAGGCTGCTGGAGACCATGTTCGAGTCCGAGGCAAAGCCAGACCCCAGGAGGAAGATGGAGCTCGCGA

GGGAGCTGGGGTTGGAGCCGCGGCAGGTGGCGATATGGTTCCAGAACAGGAGGGCTCGGTGGAAGTCGAAGCAGGTCGAG

CAAGAGTACAGGGTGCTGAGAGCCAGTTACGAGAAGCTGTTGGCCGAGTTCGAGTGTTTGAAGACAGAAAAACAGGATTT

GGAGAACGAGTTGCAGAAGCTGAGTGGCATGTTAGACAAAAACCATGGTGGGGGCAGGATCTGCAGAGAGGCTAGCAATT

TAGAGGATGAAGATACAGATTGCAAGAACGAAGCCAACCCGTTTGGGCCACACGAATCTTTCGATCAGAGTTTGATTGTC

GCCTCACCCGACGACCAATTAAACGAGACAGAGAAGTCGTCAGAAGATGTGCGGGAAAGCCGGGACGAAGAAAATTGTTC

GGTGGCATTGCCAGAGAAGTGGAGCAACTTTGGTCCGAGCGACATTTTAGACTATTCTTGCAGCAATGCGGCGCCGTGGT

GGAACTTCTGGACCTGA

>EgHD-Zip3

ATGGAGGGGCAGAGTGAGATCGACCGCTTCGGCGAGCCATTCGAGGGAAGCTTCCTGGGGAGGCTGAAGGACGAAGGCTC

CGGGAGCGACAACCAGCTCGATGGAGCTTCTGGGGATGACGATCAGGATGATGCTGTTGATGAAGAAGCAGGAGTTCTTC

CCCGCAAGAGGAAGAAGTACCACCGCCATAGCCCACATCAAATTCAAGAACTTGAAGCTTTCTTTAAGGAGTGTCCTCAT

CCTGATGAGAAACAAAGGTTGGAGCTGAGCAGGAGGCTTGGTTTGGAGTCCAAGCAAATCAAGTTCTGGTTTCAGAACCG

ACGGACCCAGATGAAGACTCAAATGGAGCGCCATGAGAATATTATGCTGAGAGACGATAACAATAGGCTCCGGAACGAGA

ACGACGCGATGCGGAGCAAACTGGCCAATCCCATTTGCGGCAACTGCGGCGGCACGGCCATCTTTAACGGCGCCGGCATG

GTGTCGTACGAGGAGCACCAGCTGAGGGTCGAGAATGCTCGGTTGAAGGAGGAAGTAGCTCGGGTTCGCGCTCTTACCGA

CAAGTTCTTGGGGAAACAACCCCCCACTGCACTCCTTCCCTCGAGCCCTTCAAGGTCCAATCGTGGTCCGGAGTTCTCCG

CCCAGGGGCACGTTCCCAACGTTTCAACCACAGCCACTGCCGCATTGCCAATGGGAATAAGCTCTTTAGATGGAATGTCG

AATGCTTCCCCAGTTAGTCCTTATGCTAGACCAGCAGTTGCGATGGGTAGGAATCAATTGTCCACTGAAAAATCGGCTTA

CGTTGAAGTTGCAATAGCTTCCATGAATGAATTGATCAAGATGGCTGAACCCAATTCCTGCCTTTGGCTGCGAACTTTGG

ACGGAGCAAAAGAAGTATTGAACCACGAGGAGTACTCACGGTTATTCACTCCTCTTGCCAGTGCGAGGCACAGTGAAGTG

GTGACCGAGGCATCAAGGGAGACCAGTATTGTCCCCATCAACAGCTTGGCCGTTATAGAGATTTTGATGGACGTGAATCG

TTGGGTGGAAACATTTCCCTGCATAGTTGCTCGATCATCCACCATCGAAGTAATGTCGAGTGGAATCAGAGGAAACAGGA

ATGGAGCTCTGCAAGTGATGCATGCTGAATTTCAAGTGCTTTCACCGTTGGTTCCTTTGCGCCAAGTGAGATTTCTTAGG

TTCTGTAAGCAGCATGGAGAGGGTATGTGGGCGGTGGTCGATGTTTCCGTTGGCATGCTCCAAGACAGTTCGGATCCGTG

TGGTTCCGCCAACTGTAGGAGGCTTCCTTCTGGATGTGTTTTACAGGATATGCCGAATGGCTGTTCCAAGGTAACTTGGA

TCGAACATTCAGAATATGATGGAACTGCAGTTCACCCTCTTTACCATTCCTTCCTTATTTCCGGCATGGGATTCAGTGCT

CAGAGGTGGATTGCCTCGCTACAGAGGCACTGTGAGTGCTTGCTAACATTCATGTCATCTACCTACCCTAATGAAGATGG

TTCAGGAATGAATCCCAGTGGAAGGAGAAATATGCTGAAACTGGCACAACGTATGACAGATAATTTCTGTTCGGGGGTCT

GTGCTTCACCGCTGCACAACTGGGAAGTCCTTCACCTCGGGAACATGAATGGCGATGTGAGGGTAACGACTAGAAAAAAC

GCTGGTAGACCCGGCGAGCCGCCCGGGATTGTACTGAGTGCAGCTACTTCCATTTGGATGCCCATAGCGCACGAGACAGT

ATTCAACTTTGTGCGCAACGAGCAGTTGAGGAGCGAGTGGGATATATTATCCCACGGTGGCCCGATGCAAGAGATAGTCC

ATGTGGCTAAGGGTCCGAGGCGTGACAATTGCGTCTCTCTTCTCCGTGCTAATGCACTCAGCGCGAGCGACACAAGCATG

GTGATTCTGCAAGAGACGTGGACAGATGCATCCGGCTCCCTCATAGCCTATGCTCCAGTGGACATAACAGTGATTGAATC

AGTGATGAGTGGCGGAGACCCGAACAGCGTCGCTCTCCTACCGTCTGGATTCGCGTTGCTCCCAGACAGTCCGTCGCACA

TTGATGGACTAAAGAATCCCGATGAAAGTCTCATAGGAACAGCTGATAGCAGGGGGTGCCTAATGACAATCGGGTTTCAG

ATCCTTGTGAGCAGCACTCCTGCTGCGAAGCTCACAGCCGAGTCAGTCGAGACTGTGCACAACCTCCTTTCGCGCACAAT

CCAGAGGATCAAAACCTCATTGCAAATGTCCTAG

>EgHD-Zip4

ATGGGAGTCGTCGACATGTCGAATAACAATCCTCCCACCTCTCGCACCAAGGACTTCTTCGCCTCTCCCGCTCTCTCCCT

CAGCCTCGCCGGGATTTTTCGGGATGCCGGGGCGGCGGCGGCGGCCTCGGCCAGCATGGACGTGGAGGAGGGGGACGAGG

GGAGCGGCGGAGGAGGAGGGAGGAGGGAGGATACGGTCGAGATCAGCAGCGAGAACTCGGGCCCCGCAAGGTCGAGATCG

GATGACGAGTTCGATCCGGACGGGGACAACGACGAGGACGGCGGCGACGGCGACAAGAGCAAGAAGAAGAAGCGGAAGAA

GTACCACCGCCACACGGCCGAGCAGATCCGCGAAATGGAAGCGTTGTTCAAGGAGTCGCCCCATCCCGATGAGAAGCAAA

GGCAGCAACTGAGCAAACAGCTGGGACTCGCTCCTAGGCAAGTGAAGTTCTGGTTCCAGAATCGCAGAACGCAGCTCAAG

GCAATTCAGGAGCGCCATGAGAATTCTCTGTTGAAAACAGAAATGGAGAAGCTCAGAGATGAAAACAAAGCCATGAGAGA

CACCATACAGAAATCTTGCTGCCCCAATTGTGGCTCAGCCACCACAAGCAGAGATACCGCCTTGACAACTCAGGAGCAGC

AACTCCGAATTGAAAATGCTCGACTGAAAGCCGAGGTCGAGAAGCTCCGAACAGCTCTAGGAAAGTACACTCCAGGGACG

GCATCGCCTTCTTGCTCAGCCGGGAACGACCAAGAGAACAGGAGCTCCTTGGATTTCTACACTGGAATCTTTGGGCTCGA

CAAGTCGAAGATCATGGAATTGGTGAACCAAGCGATGGAAGAGCTCAAGAAGATGGCTACTGCTGGAGAGCCACTTTGGA

TCAGGAGCGTTGAGACCGGCCGTGAGATACTTAACTATGACGAGTACGTGAAGGAGTTCAAGGTCGAAGCCCCTAGTGAG

GGCCGGCCCAAGAGATCTATCGAAGCCTCGAGAGAGACTGGGGTCGTGTTCGTCGACCTCCCCCGCCTCGTGCAAAGTTT

CATGGACGTGAATCAATGGAAGGAAATGTTTCCTTGCATGATCTCAAAGGCAGCAACTGTGGATGTGGTTTGCAGCGGTG

AAGGTCCCAACAGAAACGGCGCCGTGCAATTGATGTTCGCAGAGCTGCAAATGCTAACGCCAATGGTGCCCACGAGGGAG

GTGTACTTCATCAGATACTGCAAGCAGCTCAGCGCAGAGCAGTGGGCCTTAGTTGATGTCTCCATCGAAAAAGTCGAAGA

TAACATCGACGCCTCTCTTGTGAAATGCAGGAAACGCCCCTCTGGCTGCATCATCGAGGATAAATCCAACGGCCATTGTA

AGGTAATCTGGGTAGAACACTTGGAGTGCCAGAAAACCACCGTTCATCCCATGTACCGTACCATTGTTAACAGCGGCCTA

GCCTTTGGCGCAAGACACTGGATGACGACACTGCAAGTCCAGTGCGAGCGGCTCGTTTTCTTTATGGCGACTAATGTTCC

AACCAAAGATTCGAATGGTGTCGCCACACTTGCAGGAAGGAAGAGCATTTTGAGGCTGGCACAGAGGCTGACACAAAGCT

TTTGTCAGGCCATTGGAGCATCGAGCTATCACAGCTGGACCAAGGTCCCAACTAAAACTGGAGAGGACATTAGGGTGGCT

TCTAGGAAAAATCTGAATGATCCTGGAGAGCCTCTTGGGGTGATCCTATGCGCAGTTTCCTCTGTACTATTGCCAGTCTC

ACCCCACGTACTGTTCGACTTCCTCAGAGACGAATCTCGCCGTAGTGAGTGGGATATCATGGCGAGTGGAAGCCCGGTAC

AGTCCATTGCTAACTTAGCAAAAGGGCAAGATCGCGGCAATGCAGTCAACATCCAAACAATGAAAAATAAAGACAACAGC

ATGTGGGTACTCCAGGATTGCTGTACAAATGCTTACGAATCTATGGTGGTCTATGCTCCAGTGGATATCACAGGAATGCA

GGCGGTGATGACCGGCTGTGACTCTAGCAACATAGCCGCACTGCCATCTGGTTTCTCGATCCTGCCCGATGGGATCGAGT

CGAGGCCTCTAGTCATCAGCTCAAGGCACGAGGAGAAGAGCTCAGAAGGAGGATCACTGCTCACAATAGCTTTTCAAATC

CTAACAAATACCTCTCCCACAGCCAAGTTAACTGTGGAATCTGTGGAGTCTGTCAACACTCTCATATCCTGTACATTGCG

GAATATTAGAACGAGCTTGCAATGTGAGGATGGATGA

>EgHD-Zip5

ATGAGTTTCGGGGGATTCCTCGACACCAACTCGGGCGGCGGGGGCGGCGGCGGAGGAGGGGCGAGGGTCGTTGCCGCCGA

TATCCCGTACGCGGGAAAGAACGACATGGCGCCGTCGGGCGCAATCGGGCAGCCTCGACTCCTGAGCCCTTCTCTGTCCA

AGTCCATGTTCAACAACTCTCCTGGACTCTCCCTTGCTCTCCAAACCGGGATAGAGAACCAGCAAATGGGGGAGAGCTAC

GAACAAATGAGCGTGAGCAACCACCACCTTCGGAGGAGCAGAGAGGAGGAGCACGAGAGCAGATCCGGCAGCGACAACCT

CGACGGGGGCGGGGCCTCCGGCGACGACCAGGACGCCTCCGCCGACAACCCTCCCAGGAAGAAGCGTTACCACCGACACA

CTCCCCAGCAAATCCAAGAACTTGAAGCGTTGTTCAAGGAGTGCCCTCATCCTGATGAGAAGCAAAGGTTGGAGCTGAGC

AAGAGGCTTTGCTTGGAGACCAGGCAAGTCAAGTTCTGGTTCCAGAATCGGCGCACCCAGATGAAGACACAACTGGAGCG

ACATGAGAATTCGCTGCTCCGGCAAGAGAACGACAAGCTCCGGGCTGAGAACATGTCCCTCCGAGACGCCATGAGGGAGC

CGATTTGCAGCAACTGCGGCGGCCCGGCCGTCATCGGCGAGATCTCCCTCGAGGAGCAGCACCTCCGGATCGAGAACGCC

CGGCTCAAGGACGAGCTGGACCGCGTGTGCGCGCTCGCCGGGAAGTTCCTCGGCCGCCCCGTCCCGTCGCTCGCCGCTTC

CATCGGCCCGCTGATGCCCAACTCGAGCCTCGAGCTAGGGGTCGGGACCAACAACGGGTTCGTGGGTAGCTTGGGCCCGG

TGGCCGCGACGACGCTGCCGCTGGGGCCTGATTTCGGGGGCGGGATCTCGGGCAACTCGGCGATGGCGGTGGTGAACCCG

TCGAGGCCCCCGGGAGCTGGCGGGGGCATGGTATCTATCGAGAGGTCGATTTTGCTGGAGCTCGCTTTGGCCGCCATGGA

TGAGCTGGTCAAGATGGCTCAGACCAACGAGCCGCTCTGGATCAGGAGCTTGGAAGGTGGGAGGGAAATGCTGAACCGCG

ACGAGTACATGAGGACCTTCAGTCCTTGCATTGGCATGAAACCCAATGGATTCGTGACCGAGGCCTCTAGAGAGACCGGC

ATGGTGATCATCAATAGCTTGGCACTCGTGGAGACTCTCATGGACTCTAACCGGTGGGCTGAGATGTTTCCGTGTATGAT

AGCTAGAACATCGACCGCAGATGTGATCTGCAGTGGAATGGGAGGAACCAGAAATGGTGCACTTCAGCTGATGCAGGCGG

AGCTACAAGTGTTGTCGCCGCTGGTTCCAGTCCGGGAGGTGACCTTCCTCCGGTTCTGCAAGCAGCACGCCGAGGGCGTC

TGGGCGGTGGTCGACGTGTCGGTCGACGGAATCCGGGAAACTCCAGGCGGCGGTGGCGGCGCGGCCTCGTTCCCGAGCTG

CTGCCGGAGGCTGCCTTCCGGCTGCGTGGTCCAAGACATGCCCAACGGGTACTCCAAGGTGACGTGGGTGGAGCATGCAG

AGTACGATGAGAACCACATACACCAGCTGTACAGGCCGTTGATCAGCTCGGGCATGGGCTTCGGCTCTCAACGGTGGGTC

GCCACCCTCCAACGGCAGTGCCAGTGCCTCGCCATTCTCATGTCCTCCACCGTCTCTTCTCGTGATCACACCGCGATAAC

GCCGAGCGGGAGGCGCAGCATGCTGAAGCTGGCGCAGCGGATGACGGCCAACTTCTGCGCGGGGGTGTGCGCCTCCACGG

TGCACAAGTGGAACAAGCTGAGCGGCGGGAACGTGGACGAGGACGTCCGGGTGATGACCCGGAAGAGCGTGGACGACCCG

GGGGAACCGCCGGGCATCGTGCTGAGCGCGGCTACCTCCGTGTGGCTCCCCGTCTCCCCACAGCGCCTCTTTGACTTCCT

CCGCGATGAGCAGCTCCGGAGCGAGTGGGATATCCTCTCCAATGGCGGCCCCATGCAGGAGATGGCCCACATCGCCAAGG

GCCAAGACCACGGCAACTGCGTCTCCCTCCTACGTGCCAGCGCCATGAACGCGAATCAGAGCAGCATGCTGATACTGCAG

GAGACGTGCATCGACGCTGCCGGGTCGCTCGTGGTGTACGCGCCCGTGGACATCCCCGCCATGCACGTGGTGATGAACGG

CGGGGACTCCGCCTACGTGGCGCTCCTCCCGTCGGGGTTCGCCATCGTCCCCGACGGGCCGGACGGGCCGGGCTCCCACG

GGGGCGGGGCTGCCCCCAATGGGCCCGCTGCGAGCAACGGGGGACCGCCAAGGGTGGGCGGGTCGCTCCTGACGGTGGCG

TTCCAGATCCTGGTGAACAGCCTGCCCACGGCGAAGCTCACGGTGGAGTCGGTGGAGACGGTCAACAACCTGATCTCGTG

CACGATCCAGAAGATCAAGGCCGCGCTCCAGTGCGAGAGCTGA

>EgHD-Zip6

ATGCCAGCTGGAATCATGATTCCGGCAAGGAACATGTCGTCATCGCCGATGATCGGATCCAATGGAAATGCCAGTGGGTA

TGGATCGTCTTCCGGGCTCACCCTCGGTCAGCCAAACATGATGGAAAGCCTTCATCATCATCAGCTTCACCACCCCTTAG

ACATGGCTCATCAGACCCCGTCAGAGAGCGAACTTGCTCGGCTCCGAGATGAAGAGTTCGAGATTAGCACAAAGTCGGGG

AGCGAAAACCAGGAAGGTCAATCTGGAGACGACATGCAGGATCTTCAGCGTCCCAATAAGAAGAAGCGATACCATCGTCA

CACCCAGCATCAGATCCAAGAGATGGAAGCTTTTTTTAAGGAATGTCCGCATCCAGATGACAAGCAGAGGAAGGAACTCA

GCCGTCAATTAGGCTTAGAGCCTTTGCAAGTCAAGTTTTGGTTCCAAAACAAGCGCACTCAAATGAAGACACAGCACGAG

CGCGGCGAGAACACGCAGCTCCGAGCGGAGAATGACAAGCTTAGGGCCGACAATATGAGGTACAGGGAGGCACTAAGTAA

TGCCACATGTCCCAACTGTGGAGGGCCGACAGCCATCGGAGAGATGTCATTCGATGAACACCACTTGAGATTGGAAAATG

CACGTCTAAGAGAAGAGATCGATAGAATTTCAGCTATCGCTGCCAAGTATGTGGGCAAGCCAGTGGTGAACTATCCTCTT

CTTTCCCCTTCGGCTCCACCTCGTCCACTTGACCTCGGAGTCGGAGGTTTTGGGGGTCAATCTAGCGTAGGAGGAGAAAT

TTATGGAGCTGGAGACCTTCTCAAGTCAATTGGCGCCCCGACAGAAGCCGACAAGCCAATGATCATTGAGCTGGCGGTCG

CAGCCATGGAGGAATTGATCAGGATGGCCCAGATGGGAGAGCCCCTCTGGAGTGAGAGCTTGGATGGCAATTCATCAAGT

GTGCTAAATGAAGATGAGTACATGAAAACATTTCCTCGGGGTATCGGGCCAAAGCCTGCTGGATTTAACACCGAAGCTTC

ACGAGAAAGTGCTGTTGTCATTATGGATCACATCAACCTTGTTGAGATTCTCATGGATGTGAATCAATGGTCGACAGTTT

TCTCCAGCATTGTCTCACGAGCTGCAACATTGGAAGTACTATCAACAGGAGTAGCTGGCAACTACAATGGAGCTTTGCAA

GTGATGACAGCTGAATTTCAAGTTCCTACACCACTTGTTCCAACTCGCGAAGTCTACTTTGTTCGGTACTGTAAACAGCA

CGGTGATGGGGCTTGGGCTGTTGTTGATGTTTCCTTGGACAATGTACGCCCAAGCCCGATTGTCAGGTGCCGAAGAAGGC

CTTCAGGATGTGTAATTCAAGAAATGCCAAATGGATATTCCAAGGTTACATGGGTTGAGAATGTGGAAGTGGATGATAGA

GGAGTCCACAGCTTATACAAGCAGCTTGTTAATTCTGGTCACGCCCTTGGGGCTAAACGGTGGGTCGCCACCTTAGATAG

ACAATGTGAACGCCTTGCCAGTGCCATGGCAACAAATATTCCTAATACCGATGTCGGAGTGATAACGAGTCAAGAAGGAA

GAAAGAGCATGCTAAAACTAGCCGAGAGAATGGTGATAAGCTTCTGCGCCGGAGTCAGCGCATCTACAGCTCACACATGG

ACGACATTATCAGGGACAGGTGCCGACGATGTGAGGGTCATGACCAGGAAAAGTATAGACGATCCGGGTAGACCCCCCGG

TATAGTACTGAGTGCTGCGACTTCTTTCTGGCTTCCGGTGCAGCCAAATAGAGTCTTCGATTTCCTCCGTGATGAGAATT

CTCGAAGCGAGTGGGATATTCTCTCGAACGGAGGGGTGGTTCAAGAAATGGCGCATATCGCAAATGGCAGGGACACTGGC

AACTGTGTATCTCTACTACGAGTAAATAGTGCTAACTCAAGCCAAAGCAACATGCTGATCTTGCAAGAGAGTTGCACCGA

CTCTGCCGGTTCTTTCGTAATTTACGCTCCAGTGGATATCGTCGCTATGAATGTGGTACTGAACGGTGGGGATCCAGATT

ATGTCGCGCTTCTTCCTTCAGGGTTTGCAATACTGCCTGATGGTTCGGCCATGCAAGGTGGCAATGATCAACAGGGAGTG

GGATCTGGTGGTGGGTCTCTCCTCACAGTGGCATTCCAGATTCTGGTTGATTCAGTTCCTACTGCTAAGCTCTCTCTTGG

GTCAGTTGCGACTGTTAACAATTTGATCGCTTGCACTGTCGAGAGGATCCGGGCTTCATTATCGTGTGAAAGTGCATAA

>EgHD-Zip7

ATGGAGGCAGGGCGTTTTCTGTTTGATCCCCCCGCGCTTCAGGGGAACATCCTCTTCCTTGATAAAGGATCAAGATCCAT

GATGGGCATGGAGGAATCCCCGAAGAGGCGCCGGTTTTTCTGCTCGCCGGACGAACTTTTCGATGAGGAATATTACGATG

AGCAGATGCCAGAAAAGAAACGTCGCCTCACTCCTGAGCAGGTGCTTCTGCTGGAGAAGAGCTTTGAGGAAGAGAACAAA

CTGGAACCGGAGCGGAAGACCCAGCTGGCTAAGAAGCTGGGGTTGCAGCCAAGGCAGGTTGCTGTGTGGTTCCAGAATCG

CCGGGCTCGGTGGAAGACAAAGCAACTGGAAAGGGATTATGATTACCTCAAATCTTCATACGATTCCCTTCTTTCGGACT

ATGATTCCATCTTGAAGGAAAACGAGAAGCTCAAACTGGAGGTCTATTCCTTGACAGAAAAACTTCAGGGCAAGGAAGTC

GATGGAGCACCAATGACAGGCCCCTCGGAGCCAGCTCCGCTGGAGGAGGCTGATGTCCAGGCCGTCCAATTCAGTGCGAA

GGTGGAGGATAGGCTGAGCACAAGGAGCGGGGGAAGCGCAGTGATCGACGAGGAAGGTCCACAGCTTGTGGACAGTGGCA

ACTCGTACCTCCTGTGCGAAAATTACCCCGGATGCGTAGCCCAATCGGAGGATGATGGAAGTGATGACGGCCGGAGTTAC

TTCCCGGGCGTTTTTGCCGCCGCGACTGAGCAGCCACACCACGAAGAAGAGGAGGAGCCAATGGACTGGTGGGTGTGGTC

TTGA

>EgHD-Zip8

ATGTTTGGAGATTGCCAGGTGATGTCATCCATGGGAGGGACTGGTATTTCCGCCGAGTCCCTCTTCTCGTCGCCCATCCG

AAACCCTAACTTTGACTTCATGTCGAGCTTGCCCTTCAACGCCTTCGCTCCGCTCATTCCGAAAGAAGAAGAGATTAATG

GAATGCTGGGAGGGAGCAGCAACAGCAAGGAGGAGATGGTGGACAGTGGAGGGTCGGGGAGTGAGCATTTCTTGGAAGAG

AAGTCGTCGGCGAACGAGCTATTAGATCTAGACGGCGGCGATCAAGATCAGCCTGCTAACAAGAAGAAGCGTTACCATCG

ACACACCGCTCGTCAGATCCAAGAAATGGAAGCATTGTTTAAGGAATGTCCTCATCCGGACGACAAGCAGAGGATGAGGC

TGAGCCAGGAGCTAGGGCTCAAGCCTCGCCAGGTCAAGTTCTGGTTCCAAAACCGGCGAACCCAAATGAAGGCACAACAA

GACCGAGCTGATAATGCGATACTGAGGGCCGAGAACGATAATCTGAAGAATGAGAATTACAGGCTTCAAGCAGCCTTGCG

CAACATAGTGTGTCCTAATTGTGGAGGCCCAGGGATGCTGGGAGAGATGGGCTTTGATGAGCAGCAGCTTCGCATCGAAA

ATGCTCGACTAAAGGAGGAGTTGGAACGTGTGTGCTGTCTAGCTTCACGGTACAGCGGCCAACAAATACAATCAATTCCA

GTGCTGCCGCCGCTGTCTCTTTTACCACCATCGCTTGACTTGGAGATGACCATCTACCCACCAAGGCATTTCCCGGAAGC

CATGTCCTCTTGCTCAGACATCATGCCCATGCCTTTGTTGCCGCAAGACACTCCACACCACTTGTCCGAAAGCACTGGCC

TCATACTCATGGAAGATGAAAAAGCACTTGCCCTAGAGCTCGCCATGTCCTCCATGGAGGAACTCTCAAAAATGTGCCTC

GCGTCCGAGCCCCTTTGGGTCCAGAGCAGTGAGAGCGGAAAGGTAGTGCTCAATTTCGAGGAGCATGCGCGGTTGTTCCC

ATGGCCATTGAACAATAATCTCAAGCAAAACTCAGACAAGCTTAGGAAAGAAGCTAGTAGAGACAGTGCTGTTGTTATAA

TGAATAGCATTACTCTGGTTGATGCCTTCATGGATGCTGGCAAGTGGATGGACTTGTTTCCCACCATTGTTTCTAGGGCA

AAAAATATTCAAGTCATAGCATCAGGCGTCGCAGGTCATGCAAGAGGATCTCTTAACCTGATGTACGCAGAATTGCAAGT

TCTTTCACCGATGGTACCGACACGAGAGACATACTTCCTTCGTTATTGCCAACAAAATGTGGATGAGGGAAGTTGGGCGA

TTGTTGATTTTCCCATGGACAGCCTTCACAACAATCTCGCTTCACCACCTTCTATGCCCAGATACATCAGACAACCTTCC

GGTTGCGTCATTCAAGACATGCCCAATGGCTACTCAAGGGTGACCTGGGTCGAGCATGCAGAGATTGAGGAGAAAGTGGG

GCACCCACTGTTTAGTCAGTTTGTGAAGTGCGGCATGGCATTTGGAGCACATCGGTGGTTGGCGGTCTTGCAAAGACAGT

GCGAGAGGGCGAGGAAGAACCTGATGAGGTTGGCTCAGAGGATGATAAGGACATTCAGCACCAACATCAGCACCTCCGGT

GGCCAATCATGGACGGCGCTATCGGACTCCTCCGACGACACCATCCGCATCACCACCAGGAAAATCACCGAGCCCGGTCA

GCCCAACGGCGTTGTCCTCGCCGCCGTATCCACAACCTGGCTTCCCTACCCTCACCACCATGTCTTCGAACTCCTGAAGG

ATGAGCGCCGCCGATCTCAGCTAGATGTGCTTTCAAATGGGAATTCTTTGCAAGAGGTGGCTCACATTGCGAATGGTTCT

CACCCTGGAAATTGCATCTCTCTCCTTCGCATCAATGTGGCGAGCAATTCGTCGCAACATGTGGAGCTAATGCTTCAGGA

GAGCTGCACCGACGCCTCCGGTAGCCTCGTCGTCTACTCCACCATCGACGTTGACTCCATCCAGCTCGCCATGAATGGCG

AGGACCCTTCCCACCTTCCTCTCCTCCCTATCGGCTTCTCCATCGTCCCCTTCGGATCGGGTTCACCCGGATCCGACTCC

GGCGCCAATGACAACCTGGGGCTCGACGCTTGCCCTCAAGGCGAAGGTTGCCTCCTGACGGTGGGGCTCCAAGTCCTGGC

CAGCACAATCCCAACAGCCAAGCTCAGCCTCTCCAGCGTGAACGCCATCAACAACCACCTCTGCATGGCGGTCCACCAAA

TCACCGCCGCCCTCGCCAGCAGCTCCAGCAACGGCAATTCTTCCCCAGCCCCCTGCAAT

>EgHD-Zip9

ATGAACGACCTGAGCGAAGATGACATGGCGCTCATCTCTGAGCTTTACCCTGGAGTGTACATGCGACCATCTCCTCCTCC

ACAAGAGGCAAAGCCGCCGCGGCGGAGGCGGAAGAAGAGCAGAGGACTCGAGAACCCGGGGGAAGAAGGAGGAGGTACAG

GAGGCGAGGGGCGGGGGAAGCGGAAGCTGACCGCGGAGCAGGTGGAGCTGCTGGAGCAGAACTTCGGGGACGAGCACAAG

CTTGAGTCGGAGCGGAAGGACCGGCTAGCGGCGGAGCTCGGGCTCGACCCGCGGCAGGTGGCGGTGTGGTTCCAGAACCG

GCGGGCCCGGTGGAAGAGCAAGAAGCTGGAGGTGGAGTACGCCAAGCTGAAGTCCGTCCACGAGACCGTCGTGGTGGAGA

AATGCCGTTTTGAGTCCGAGGTGCTGAAGCTCAAGGAGCAGCTTTCGGAGGCAGAGATGGAGATCAAGAGGCTGTCGGAG

AAAGCTGATCTCGGGCTCTCCGGCAACAGCCCATGCTCCTCCGTCACGGCCGATGCTGCTACAGACGATCCC

>EgHD-Zip10

ATGATGGACATATTCCAGCAAAACTTGATGGAGGGCGGTGGTGGTCCACCGCTTCACCCTCTAGACATGGTGCAGAATCC

GTCGGAGAGCGAGCTTGCTCGCCTCCGGGAGGACGACTTCGATAGCACCAAGTCTGGCAGCGACAACCATGAGGAGCCGC

CCTCCGGCGACGATCTGCAGGATGAGCGCCGGAAGAAGAAGCGCTACCATCGTCACACCCAGCACCAGATTCAAGAAATG

GAAGCATTTTTCAAGGAGTGTCCACACCCGGATGATAAGCAAAGGAAGGAACTGGGGCGTCAATTAGGGCTTGAGCCTTT

GCAAGTCAAGTTTTGGTTCCAAAACAAGCGCACTCAAATGAAGACCCAACATGAGCGCCACGAGAACACGCAGCTCCGCA

GCGAGAACGAGAAGCTCCGGGCTGATAACATGAGGTACCGGGAAGCGCTCACTAACGCTTCCTGTCCCAACTGTGGAGGC

CCGACTGCCATAGGAGAGATGTCCTTCGATGAACATCACTTGAGGCTTGAAAATGCACGTCTGAGAGAAGAGATTGATAG

GATATCAGCAATAGCTGCAAAATATGTAGGCAAGCCGATAGCGAACTACCCTCTTCTTCCTTCTTCGGTCCCTCCTCGCC

CACTTGAGCTTGGTTTTGGAAGTTTCTCGGGCCAGTCAGGTGTCGGGGGTGACATTTATGGCAGCGGGGACTTATTCAGA

TCAATCCCTGCACCAACTGAAGCCGAGAAGCCGATGATTATCGAGCTGGCCGTGGCGGCCATGGAGGAACTAATCCGAAT

GGCGCAGGAGGGAGAGCCCATGTGGATTGCAGGACCCGATGGCTCTTCAAGCGTGCTGAATGAAGACGAGTACATTCGCG

CATTTCCTCGTGGGATCGTAACAAACCCCACCGGATTTAAGCGCGAAGCCTCACGACAAACCGGGGTCATCATCATGAAT

CACATCAATCTTGTTGAGATTCTCATGGATGTGAACCAGTGGTCCACTATATTCTCAAGCATCGTGTCAAGAGCTATGAC

TTTGGAAGTACTATCAACTGGGGTGGCAGGGAATTACAATGGAGCCTTGCAAGTGGTGACGGCTGAATTTCAAGTTCCGT

CGCCGCTTGTTCCGACTCGGGAAGTTTATTTCGTCCGCTACTGCAAACAACATAGGGATGGGACTTGGGCAGTGGTTGAT

GTTTCTTTGGACACTTTACGCCCAAACCCAGCAGTTCGATGCCGCAGGAAGCCTTCAGGATGTCTAATTCAAGAAATGCC

GAACGGATACTCTAAGGTCACATGGCTTGAGCATGTGGAAGTAGATGATAGAGGAGTGCATAACCTATACAAGCAACTTG

TTGGCTCTGGCCATGCTTTCGGGGCCAAACGCTGGGTTGCTACGTTAGATCGACAATGTGAACGTCTCGCAAGTGCCATG

GCAACTCATATTCCTAGCGGAGATGTTGGTGTCATAACGAGTCCGGAAGGGAGAAAAAGCATGATGAAGCTGGCTGAGAG

GATGGTAATAAGCTTTTGTGCCGGAGTCAGCGCATCTACAGCTCAAACATGGACGACATTATCTGGAACCGGAGCAGACG

ACGTGCGTGTGATGACTCGGAAAAGTGTGGATGATCCGGGCAGGCCTCCCGGAATAGTTCTGTGTGCTGCCACTTCCTTC

TGGCTGCCAGTTTCCCCCAAGCAGGTCTTCGATTTCCTTCGTGACGAGAATTCTCGAAGTGAGTGGGATATTCTCTCAAA

TGGAGGGATTGTTCAAGAAATGGGTCACATCGCCAATGGCCGGGACACCGGCAACTGTGTGTCTCTGTTGCGAGTAAATA

GTGCAAATTCAAGCCAAAGCAACATGCTGATATTGCAAGAGAGCTGCACTGATGCGACAGGCTCTTTCGTGGTATATGCT

CCGGTTGATATCGTCGCGATGAATGTGGTTCTAAATGGCGGAGATCCCGATTATGTGGCACTTCTTCCCTCGGGGTTTTC

CATTCTTCCTGATGGCTCGCAAGGCAGTGGCGAAGTGGCATCCGGTGGATCTCTCTTGACTGTTGCATTTCAAATTCTGG

TCGATTCAGTCCCAACTGCTAAGCTTTCTCTGGGATCGGTCGCAACTGTTAACAATTTGATTGCTTGCACTGTTGAGAGG

ATTAGGGCTTCTTTATGGTAA

>EgHD-Zip11

ATGGGAATTGAGGAGGCCACGAAGAGGCAATCGATTTTCAGCTATCCTGAGGATCTTTACAACGAGGAATATTATGATGA

CCAGGCGCCGGAAAAGAAGCGCCGCCTCACTCCTGAGCAGGTGCATCTGTTGGAGAAGAGCTTTGAAGTAGAGAACAAAT

TGGAACCAGAGAAGAAGACCCAGCTGGCGAAGAAGCTGGGCATGCAGCCGAGGCAGGTGGCTGTGTGGTTCCAGAATCGT

CGAGCCCGGTGGAAGACAAAGCAGCTCGAGAGGGACTATGATGTCCTCAAGTCGTCTTATGACTCACTGCTTTTGAGTTA

CGACTCCATCAAGAAAGAGAATGAGAAGCTCAAATCTGAGGTCAATTTCCTGAACGAGAAGCTGTCCGCCAAAGATGTCG

CCGGAGCACCAGCCTCGGGTCAAAGTTCCCATGCACTTCAAGTGGAGGAAACCCGAGATTCACCCCTCGGGTTTGTGGTG

AAGGTGGAGGACCGCCTGAGTTCCGGGAGCGGTGGGAGCGCCGTGGTGGATGAGGACGGGCCGCAGCTCGTGGACAGCGG

CCATTCATATTTTCATTGCAATGACTACCCGGGAAGCTTGGTGGCCGTCAATGGGTTGCAGTCAGAGGACGATGGAAGCG

ATGATAGCCGAGGTTACTGCTCAGAGATTTTCGCCGCTGCTGAAGAGCCGCATCAGGAGGGAGGCGTGCCTATGGGGTGG

TGGGCGTGGCCCTAG

>EgHD-Zip12

ATGTTCCAACCCAACATGTTCGACAGCCACCATCACTTGCTTGACATAACTGGCGCGAGAAGCTCCGAGAGCGAAGTGCT

GAAGATGAGAGAAATCGAAGATTTCGAGACCAAATCTGGCACTGAAACCATGGAACCCGACCCTTCAGGCGACGACGTTC

AAGATCCCAACAACGACGACAACAGCAATAACAATGGTCAACGCCAGAAAAGAAAGCGTTACCATCGCCACACTCAGCGT

CAAATCCAAGAGCTTGAAGCTTTCTTCAAGGAGTCCCCTCACCCAGATGACAAACAAAGAAAGGAACTGAGCCGGGAGCT

AGGGTTGGAGCCTTTGCAAGTCAAGTTTTGGTTCCAAAATAAGCGAACCCAAATGAAGGCTCAACATGAACGCCACGAGA

ATGCAATTCTGAAGGCTGAAAATGAGAAGCTTCGGGCTGAGAACATGAGGTATAGGGAAGCTCTCAGCACCGCCACCTGC

CCCAACTGCGGTGGCCCTGCCGCCCTTGGGGAAATGTCTTTTGATGAGCAGCATCTGAGAATTGAAAATGCTCGTTTGAA

AGAAGAGATAGATAGGATATCCGTGATTGCTTCGAAGTATGTCGGGAAGCCGTTGGCTTCTAACTACCCTCATCTTCCTC

CTCACATGTCATCCCGCTCCCCAGATCAATTCCCGGCACAATCAGGTCTTGTTGGAGAGATGTATGGGGGAATTGATCTC

AGAAGATCAGTATCGATGCCTTCTGAAGCAGATAAGCCATTGATCGTCGAGCTTGCGGTTGCGGCAATGGAGGAACTGAT

TAGGATGGCACAAGGAGGAGAGCCATTGTGGATTCCTGCGGGCAGTGGCCAACCATCAGAGATATTGAATGAAGATGAAT

ATTTTAGGATCTTCCCTAGAGGAATTGGGCCAAAACCTTTAGGGTTCAAATCGGAAGCTTCGCGAGAGTCAGCAGTGGTT

ATTATGAATCACATTAACCTTGTCGAAATACTCATGGATGAGCATCAATGGTCAGGTGTTTTTTGTGGCATCGTCTCAAG

AGCAATGACCATCGAAGTCCTATCAACTGGTGTTGCCGGGAATTACAATGGGGCATTGCAAGTGATGACAGCTGAGTTCC

AGGTCCCATCGCCACTTGTTCCTTCTCGAGAAAATTATTTTGTGAGATATTGTAAGCAACACGGTGATGGAACTTGGGCT

GTGGTCGATGTTTCCTTGGACAATATACGAGGGAATCCCATACTACGGAGCCGGAGAAGGCCATCCGGCTGTTTGATCCA

AGAATTGCCCAATGGTTACTCAAAGGTCACATGGGTCGAACATGTAGAGGTTGATGATAGAGCTGTTCATAGCATATATA

GGCCCCTAGTGAATTGTGGCCTTGCCTTTGGAGCGAAACGTTGGGTGGCGACACTGGACCGGCAGTGTGAACGTCTCGCG

AGCTCAATGGCCATTAACATACCATCAGGAGATCTGTTGATAACAAGCCCAGAAGGAAGAAGGAGCATGTTAAAGTTGGC

TGAGAGGATGGTGCTTAGCTTCTGTAGCGGTGTTGGTGCTTCAACTGCACATGCTTGGACGACATTATCAGCGGCTGGTT

CTGATAATAATGTGAGGGTCATGACAAGAAAGAGCATGGATGAGCCGGGCAGGCCTCCTGGAATTGTGCTAAGTGCCGCA

ACTTCCTTCTGGCTTCCAGTTCCCCCCAAGCGGGTTTTTGATTTCCTCCGTGATGAGAACTCTCGCAATGAGTGGGATAT

ACTTTCAAATGGGGGACAAGTTCAAGAAATGGCACACATAGCAAATGGTCGCGACCCTGGCAACAGCGTCTCGTTACTCC

GCGTAAATAATGCAAATTCGAGCCAGAGCAACATGCTTATACTGCAAGAAAGCTGCACAGACTCTGTTGGTGCTTATGTG

ATCTATGCTCCAGTTGACATTGTCGCTATGAATGTCGTATTAAATGGTGGCGACCCCGACTATGTAGCGCTGTTACCCTC

AGGTTTTGCCATACTTCCTGATGGGCCAGAGTTTGGTGGAGGAGGAGGCATTCTCGAGATTGGCTCAGGTGGCTCGCTAC

TGACCGTCGCATTTCAAATCCTAGTTGACACAGTACCATCTGCTAAACTCTCTCTTGGGTCGGTGGCTACGGTGAACAAC

CTGATCAAGTGCACCGTTGAAAGAATCAAGGCATCAGTGTCGTGTGACAACCCATGA

>EgHD-Zip13

ATGTTTAGTACGGGAGAATATTCTGCTGCTGCCTTCGAAGGCATGGACTCGCTCCCGAGCCCAAGGAAGATGAAGAACCA

GCTGGTGAACAGAAGAAGGTTCAGTGATGAACAGATCAGGTCACTGGAGTCTATCTTTGAATCCGAGTCGAGGCTAGAGC

CTCGGAAGAAGCTGCAGCTCGCTAGGGAATTGGGGCTGCAGCCCCGCCAGGTGGCCATTTGGTTCCAGAACAAGAGAGCC

CGATGGAAGTCCAAGCAGCTGGAGCGTGAGTTCGCCATTCTTCGCGCCAACTACAACTCCCTCTATTCCCAGTTCGAGTC

TCTCAAGAAAGAGAAGCACTCCTTGGTCACTCAGATTGAGAAACTAAACCAACTCGTCGAGAAGCCGCAAGGAGAGGGCC

AGAGCTGCGGGCATGATTTGGCAACGAACAGCATCGATCGCGAATCCGACAATGGGGTTCCCAAGCATGAAGACAGTCAG

CCTGTATTTCCGGATAAACTAACGCGTTTGATGGGAATCCCATGTGAGGATGACTACTTTGGCCTAGAAGAGGAGCAGAG

CCTCCTAAACATGGCCGAACCCGATCCTGAACCTGCCCCTGTGGATAGTTCCTTGACATCACCTGAGCCGTGGGGCAGCC

TGGAGACTGATGATCTTCTCAATCAATCGTCCGGCAGTTCGCAGTGGTGGGACTTCTGGTCTTGA

>EgHD-Zip14

ATGGCGGGCGGTGCTTCGGCTTCGGCTTCGGCTATCACGACTCTGCTTCAGAACCAAAGGGTCCCTCCTCCTCCTCCTTC

TGATGCCTTTTTCCTTTCTGGGTCTTCTCCTTTTCTGGGTTCAAGAGCCATGGTGAGCTTCGAAGATGCTCACAGGGGAA

ACAGATTGAACAGACCGTTTTTCCGGTCAATCGACCCTGATGAAAACGGGGAAGACGACCTGGAGGATTACTTCCATCAG

CCTGAAAAGAAGAGGCGCCTGACTGTTGAGCAGGTACAGTTTCTTGAAAAGAGTTTTGAAGTAGAGAACAAGCTCGAGCC

AGATCGCAAAATCCAGTTGGCAAAAGACCTCGGATTGCAGCCACGACAGGTAGCGATATGGTTTCAGAATCGTCGTGCAC

GGTGGAAGACGAAGCAGCTAGAGAAGGATTATGAAACTTTGCAAGCTTCTTTTAACACCCTGAAGTCAGACTACGACACT

CTCATCAAGGAGCGGAATGATCTGAAAGCCGAGGTTCTTAACCTCACGGACAAGCTGCTTCACAAGGGAAATGAGAAGGA

GAGTTCCGAGTCGTCCAGCAAATCATCTCAAGGGCTATTCCAGAACCCCATTGCTGATTCTGTTTCTGAGGACGAAGTGT

CCAGAGTCCCCATTCCTACATGGCCAGAGGATATTTGCTCGGTCAAGAGCGACATGTTCGATTCTGAAAGTCCGCATTAC

ACTGACGCTGCCCACTCTTCGCTCTTAGAGCCCGGCGATTCTTCCTATGCTTTCGAACCTGACCATTCGGACCTATCTCA

AGATGAAGAAGATAATTTGAGCAAGAGCCTTTTGTCCACTCGCAATTACCCAAAGCTCGAAAACTCTGACTACGCCATCC

TGCCTCCAAATTCGTGTAACTTTGGATTCCATGCTGAGGATCCTGCCTTTTGGCCTTGGTCATACTGA

>EgHD-Zip15

ATGGACTGGCATGGCGACTTGAGGAGCTTCAGTAACGTCTCTTTCCCTGAATCTTCCATCGGCTTTCTCAACCCCAGCTT

CGCCCAATACCCTGGTATGGAGCTGAAGCACCACCACCACCACCACCCCCCGCCGCTGTCCTCCTCGATGGCTGAGGCCC

ACAACGACGTGGTGGTTGCAGCGTCGATGGACAAGAAGAAGAAGCTGACGAGCGAGCAGCTGGAGATGCTGGAGAGGAGC

TTCCAGGAGGAGATAAAGCTGGACCCGGACAGGAAGATGCGGCTGTCGCGCGAGCTTGGGCTCCAGCCCCGCCAGATCAC

CATCTGGTTCCAGAACCGCCGGGCCCGGTGGAAGACGAAGCAGCTGGAGCGCCTCTACGACGCCCTCAAGCTGGAGTTTG

ACGCCGTCTCGAGGGAGAAGCTCAGGCTTCAAGAGGAGGTCTCAAGATTGAAAGCCATGGTGAGCGACATCCAAGCGGCC

AAGAAGAAGCAAGTCTCCACCGGCTACACTGACATCTCCGGTGACGAGACGGTGGAGAGCGCTCGCTACTCCGGTGACAT

CCGCCACTGTAGCGACCTCACTCGGGGAGCAGGAGGAGGAGGAGGAGTGACGCCGACGCCCTCGACCACTCAGCACCACC

CGCTCGCAGGCTCGAGCTATCAACTGTTCACCCTCCAAGACTACGAGTCCAACTCCTACTGGGGCGTCCTGCCCTCATCA

TACCCATGA

>EgHD-Zip16

ATGGGAGATTTGGGAGATTTCCCCGGGAGCTCCTCGAGCCTGGAGTTGACTATTTCCGTCCCGGGCTTCTCTTCCTCCCC

TCCCCTCCCTTCGTCTGTGAGGGACCTGGACATAAACCAAGTGCCGTCGGGGGGCGAGGAGGAGTGGATGACGATGAGCG

GAGGGATGGAGGACGAGGAAGAGAGCAGCCACGGCGGCCCTCCTCGCAAGAAGCTCCGCCTCACCAAGGAGCAGTCCCGC

CTCCTCGAAGAGAGCTTCCGACAAAACCACACGCTCAACCCGAAGCAAAAGGAGGCACTGGCGGCGCAACTGAAGCTCCG

CCCGCGCCAGGTCGAAGTGTGGTTCCAAAACCGTAGGGCCAGGAGCAAGTTGAAGCAAACGGAGATGGAGTGCGAGTACC

TGAAGAGGTGGTTCGGATCGCTGACGGAGCAGAACCGGCGGCTCCAGAGGGAGGTGGAGGAGCTTCGTGCCCTCAAGGTT

GGCCCGCCCACCGTCATCTCCCCCCACACATGCCAGCCCCTCCCCGCCTCTACCCTCAGCATGTGCCCTCGCTGCGAGCG

CGTCACCTCCACCGCCCCTCCCCCCACCAGCACCTCCGCCCCTTCCGCCGCGACCAGGGCGCCGTCTAAAGCATCACAAC

TGTCGCCCCCTTCAGTTCATTCGAGGCAGCCATCAGCGGCTTCCTAG

>EgHD-Zip17

ACAAACTCCAAAACAAGGCCATTCTTTGGTGGAGTCGGCGTCAACCGAGACATACTGGTGGTGGATTTGGATGATGATAA

TCATGTCTCATCACCTAACAGCACCGTGTCGAGCATAAGTGGGAAGAGAAGTGAGAGAGATCCGCATGGAGAAAATGAAG

TTGAGGCTGAGAGAACTTCGTGCTCTTGTGAGAGCATCGAAGAAGATGGCAGCGGAGGAGATGGAGCGAGGAAGAAGCTC

AGGCTGTCTGGAGAACAGTCATTGGTGCTAGAGGAGACATTCAAAGAGCATAACACTCTTAACCCTAAGCAAAAGCTTGC

CTTGGCCAAGCAGCTGAATCTTAGTCCCAGACAGGTAGAGGTCTGGTTTCAGAACAGGCGTGCCAGGTCTAAGATGAAGC

AAACTAAGGTGGACTGTGAGTACCTTAAGAGATGCTGCGACAATCTTACACAGGAAAACAAGAGGCTGCAGAAGGAAGTG

CAGGAGCTCAGAGCACTGAAGCTCTCCCCACAGCTCTACATGCATATGAACCCTCCCACCACGCTCACCATGTGCCCTTC

ATGTCAGCGAGTGTCTGTTCCTTCAGCATCATTGTCGCCTCCATCTTCATCTTCAGTCACTGCCTCAGTGATTGGCCCCA

TAGGGCCGGTCCATAATCCTTTTAGTCAGTCTGGACCATCCATCAACTCTTGGGCAAGGCTACAGATGCAACAAGGATTG

AACAATTTGCACTCTTAA

>EgHD-Zip18

ATGGCGGGCGGTGGTGGTTGCGAGGGTTCGGCGATCGCATCTTTGCTTCAGAACCAGCGAGTCTCTCCCTCTTCCGATGC

CTTTTTCTTTTACGGGTCTTCTTTTTCTGTAGGATCGAGATCCATGGTGAGCTTTGAGGATGCTAGCGGGGCAAACGTGT

CGAAAAATCCCTTCTTCCAAGCGTTTGATCCCCATGAAATCGGGGAGGAAGAGCTGGACGAGTATCTCCATCAACCCGAG

AAGAAGAGGAGGCTGACGACCGAGCAGGTCCATTTCCTCGAGAAGAACTTCGAGCTGGAGAACAAGCTCGAGCCGGAGAG

GAAGATCCAGCTCGCCAAGGACCTCGGTTTGCAGCCCCGGCAAGTCGCGATATGGTTCCAGAACCGCCGGGCCCGATGGA

AGACCAAGCACTTGGAGAAGGAATACGAAGATCTGCAAGCCAGCTATAACAGCCTCAAGGCCGACTGCGACGGCCTTCTC

AAGGAAAACGATAAGCTCAAAACCGAGGTTCTTGTCCTTACGGACAAGCTTCTTATCAAAGCAAGAGGAACGCAAAATTC

CAAATTGTCCAACGCATCATCTCCTGGACCACCGGAGAATCCCGTCGCCTGCTCCAAGGGCGAAGAAGAGAGAATTTCGA

CGGTACCCGAAGATGTCTGTCCCGGCAAGAGCGAGATATCGGATTCCGACAGTCCAAATGGTGGCTATTCTCCGCTGAGA

GAGCACGGTGATTCTTCCTACGCCTTCGAGCCCGACCTCTCGGATTCGTCCCAAGACGAGGACTACACGCGGAATGAGAA

CCTGCAGCATCTGTGCGTCTTCCCGAAGCTAGAAGAGACTCGAGACGATTACCCCATCCTATCCACAAGCTCGTGCCATT

TCGGATTCCCTCCCGAAGATCAAGCCTCCTGGCCCTGGCCGTACTAA

>EgHD-Zip19

ATGGCAACCTCCTGCAAAGAAGGTAAACTCGGGCACAGCAACAGTAGCAATAGCTTGGACAATGGGAAATATGTGAGGTA

CACGCCTGAGCAGGTTGAGGCCCTCGAGAGGCTCTACCACGAGTGTCCGAAGCCCAGTTCACTCCGTCGCCAACAGCTGA

TCAGGGAGTGTCCCATTCTCTCCAATATTGAGCCCAAGCAAATCAAGGTCTGGTTCCAGAACCGAAGATGCAGGGAGAAG

CAGAGGAAAGAAGCTTCCCGTTTGCAAGCTGTGAACAGGAAGCTCACTGCGATGAACAAGTTATTGATGGAGGAGAATGA

TAGGTTGCAGAAGCAAGTTTCTCAGCTGGTGTATGAGAATGGCTATTTCCGCCAACACACCCAGAACACGACGCTTGCAA

CCAAAGACACAAGCTGTGAATCGGTGGTGACGAGCGGTCAACACCAGTTGACATCTCAGCATCCTCCCAGGGATGCTAGT

CCTGCAGGGCTTTTGTCCATTGCAGAAGAGACTTTAGCAGAGTTTCTTTCAAAGGCCACTGGAACCGCTGTGGAGTGGGT

CCAAATGCCTGGAATGAAGCCTGGTCCGGATTCCATTGGAATCGTTGCTATTTCTCATGGTTGCGCTGGCGTGGCAGCAC

GAGCATGCGGACTTGTGGGTCTTGAACCTACAAGAGTTGCAGAAATCCTAAAGGATCGACCGTCATGGTTCCGTGACTGT

CGAGCCGTGGATGTTTTGAACGTGTTGCCAACAGCAAATGGTGGAACCATTGAGCTGCTCTACATGCAGCTCTATGCGCC

AACAACCTTGGCGCCAGCCCGTGACTTCTGGTTGCTGCGTTATACTTCTGTTCTGGAAGATGGGAGTCTCGTGGTGTGTG

AGAGGTCACTTAAAAATACACAAAATGGTCCAAGCATGCCTCCAGTACAGCCTTTTGTCCGAGCAGAGATGCTCCCTAGT

GGCTACTTGGTACGTCCATGTGAAGGTGGTGGTTCAATCATACGCATTGTTGATCACTTGGATCTAGAGCCATGGAGTGT

GCCTGAAGTACTGCGACCATTGTATGAGTCCTCCACAATGCTTGCTCAGAAGACGACAATGGCAGCTCTGCGACAGCTGA

GGCAGATAGCTCAGGAAGTTTCACAGCCTAATGTTTCTGGCTGGGGAAGGCGACCTGCAGCACTTAGAGCTCTTAGCCAG

AGGTTAAGCAGGGGATTTAATGAGGCTCTTAATGGATTTACTGATGAAGGATGGTCGATCATGGGGAATGATGGCATTGA

TGATGTCACTATTCTCGTGAATTCATCCCCTGACAAGCTAATGGGATTGAATCTTTCGTTTTCAAATGGATTCCCAGCTG

TGAGCAACGCTGTTCTATGCGCGAGGGCCTCTATGCTCTTGCAGAATGTGCCTCCTGCAGTCCTCCTTCGCTTCCTCCGT

GAGCACAGGTCAGAATGGGCTGACAACAGTATTGATGCATACTCAGCCGCAGCAGTTAAAGTTGGTTCCTGTGCTTTACC

TGGATCACGTATTGGGAGTTTCGGGGGTCAGGTTATACTTCCACTTGCTCATACTATTGAGCATGAAGAGTTCTTGGAGG

TCATCAAATTAGAAGGTATGGGCCACTCTCCAGAAGATGCCTTAATGCCTAGAGATATATTTTTCCTGCAAATGTGCAGT

GGAGTGGATGAAAATGCTGTGGGAACATTTGCCGAATTGATATTTGCTCCAATTGATGCTTCCTTTGCTGATGATGCACC

TCTTCTTCCTTCTGGGTTTCGTATCATTCCTCTTGATTCAGTAAAGGAAGCTTCTAGCCCTAATCGCACATTGGACCTTG

CCTCTTCTCTTGAGATCGGGCCAGCTGGAAATAGGAGTTTTAATGATATTAATGCTAATTCTGGTTGTACGAGATCAGTG

ATGACTATCGCATTTGAGTTTGCATTCGAAAGCCACATGCAGGAACATGTGGCCTCTATGGCCCGCCAATATGTGCGTAG

TATAATATCCTCGGTGCAGAGAGTGGCATTGGCACTCTCTCCTTCCAATCTCGGTTCACATGCTGGTCTGCGTACACCTC

TTGGCACTCCTGAAGCCCAAACACTTGCTCGCTGGATTTGCCACAGTTATAGGTGCTACTTGGGGGTGGATCTTCTCAAG

TCCAGCAATGAAGGAAGTGAGTTGATTCTCAAGAACCTGTGGCATCACTCAGATGCTATTATGTGCTGCTCTCTTAAGGC

CTTACCCGTATTCACGTTTGCAAATCAGGCAGGTCTGGACATGCTCGAAACCACCTTGGTGGCGCTGCAAGACATAACCC

TGGAAAAGATTTTTGATGATCATGGCCGAAAGACTCTGTGTTCAGAGTTCCCACAAATCATGCAACAGGGTTTTGCTTGT

CTTCAAGGTGGGATCTGCCTCTCGAGCATGGGACGACCAGTGTCATACGAAAGGGCAGTGGCGTGGAAAGTTATGAATGA

GGAAGAGAATGCCCACTGCATCTGCTTTATGTTCATCAACTGGTCTTTTGTGTGA

>EgHD-Zip20

ATGGCGGTGACTTCGGCCTGTAAGGACAAGATGGGGATCGACAACGGGAAGTATGTGCGGTACACGCCCGAGCAGGTCGA

AGCTCTCGAGAGGCTGTATCACGAGTGCCCGAAGCCGAGTTCACTGCGGCGACAGCAGCTGATCAGAGAATGCCCGATTC

TCTCCAATATCGAGCCGAAGCAGATCAAAGTTTGGTTTCAGAATCGAAGGTGTAGAGAGAAGCAGAGAAAAGAAGCATCT

CGCCTTCAGGCAGTGAATAGGAAGCTGACTGCAATGAACAAACTTTTAATGGAGGAGAACGATAGGCTGCAAAAGCAAGT

GTCACAGCTGGTGTATGAGAACAGTTATTTCCGCCAGCAGACACAAAACGCAACCCTCGCCACCACTGACACGAGTTGTG

AATCGGTGGTGACCAGCGGTCAGCACCATTTGACTCCTCAGCATCCACCAAGGGATGCCAGCCCTGCAGGACTTTTGTCC

ATTGCAGAGGAAACTTTAACAGAGTTTCTTTCGAAGGCCACTGGAACTGCTGTGGAGTGGGTCCAATTGCCTGGGATGAA

GCCTGGTCCGGATTCCATTGGAATCATTGCTATTTCTCACGGATGCACTGGTGTGGCAGCACGTGCATGCGGCCTTGTGG

GTCTAGAACCTTCAAGAGTTGCTGAAATCCTCAAAGATCGGCCGTCGTGGTATCGTGATTGCCGAGCTGTGGATGTCGCA

AATGTGCTGTCTTCTGGAAACGGTGGGACTGTCGAATTGCTGTACATGCAGCTCTATGCGCCTACCACTCTGGCACCGGC

CCGTGACTTCTGGTTGCTGCGCTACACATCTGTAATGGAGGATGGGAGTCTTGTGGTATGTGAAAGATCAATTAACAACA

CCCAAAATGGTCCTAGTATGCCTCCTGTGCAGCATTTTGTGAGAGCTGAAATGTTGCCTAGTGGATATTTGATCCGGCCC

TGTGAAGGGGGTGGGTCGATAATTCATATCGTTGATCACATGGATTTAGAGCCATGGAGCGTGCCTGAAGTATTGCGCCC

ACTTTACGAGTCGTCAACTTTGCTCGCACAAAGGACAACGATGGCGGCTTTACGCAATCTGAGGCAGATATCTCAAGAAG

TTTCCCAGCCAAATGTCACTGGTTGGGGAAGAAGACCTGCGGCACTGCGTGCTTTAGCTATCTATTTGGACAGGGGTTTT

AACGAAGCTGTCAATGGATTTATGGACGATGGTTGGTCTATGTTGGAAAGTGATGGCGTCGATGATGTTACTCTTCTCAT

TAACTCATCGCCGGCCAAGATGGCAGGCGTGAACATTTCTTACGCAAGTGGTTTTCCTTCAATGACTAGTGCGGTCTTGT

GTGCAAAAGCATCCATGCTGCTGCAAAATGTGCCTCCAGCAATACTCTTACGGTTTTTGCGAGAGCATCGATCAGAGTGG

GCAGACAGCAGTATTGATGCATACTCTGCTGCGGCAATCAAAGCCAGTCCATGTAACATGCCAGGGACACGAATTGGAGG

GTTCGGGAGTCAGGTTATTCTTCCGCTCGCTCACACAATTGAGCATGAAGAGTTTATGGAAGTTGTTAAACTTGAAAATA

TGGGCCATTATCGAGACGACATGATTATGCCAAGCGACATCTTCCTCTTGCAACTTTGCAATGGAGTGGATGAAAATGCT

GTTGGCACTTGTGCTGAACTACTTTTCGCTCCAATTGACGCATCCTTTTCTGATGATGCACCCATTATTCCTTCGGGATT

CCGCATCATTCCTCTTGATCCAGGATCGGATGCCTCCAGCCCAAACCGGACACTTGATCTTGCCTCAGCTCTTGATGTTG

GTCCCACAGGCAACAAAGCGGTCGGTGATAATTCTGGTCATAGTGGAAACACCAAATCTGTGATGACTATAGCCTTCCAA

TTCGCATTTGAATTACATCTTCAAGAGAATGTGGCGTCCATGGCTCGTCAATACCTCAGAAGTATTATAGCATCTGTTCA

GAGAGTGGCATTAGCGCTCTCACCCACAAATTTGGGCTGTCATGCGAGTCTTCGCCCCCCACCTGGCTCTCCTGAAGCGC

ACACGCTTGCCCGTTGGATCTGCCAAAGCTACAGGTCCTTTCTTGGTGTGGCATTGCTCAAGAACGAGGCCGCTGATTCT

TTGCTAAAAAATCTGTGGCATCACTCAGATGCAATCCTATGCTGCTCTCTCAGGGCAGCACCGGTTTTCACATTTGCTAA

CCAAGCAGGACTTGACATGTTGGAAACGACGTTGATTGCACTGCAAGACATTACCCTGGAAAAGATTTTTGACGACAATG

GAAGAAAGACTCTCTGCTCAGAATTCCCCCAGATAATGCAGCAGGGTTTCATGTGTCTCCAAGGTGGCATTTGCTTGTCA

AGCATGGGGAGGCCGATTTCGTACGAAAGAGCAGTAGCTTGGAAGGTGTTGAATGAAGAAGAGACTGCTCACTGCATCTG

CTTCATGTTTGTCAATTGGTCTTTTGTCTGA

>EgHD-Zip21

ATGAGAATCACCATGGACAACGACCACCAACTAGAAGAACATATGGCTCTTATCTCTGAGCTGTACCCGGGGGTTTACAC

GCAGATCGTACCGCCCGTTAAAGAGTCGAAGCCACGGCGCAGGAGGAAGAAGAGCAAGGGCGAGGGCGGCGCAGCGGGGC

CGAGGAAGAGGAAGCTGAGCGAGGAGCAGGTGAACCTGCTGGAGCAGAATTTCGGGAGCGAGCACAAGCTGGAGTCGGAG

AGGAAGGATCGGCTGGCCTCGGAGCTCGGGCTCGACCCGAGGCAGGTGGCCGTCTGGTTCCAGAACCGGCGCGCCCGGTG

GAAGAACAAGAAGCTCGAGGAGGAGTACGCGAAGATGAAGTCGGCGCACGAGGGCACCGTCATCGAGAAGTGCCACCTCG

AGTCCGAGGTACTGAAGCTGAAGGACCAGCTGATCGAGTCGGAGAAGGAGATCCAGCGGCTGCTGGAGCGGGCCGACGGG

CCCGCGGCGTCAAGCAACAGCCCGAGCTCGTCGATGTCGATGGACGCGAGCATGGACAGGCGGTTCCCGGGGGAGTTCGC

GTACGAGGACGTTTTCTACATGCAGGAGAACGTATACAATGTCTACGGCATGGACTGGATGAACCTTTTTGACTCGCGCC

AATGA

>EgHD-Zip22

ATGGGCATAGATGATCTGTGCAACACAGGCCTTGTTCTGAGTCTTGGCCTCGAGACGCCCTTCAAGATCGAAGCGCAGAG

GCAAGCCAAACAGCGCCTTAACTTCGAGCCCTCTCTCACGCTGTGCCTCTCCGGCACGACCAAAGCTACCCGCGACGAGC

AACCTCCGGCGGACCACTTGTATCGCCAGGCTTCGCCGCACAGCCACAACAGCCTCAGCGCGGTGTCGTCGTTCTCGAGT

CCGCGGGTGAAGAGGGAGAGGGACCTCAGCAGCGAGGAGGCCGAGGTCGAGACGCGGGTGTCATCGAAGGCGAGCGACGA

GGACGACGACGGCGCGAATGCGAGGAAGAAGCTCCGGCTCACTAAGGAACAGTCTGCTCTTCTGGAAGAGAGCTTCAAAC

AGCATAGCACTCTCAACCCTAAGCAAAAGCAAGCTCTAGCGAGGCAGTTGAATCTACGGCCCCGCCAAGTCGAAGTGTGG

TTTCAAAACAGGAGAGCCAGGACGAAGCTCAAGCAGACCGAAGTGGACTGTGAGTTCCTCAAAAAGTGCTGTGAGACGCT

GACCGACGAGAACCGGCGATTGCAGAAGGAGCTCCAAGAGCTTAAGGCCCTGAAACTGGCCCAACCCTTTTACATGCACA

TGCCCGCGGCGACCCTCACCATGTGCCCCTCGTGCGAGCGGATTGGTGCGGGCCCGAGCGTCGACGGCGCGGCGCCGACG

AAGGGCCCTTTTTCGATGACGACAAAATCACACTTATACAGTCATCACTTTACCAATCCATCTGCTGCTTGCTGA

>EgHD-Zip23

ATGGCGCTGGCGATGCACAGGGAGTGCTCGAGCAAGCAGATGGACGCGAGCAAGTACGTGCGGTACACCCCCGAGCAGGT

GGAGGCGCTGGAGCGGGTCTACAACGAGTGCCCCAAGCCCAGCTCGCTGAGGCGGCAGCAGCTGATCCGAGAGTGCCCCA

TCCTCTGCAACATCGAGCCCAAGCAGATCAAAGTCTGGTTCCAGAATCGCAGATGTCGGGAAAAGCAGAGGAAGGAAGCA

TACCGTCTCCAAACAGTTAACAGAAAGCTGAATGCCATGAACAAACTGTTAATGGAAGAGAATGATCGCCTCCAGAAACA

AGTTTCACAGTTGGTTTATGAGAACGGCTACATGCGGCAGCAGCTGCATAGTCAGGCATCAACAGCGACCACAGACAATA

GCTGTGAGTCTGTGGTCATGAGCGGTCAGCACCAACAGCAGCATAACCCAACACCCCAGCATCCAGAAAAGGATGCCAGC

AGCCCAGCTGGCCTTCTTGCGATTGCTGCGGAGACCCTCACACAGTTCCTGGCCAAGGCTACTGGAACTGCTGTTGACTG

GGTACAGATGATTGGGATGAAGCCTGGTCCGGATTCTATTGGAATCGTTGCTGTTTCCCGCAATTGTAATGGGGTAGCAG

CACGAGTCTGCGGTCTTGTGAGTCTAGAGCCCATGAAGGTCGCTGAAATTCTCAAAGATCGTCCATCTTGGTTTCGCGAC

TGTCGATGCCTTGAGGTTCTTAATGTGATCCCTGCAGGGAATGGGGGGACGATAGAGCTTGTATACATGCAGACCTATGC

ACCTACAACTCTGGCAGCAGCACGTGATTTTTGGACAGTTCGATATACCACTACTCTGGAAGATGGCAGTCTTGTGATAT

GTGAGAGGTCATTGACTACAAGTACTGGTGGCCCATCAGGGCCGTCGTCTTCAAGTTTTGTGAGAGCTGAAGTTCTTCCA

AGTGGTTATCTCATCCGACCTTGTGAGGGAGGTGGTGCAATCATTCATATTGTGGATCATGTTGATCTAGATGCTTGGAG

TGTTCCTGAAGTTCTCAGACCACTTTATGAATCGTCCAAAATTCTTGCACAGAAGATGACTGTTGCTGCTTTGCGCCATA

TTAGACAAATAGCCCAAGAAAGTAGTGGGGAGATTCAGTATGGAGGTAGCCGACAACCTGCAGTCTTGAGGACGTTTAGT

CAGAAATTGTGCAGGGGGTTTAATGATGCTGTGAATGGCTTTGTGGATGACGGTTGGTCTGTTCTAAGTAGCGATGGGGT

AGAAGATGTCACCATTGCTGTCAACTCATCTCCAAATAAATTTCTTGGTTCCCAATACAATGCAACCATATTTCCAAATT

TTGGAAGAGGAGTGCTCTGTGCCAAGGCGTCCATGCTTCTTCAGAATGTTCCGCCTGCTGTGCTTGTACGCTTTCTGAGG

GAACACCGCTCTGAGTGGGCTGACCATGGAATTGATGCATACTCAGCTGCATCTTTGAAAACTAGTTCTTATGCCATTCC

ATGTGTGAGACCTGGTGGTTTCCCTAGTAGCCATGTCATTTTGCCTCTTGCCCACACTGTTGAACAGGAGGAGTTCTTGG

AGGTGGTTCGGCTAGAGGGTCATGCATTCTCACCTGAAGATATACCTTTGGCACATGACATGTATTTACTTCAGTTATGC

AGTGGGGTGGATGAAAATGCAGTTGGTGCCTGTGCTCAGCTTGTCTTTGCTCCTATTGATGAATCTTTCGCAGATGATGC

TCCTTTGTTGCCATCTGGCTTTCGTGTCATACAATTGGATCCTAAAACAGATGGGCCTGCCCCTACTCGGACATTGGACC

TGGCCTCTACGCTGGAGGTGGGATCTGGCGGTGCTCGTCCTACTTGTGAAGCTGATGCTAGCACCTACAACCTGCGATCT

GTCCTGACCATCGCATTCCAGTTCGTGTTTGAGAACCATTTACGGGACACTGTTGCCATCATGGCTCGTCAATATGTGCG

TAGTGTTGTGGGATCTGTCCAGAGGGTTGCCATGGCAATTGCACCTTCCAGGCTAGGTGGCCATCTGGGGCCAAAATCTC

TCTCTGGTTCTCCTGAAGCTCTTACGCTGGCACGATGGATCTGCCGTAGCTACAGAATTTGTGCTGGAGCTGAACTGTTG

AGAGGGGACTCCCAAGCTGGTGATGCTGTTTTGAAGGAATTTTGGCACCATTCTGATGCAATTATGTGCTGCTCTGTGAA

TACAAATGTGGCCTCTCCTGTCTTCACCTTCGCCAACCAAGCTGGACTTGACATGCTTGAAACTACTCTGGTGGCCCTCC

AAGATATTATGCTGGAAAAGGTTCTTGATGAAGGTGGCAGGAAAGTTCTTTCTTCGGAGTTCCCGAAGATCATGCAGCAG

GGTATCGCCTATCTGCCAGCCGGAGTGTGCATTTCTAGCATGGGAAGGCCGGTGGCTTATGAGCAAGCCGTCGCATGGAA

AGTTCTCAACGACGACGATTCCCACCACTGTCTGGCCTTCATGTTCATGAACTGGTCTTTTGTGTAG

>EgHD-Zip24

ATGATGGTGGAGAGGGAAGATCTGGGCTTGTCTCTGAGCTTGAGCTTCTCTGACAGCAGTCGGCCTTCCCAGCTCGGCGC

CTCTCCTTTCGGGTTCAACCTCTACAAGCCATCTCACCGCGACTGCGAGACCTTCGCTTCATTAGATCGGATCTCGGAGG

CGGATGCGCGGCCGTCCCTGCGGGGCATCGACGTGAACCGGCCGCCGCCGTCGGCGGCGGACTGCGAGGAGCAGGAGGAG

GCGGGGGTGTCGTCCCCGAACAGCACTATCTCGAGCGTCAGCGGGAAGAGGGGCGAGAGGGAGATGGTCAGCGGCGGGGA

GGACAACGAGGCGGAGAGGGACTGCAGCCGCGGAGGCAGCGACGAGGAGGACGGCGAGAACTCGAGGAAGAAGCTGAGGC

TGTCCAAGGATCAGTCGGCCGTCTTGGAGGAGAGCTTCCGAGAGCACAACACTCTGAATCCCAAGCAAAAGCTGGCGTTG

GCTAAGCAGCTAGGCCTGCGACCTAGACAAGTAGAAGTCTGGTTTCAGAACCGCAGAGCACGGACCAAGTTGAAGCAGAC

GGAGATAGACTGCGAGTTCCTGAAGAGGTGCTGCGAGAACCTGACGGAGGAGAACCGGCGGCTGCAGAAGGAGGTCCAGG

AGCTGCGCGCTCTCAAGCTCTCCCCTCAGTTCTACATGCACATGCCCCCTCCCACCACCCTTACCGTCTGCCCCAACTGC

GAGCGCGTCGGGGCGGCCGCACCGCCGCTCCCATCCGCCGGCGGCGGCGGCAGGCCCGCCCACCATCGGGAGCCGGTGCC

CATGATCCCATGGGCCGCGCGGCCCGGCCCGGTCTCGCACGGGGCTCTCCGGCCCAGGACGTGA

>EgHD-Zip25

ATGGGAGACATTGAGGAAGCATGCAACACGGGGTTGTTTCTAGGGTTAAGCGTTGGTGGGAGCACGACGAAGCGCAAGGA

AGAGAAGAAGAAGAACAAGCAGAATAACGACGCTCCTTCGACGGTGGGCCTGGGGCTTTCCTTCTCCATTAACTGCTCGT

CATCAAAGGTGGAAGCTGATGATCATCCCTGGGACAGCATTAACAACGCCGAATATCCAGCCAGGTCAAGCTTCCACAAC

AGCTTTGTTGACCAATCTCCAGATGACCACATCAGTCCACCTCGTACCAGCAACGACGACGGCGGAGATTTTAACGGTGG

CCGGAAGAAACTCAGATTGACGAGAGAACAGTCTGCCTTGCTCGAAGAAAGCTTCAAACTTCACACCACTCTCACACCGG

CACAGAAACAATCATTGGCTGACCAACTCAAGTTGAAGCCTCGGCAAGTGGAAGTTTGGTTTCAGAATAGGAGAGCAAGG

AGCAAACTGAAGCAGACAGAAGTGGACTGCCAGTTCTTGAAAAAATGCTGCGAGACCTTGAGCGACGAGAACAGGAGGCT

GAAGAAAGAATTGCAGGAGTTGAGATCGTCATCGTCGCCATATGTCCAAGGACCGGGGACAGCGGCAGTGATGTTATGCC

CTTCTTGCGAGGAACTCACGAGAGGTAATTCTGAAGCCACGAGGAAAGAGAACTGCGATATCTGA

>EgHD-Zip26

ATGGCCGGTGAGGAGCCCTATTCTGCCGACACGAACTCGGACACTTTCGCTGATGAAGAAACGCTGATTCCGAGTTCTTC

CGAGGCTCTTGAGTCCGCCTGGGTTCCTACTTCCTCGACCGCTCATCATGGTTCAAAATCAGTGGTCAATTTTGAGGACG

TTTGTGGAGGAGGAGACACCAATACTGCGCCGAGGCCATACCTCCGACAGATTGATCTGAAGGAAGAAGCCGTCGAAGAG

GACTACGGCGACGGGAACTTTCAGCCTCCTGGTAAGAAGCGGCGGCTATCGGCCGACCAAGTCCATTTCCTCGAGAGGCA

CTTTGAGGTCGAGAACAAGCTCGAGCCCGAGAGGAAGATCCAGCTCGCCAAGGACCTCGGCCTGCAGCCGAGGCAGGTCG

CGATCTGGTTCCAAAATCGGCGAGCCCGGTACAAGACCAAGCAGCTGGAGAAGGATTATGATTCGCTCAAAGCTCGCTTC

GAGAGCCTCAAGGCCGATCACGACAAGCTTATCAAAGAAAAGGAGAATTTGAAAGGGGAGGTCCTGTCTCTTAGAGACAA

GCTGAGGAGCAGAGCAAAAGGAAGCGAGGAGGGATCGCTCGAAGCTGATGATTCCTTGCGTGGAGCGACTCCGAAACCAA

CCACACCCTCAACTCTCGATGACGTTTCGTACGTGGATGCCACGATCTTAAAGCAGGAGGACGTGAGTTCGGCCAAGAGC

GATGTGTTCGATTCGGACTCGCTGCTGGAACCTGCGAATTCTTCCCACGTGTTCGAACCAGACTGCTCGGACTTCTCGCA

GGACGAGGATGACAGTTTCGGCAAGAGCCTCTTGCCCCTGGATTTCCTCCCCAGATTTGATGATGGATACGCCTTCCAAC

AGAACCCACCTGCAATTTCTTGCAATTTTGCTTTCCCAGCTGAAGAAAATCCCTTCTGGTCCTGGTCTTACTGA

>EgHD-Zip27

ATGGGTGAGCAAGATGATGGGCTGGGGTTGAGCTTGAGCCTGGGATGCGGGAAGAGCGCATTGTCTTTGAACCTCAATCT

CGTGGAAGCTCCTGTGCAGTCCAGGCAGAGTCCTGGGCAAAAGTGCTCTTGGAATGAGATTTTCCACTCTCCTGAAACAA

ACTCCAAAACAAGGCCATTATTCGGTGGAGTCGGCGTCAACCGAGACATACTGGTGGTGGATTTGGATGATGATAATCAT

GTCTCATCACCTAACAGCACCGTGTCGAGCATAAGTGGGAAGAGAAGTGAGAGAGACCCGCACGGAGATAATGAAGTTGA

GGCTGAGAGAACTTCGTGCTCTTGTGAGAGCATCGAAGAAGATGGCAGTGGAGGAGATGGAGCAAGGAAGAAGCTCAGGC

TGTCTAGAGAACAGTCATTGGTGCTAGAGGAGACATTCAAAGAGCATAACACTCTTAACCCTAAGCAAAAGCTTGCCTTG

GCCAAGCAGCTGAATCTTAGTCCCAGACAGGTAGAGGTCTGGTTTCAGAACAGGCGTGCCAGGACTAAGACGAAGCAAAC

TGAGGTGGACTGTGAGTACCTTAAGAGGTGCTGCGACAATCTCACACAGGAAAACAAGAGGCTGCAGAAGGAAGTGCAGG

AGCTCAGAGCACTGAAGCTCTCCCCACAGCTCTACATGCATATGAACCCGCCCACCACGCTCACCATGTGCCCTTCATGT

CAGCGAGTGTCTGTTCCTTCGTCATCGCTGTCGCCTCCATCTTCATCTTCAGTCACTGCCTCAGTGATTGGCCCCGTGGG

GCCGGTCCATAATCCTTTTAGTCAGTCTGGACCATCCATCAACCCGTGGGCAAGGCTACAGATGCAACAAGGACTGAACA

ATTTGCACTCTTGA

>EgHD-Zip28

ATGTGCCCTATCGATTCGGGCCGCTCCTTCGACACGAGCCTTAGTCTCGGTTTAGGCTGTTATGGGGATCCTGAAGATCA

CGAGATCAAGATCAAGAAACCGCTCGCGAAACTCAGTGGTAACTCCACGTGCCTCACGATAGGCTTGCCCGGCGGGGAGG

CGTGCGGGCTGGGATCCGCGAGTGGGGACGAGGTCAGGAACATCCCGAGCAGGTCGGCATCGTCGTTCTCAAACTCAAGC

AGTGCGAAGAGGGAGAAGGCGGAGCAAGGAGAGGAAGAAGCGGTTGAGAGAGGGACGGGCTCGCCGAGGGCGACTATCAA

TATCGAAGATGAAGATGAGTTCAGCCCCAGGAAGAAGCTCAGGCTTTCTAAAGCACAAAGTTCCATTTTGGAAGAGAGCT

TCAAAGCGCACACAACCCTCAACACTAAACAAAAGCACGATTTGGCAAATCGGTTGAATCTCCGGCCACGGCAAGTGGAA

GTGTGGTTCCAGAATAGGAGAGCCAGGACTAAATTAAAACAAACTGAAGTAGAGTGCGAGATGCTGAAGAAATGCTGCGA

AACCCTAAAAGAAGAGAACAGAAGGTTGAAGAAGGAGTTGCAAGAGCTCAAGTCATTGAAGCCAACTGCGTCGGTTTATA

GGCAGATTCCTGCAGCTGCTCTCCCTCTATGCCCTTCTTGTGAAAGGATTGCCCATCCAGAATTCCCGTTCTCGACTGAA

TCTCGACTTTGGCCTGCTCATCCATCAGCCGCTTGTTAA

>EgHD-Zip29

ATGAAGAGACTTGGCAGCTCAGATTCGTTGGGTGCTTTGATGTCCATCTGCCCACCTTCAGAGGAATTGCAGCACAGTCC

GAGAAACGGCAACCCCATCTACCACAGCAGGGACCTGCAGTCCATGCTGGAGCTGGGCCTCGACGAGGAAGGCTGCGTGG

AGGACCAGTCCGCCGGCGGCGGGGGGCACGTCGGCGGCGAGAAGAAGCGGCGGCTGAGCATCGACCAGGTCAAGGCCCTG

GAGAAGAACTTCGAGGTGGAGAACAAGCTCGAGCCGGAGCGGAAGGTGAAGCTGGCCCAGGAGCTGGGGCTGCAGCCGCG

CCAGGTGGCCGTGTGGTTCCAGAACCGCCGCGCGCGGTGGAAGACGAAGCAGCTGGAGCGGGACTACGGCGTGCTCAAGT

CCAGCTACGAGGCGCTCAAGCTCAGCTACGACGCCCTCAAGCACGACAACGAGGCCCTTCACAAGGAGATAAAAGAGCTG

AAATCGAAACTCCGGGAAGAAGACGACAACCCCGAGAGCAATCTCTCCGTCAAAGAAGAGGTCATCATCCCCAGCCACGA

CGTGTCGGACAAGATCCGGGCCGCGGACGACGGCGACGACGACACCAAACGCTCTCCTCCCCCTCCGATCACCGCCCCGC

CTCGCGAGCTGAGCTTCAACAATGGTGGGCTGAAGGACGGGTCGTCCGACAGCGACTCGAGTGCAATTGTGAACGAAGAG

AACGCGGCGACCAGCAGCAGCAGCCCGAACCCCGCCGTCCAGAGCCACGGCGGCTTCTTGAAATTCATGGGGTCATCGTC

CTCTTCGGCCTCCCCACCGCCGCCGCCACCGGCTTCCTTCGGCGGGTGCTTCAGCTTCCAGTTCCAGCGAGCGTACCAGC

CTCAGCCTCAGCCTCCTCACCACCACCACCACCACAGTCCGTACGTGAAGATGGAGGAGCACAATTTCCTCGGCGGCGAG

GAGGACTGCAACTTCTTCTCCGACGAGCAGGCTCCGACGCTGCATTGGTACTGCCCCGATCAGTGGAACTGA

>EgHD-Zip30

AGCATAAGTGGGAAGAGAAGTGAGAGAGACCCGCATGGAGAAAATGAAGTTGAGGCTGAGAGAACTTCGTGCTCTTGTGA

GAGCATCGAAGAAGATGGCAGCAGAGGAGATGGAGCAAGGAAGAAGCTCAGGCTGTCTAGAGAACAGTCATTGGTGCTAG

AGGAGACATTCAAAGAGCATAACACTCTTAACCCTAAGCAAAAGCTTGCCTTGGCCAAGCAGCTGAATCTTAGTCCCAAA

CAGGTAGAGGTCTGGTTTCAGAACAGGCGTGCC

>EgHD-Zip31

ATGGCGATGACGATGGTGCCTCACAGGGAGAGCAGCAGTGGAAGCATCAACAAGCACTTGACCGACTCGGGCAAGTACGT

GAGGTACACAGCGGAGCAAGTGGAAGCTCTCGAGAGGGTCTATTCAGAATGCCCTAAGCCCAGCTCTCTGCGCAGACAAC

AGCTGATTCGGGAGTGCCCCATTTTGTCCAACATCGAGCCTAAGCAGATCAAAGTCTGGTTTCAGAATCGCAGGTGTCGA

GAGAAGCAGAGAAAAGAGGCCTCAAGACTCCAGACGGTGAACAGGAAACTGACGGCCATGAACAAGCTCTTGATGGAGGA

GAATGATCGGCTGCAGAAGCAGGTTTCGCAATTGGTATGCGAAAATGGCTACATGCGTCAGCAACTCCATACTACATCGG

CTACTACTACCGATGCAAGCTGCGACTCTGTGGTTACTACTCCTCAGCACTCTCTCAGAGATGCAAATAACCCTGCTGGG

CTCCTCTCCATTGCGGAGGAGACATTGGCGGAGTTCCTCTCCAAGGCTACAGGAACTGCTGTCGATTGGGTTCAAATGCC

TGGGATGAAGCCTGGTCCGGATTCGGTTGGAATCTTCGCCATTTCACAAAGTTGCAGTGGAGTGGCCGCTCGAGCCTGTG

GTCTTGTTAGTTTAGAACCAACCAAGATTGTAGAGATCCTCAAAGATCGTACTTCATGGTTCCGGGACTGTCGGAGCCTT

GAAGTCTTCACCATGTTTCCTGCTGGAAATGGTGGAACAATCGAACTTGTTTACACACAGATTTATGCTCCAACTACTCT

GGCTCCTGCACGAGATTTATGGACTCTCAGATATACCACGACTCTAGAAAATGGCAGTCTTGTGGTATGTGAGAGATCTC

TGTCCGGATCTGGTGCTGGCCCCAACCCAGCTTCAGCAACTCAATTTGTGAGAGCTGAAATTCTTCCTAGTGGCTATTTG

ATTCGCCCATGCGAGGGTGGAGGATCAATCATTCACATTGTCGACCACCTCAATCTCGAGGCCTGGAGTGTTCCTGAGGT

TCTTCGGCCACTTTATGAATCATCCAAAGTTGTGGCCCAGAGAATTACTATTGCGGCTCTTCGATACATCCGGCAAATTG

CTCAGGAGACAAGTGGGGAGGTGGTTTATGGTTTGGGAAGGCAGCCGGCAGTTTTGAGAACTTTCAGCCAAAGATTGAGC

AGAGGCTTTAATGATGCAGTAAATGGATTCAATGACGGTGGGTGGTCATTGATGAATGGTGACGGAGCCGAAGATGTCAT

GATTGCTGTCACTTTCTCTAAAAAATTGAACACTACATCAAATCCTGCTAATCCCCTTTCATTTGTGGGAGGAATTCTAT

GTGCCAAAGCTTCCATGTTACTACAAAATGTCCCTCCTGCTGTGTTGGTTCGGTTTCTGAGGGAGCACCGCTCAGAATGG

GCTGATTTCAATGTGGATGCTTATTCTGCAGCATCATTAAAAGCTAGTCCATTTGGCTATCCTGGTATGAGGCCCACAAG

ATTCACCGGGAGTCAAATCATCATGCCCCTTGGTCACACAATTGAGCACGAAGAGATGCTTGAAGTTATCCGTCTTGAAG

GCCATTCTCTTGCTCAAGAAGATGCTTTTGTATCAAGAGATATTCATCTTTTGCAGATATGCAGCGGGATAGACGAGAAT

GCAGTTGGAGTCTGTTCCGAACTTATTTTTGCGCCAATTGATGAAATGTTTCCTGATGATGCTCCACTGCTACCCTCTGG

TTTCCGTATCATACCACTGGATTCAAAATCATCTGATGTACAGGATTCTCTAACGACAAATCGGACCCTTGATCTGACAT

CGAGTCTTGAGGTGGGGCCTGCATCAACAAATTGCGTTGGAGATGTTGCGCCAAGCCATGGTGCACGATCTGTTCTGACT

ATCGCCTTCCAGTTCCCATTTGATGCCAACACACAGGATAATGTGGCAGTCATGGCCAGGCAGTATGTCCGTAGCGTTAT

TTCGTCTGTGCAGCGGGTTGCGATGGTCATATCTCCATCAGGATTGGGCCCTTCCATTAACCCCAAGCTTTCACAGGGAT

CTCCAGAAGCTCTAACACTTGCTAACTGGATCTGCCAGAGCTACAGCTTATATCTGGGAACAGAATTGCTGGGATCTGAT

TTGCTTGGTGCTGATTCTATGTTGAAGACTCTTTGGAGTCATCAGGATGCCATATTGTGTTGTTCATTGAAGTCTATTCC

AGTTTTCATCTTTGCAAACCAGGCAGGACTTGACATGTTAGAGACCACTTTGGTGGCTTTACAAGATATCACTTTGGATA

AGATATTTGACGAGTCAGTCCGCAAAGAATTGTCCCCCGAGTTTGCCAAGTTAATGCAAGAGGGGTCTGCCTACTTGCCC

TCTGGAATTTGCATGTCGACGATGGGGCGACATGTCTCATATGAACAAGCGATCGCGTGGAAGGTCCTTTCTGCGGAGGA

GAACACTGTCCACTGCCTCGCTTTCTCTTTCGTGAATTGGTCTTTCGTGTGA

>EgHD-Zip32

ATGGAGCTGGGTCTGAGCTTGGGAGATGCGCCGTTTCTTGAGAAGGAAGACTTAGGGTTTCATCTTGGGTTGGGAAGCAG

CTTCTGTGGACGGCCCAAGGAAGCAAGAGATCACGGCTGTGGCGATGTTGAGGATAAGAGTAAGCGGCCGAGAGACGCCG

TGGCTCTTCAGCTTCATCTCTTTCCTTCTCGAAGCCCGTCTTTGCTTCCTCAGCTTAACGATAATAACGGCGATTCTTCA

GATGGGCTAGCCGAAGGGAAGTGGTTGCCGAGCCGGTTGCTGGCGACTGCGGCGGCGGCAAAGACCGAGTTGGTGGATGC

TGGGGCGGAGCGGCTGTCTTCCCCGAACAGCGGCACATCTTCGTTTCATCAGCTGGAGTTCAGATCGGGCTCAGGCGGAA

GGAGCAAGACGGTCGACGCCTTGTCGTTGGAAGGCGACAACGAGAGGGCGTGTTCCAAAGGCAGCGATGAATATGATGAA

GACAACGGTTTGGCTCGGAAGAAGCTCAGGCTCTCCAAGGAACAGTCTGCTTTTCTTGAAGAGAGCTTCAAGGAACACCA

TACCCTTAATCCTAAAGAAAAGCTTGCATTGGCAAGACAGCTGAATCTGCGCCCTCGCCAAGTAGAAGTGTGGTTTCAGA

ACAGAAGAGCAAGGACGAAGCTGAAGCAAACGGAGGTGGATTGCGAGCACTTGAAGAGATGCTGCGAGAGACTGACAGAA

GAGAACAAGAGGCTGCACAAGGAGCTTCAAGAACTCAGGGCTCTCAAGTCCTCCCACCATTACTCGTTCATGAAACATCC

GGTTCCAGCCACCACTCTCTCCATGTGCCCCTCCTGCGAGCGCATCTCCACCTCCACTGCCGCCGCCACTAACATGAATA

AAATGCCTGCTCCATCGTCATCATTAACGTCGGCTAATCCTAGAACAGCCTTAATCCACCAGCCTCTCCCGAATAATACG

CAGGCTCAAGCTGCTTCCTGA

>EgHD-Zip33

ATGAAGCGGCTCAGTAGCTCGGATTCGTCGGATGTCTGGATCTCCATGTGCTCGGGTAAAGAGGAAAAAGTCCTAAAGAA

GAGCCAAGGCTACAGCATAGAATTTCAGGCAATGCTAGACAGTCTAGACCAAGAGGATCATTCCGGGGAAGAGGCCGGCC

TGATCACCGAGAAGAAGCGGCGGCTCAGCGCAAACCAAGTGAAGGCCCTGGAGAAGAACTTCGAGATCGAGAACAAGTTG

GAGCCGGAGCGGAAGGCGAGATTGGCTGAAGAATTGAGCCTCCAACCGCGGCAAGTCGCGATATGGTTCCAGAACCGGCG

CGCTCGCTGGAAGACCAAGCAATTGGAGAGGGACTTCGGCCATCTCAAGGCCAGCTACGACTCTCTGAAGCTCGACTTCG

ATAGCCTCGAGCAAGAAAAGGAGTCCCTAGCGGCAGAGCTAACGGAGCTGAAGGTGAAGCTCCGTCGAGAAACGTCAGAG

AGCAGCAATCACTGCGCAGTCAAGCATGAATCCCCCCTCTCCGAATCCTCCGAAGACGGCAAGCCCGGGTCGTGCAGCGG

ATCCGTCAAGGAAGACCCGAACCCGACCCCGGAGCTGCCCCCCTCGTCGGCTGCTCCCCCGCTCCCTCTCCGATACGGCA

GTTGCTCTACGTCCCCGCCGCCGCCGCCGCCGCCGCCGCCGCCTCCTTCGTCGTCAAGAGGGACCACGGCGGGAAGAGGG

TACTACCATCAGGTGAGGATGGAAGAGAATCATCCGAGCGGGTTCATTTCGGAGGAGTCCTGCAACTTCTTCTCGGTCGA

CCAACCTCCCACGCTGCACTGGTACTTCCCATGA

>EgHD-Zip34

ATGGAGCTAGCTCTGACTTTAGGGGACACGTCGAAGCCGTTCTCGTTCCTCGACAAGGCCCCCAAGATGGCCGGGGACAA

GGAGAGCGGCAACCGGGACCTGGGGTTCTGCATGGGGTTGGGGGTAAGTGGAAGGTCCTCGAGAGGCGGCGGCGACGAGG

AGGAGGACGGTGATGGTGACAAGAGAGAGAGTCCGGCGACGGATCGGCGCCACCACCAACAACACCATCCGCCTTCGACT

CTTCTTCAGCTCCATCTCTTGCCTTTCTCTCCGACAAATCCCAGGAACAGCCACCACAGCCACAAGCCCTCCACCTCTCA

GTTTCGGCTTCCGTGGCTCACCGATCACTTGGGGTCCGAGCCGGAATCTTCGGGCGGGGCCGGCAGGGGCCTGGACGTGA

ACCGGTTCCCGGCGGCGGAGGAGGCGGAGCAGTCGTCGCCGAACAGCGCGCTGTCGTCGTTTCAGATGGAGTTCGGCATC

AGAAGCGGCGGCGGGGCAGGATTTAGGAGCGGCAGCCACAAGAGGGTCGACATGGAAGGAGACAACGAGAGGGACTGTTC

GAGAGGCAGCGACGACGACGACAACGGCCTGACCCGGAAGAAGCTCAGGCTCTCCAAGGAACAGTCGGCTTTCCTCGAGG

AGAGCTTCAAGGAACACAACACCCTCAATCCCAAGCAGAAGCTAGCTTTGGCGAAACAGCTGAATCTCCGCCCTCGCCAA

GTCGAGGTGTGGTTCCAGAACAGAAGAGCCAGGACGAAGTTGAAGCAGACGGAGATCGACTGCGAGTACCTGAAGAGGTG

CTGCGAGACGCTGACGGAGGAGAACCGGCGGCTCCAGAAGGAGCTCCAGGAGCTCCGGGCGCTCAAAACCTCCCAGCCCT

TCTACATGCAGCTCCCCGCCACCACTCTCACCATGTGCCCCTCCTGCGAGCGCGTCGCCACCACCACCGCGCCCGCCTCC

GCCGCCTCCTCCCCAGCGGCGGCGGCGGCGGCCCCGCCCCTCGCCAAGCCGAAGCCCAGAACGTACCCGTTCCCCCACGC

CCCCCAGCTCCACCACCTCCCCCAGGCCCAACCCCAGGCCCACCAGGCCGCCTCCTGA

>EgHD-Zip35

ATGGCCTTCCCACCACCATCGGCATCCTCAGCTGCTAGTTTCATGTTCCAAGCCCACCACCATCACCACCAAGAAGATCC

GCTCCCTTCGATCATCCCGCTGAACCACAGCAACGGCGACGTTGTCAACAATGTGCCTTTCTTGATGAAGAGGTCCATGT

CCTTCTCAGGGGCCGACCACAACCAGAGATCGTGCGGGGGCGGCGACGACGACGACCTGTCGGATGATGGGTCGCAGCTG

CTTCTGGGGGAGAGGAAGAAGAGGCTGAGCTTGGAGCAAGTGAAGGCGCTCGAGAAGAGCTTCGAGATCGGGAACAAGCT

CGAGCCCGAGAGGAAGATCCAGCTCGCGAGGGCGCTCGGCCTCCAGCCGAGGCAGATCGCCATCTGGTTCCAGAACCGGC

GGGCTCGCTGGAAGACCAAGCAGCTGGAGAGGGACTACGAGGTGTTGAAGAAGCAGTTCGAGGTCCTGAAGGCTGAAAAT

GATGCTCTTCATGCCCAGAATGAGAAGCTTCACGCCGAGCTATTGGTTCTCAAAGGAAGGGACACAAATGAAGCACCCAG

CAACCTCAAGAAGGAAATTGAGAGGTCATATTGGAGCAATGGGAGCGAGAACAGTTCCGATATCAACTTAGATATTTCGA

GAACATCAGTTATGAACAGCCCTGTATCATCTTCACAGTTCAGCAACAAGCACCACTTCACCTCATCGAGGCCCTCG

>EgHD-Zip36

ATGGCCTTCTTTTCACCAAATTTCATGCTTCAAAGCCCGCACGATCAAGATCATGAACACCCTCATCACCAGCATCAGCA

CCAGATCCTCTCCTCTTGCACGCCTCAGGACTTCCATGGTGTTGCCTCCCTACTGGGCAAGAGATCCATGTCCTTCACGG

GCATTGACGTGGGCGACGACCCCAACATCAACAACGGCAACGTTAATGGGGAGGAAGATCTGTCCGAAGATGATGGGTCG

CAGCCAGGGGGAGAGAAGAAGAGGAGGCTCAACATGGAACAGGTGAAGACATTGGAGAAGAACTTTGAGCTTGGCAACAA

GCTTGAGCCCGAAAGGAAAATGCAGCTCGCGAGAGCACTTAATCTGCAACCCAGACAGATCGCCATTTGGTTCCAGAACA

GGAGGGCGAGGTGGAAGACGAAGCAGCTGGAGAAGGACTATGATCTGCTCAAGAGACAGTTCGATGCCGTGAAGGCTGAC

AACGAAACCCTCCAAGCTCAGAACCAGAAGCTTCAGACTGAGATACTGGCACTCAAGAATACCAGAGAACCAGCCGAATC

CATCAACCTCAACAAAGAAACCGACCAGGGCTCTTGCAGCAACCGGAGCGAGAACAGCTCCGAGATCAGGCTCGACATGA

CGCGGACGCCGCCGGTTGAGAGCCCGGTCTCAGGCCACGCGCTGCCGGCAGCTGGCCGGCAGCTGTTCCCTGCCTCGATG

AGGCCTGCCGCCTCCGGAGGATCGGTGGCGCAGCTGTTCCAAAACCCTTCGAGGCCTGATCTTCCGATGATAGTCAAGGA

AGAAAGCAGCATCACCAACATGTTCTGTGGCATCGAGGATCACTCTGGGTTTTGGCCATGGTTGGAGCAACAGCATTATT

GA

>EgHD-Zip37

ATGTCCTTCTCAGGGGCCGACCACAACCAGAGATCGTGTGGGGGCGGCGACGACAACGACCTGTCGGATGATGGGTCGCA

GCTGCTTCTGGGGAAGAAGAGGCTGAGCTTGGAGCAAGTGAAGGCACTCGAGAAGAGCTTCGAGATCGGGAACAAGCTGG

AGCCCGAGAGGAAGATCCAGCTCGCGAGGGCGCTCGGCCTCCAGCCGAGGCAGATCGCCATCTGGTTCCAGAACTGGCGG

GCTCGCTGGAAGACCAAGCAGCTGGAGAGGGACTACGAGGTGTTGAAGAAGCAGTTTGAGGTTCACGCCCAGAATGAGAA

GCTTCACGCCGAGCTATTGGTTCTCAAAGGAAGGGACACAAATGAAGCACCCAGCAACCTCAAGAAGGAAATTGAGAGGT

CATATTGGAGCAATGGGAGCGAGAACAGTTCCAATATCAACTTAGATATTTCGAGAACATCAGTTATGAACAGCCCTGTA

TCATCTTCACAGTTCAGCAACAAGCACCACTTCACCTCGTGGAGGCCCTCGGTCATGGCTCAACAACTCCATGGCTCAAC

CAGATAG

>EgHD-Zip38

ATGGCTGATTCTGGAGGTAGTGGAGGTTCAGGTGAGGAGCATGAGCATAGGCAGGACTCTCACCCGAGTCCAAATCCGAG

CTCGAGCTCGAGGAAAGGGAAGGGGAAGTCGAAGACGTACCATCGTCACACCCCACACCAGACACAGAGGCTTGAAGCGA

ATTTCCGCACCCCTGATGAGAATCAACGGCGTCAACTGAGCAGAGAACTCGGGCTTGACCCTAAACAGATCAAGTTCTGG

TTTCAGAACAAGAGGACTCAGAGAAAGACTCAAAATGAACGAGCAGATAACACAGCTCTTCGGGCTGAGAACGAGAGGAT

ACACTGCGAAAACCTTGCGATCATGGAGGCGCTCAAGAGCGTTATTTGCCCAGCTTGTGGTGGTCCGCCCTTTGGCGAGG

AAGAGAGGCAGCGTAATTTGGAGAAACTCAAGATGGAAAATGTGCAATTGAAAGAAGAGCATGCGAAAGTATCCAAAATT

CTCGCCAAATATATCGGGAAACCGATCTCGCAAATTGATTTGCTTATGCCTGGCCCCAGATCATCTGCGGACGTGTCCAC

CGCCAGTGTCCCCAACCAAGGGTTTAGTGGTCACAACGTTGACTTCGTTGTGGCCCCTCCCAACCCTGCGCCTTGGAGCC

AATTAAAGGTTATACCGGAAACAGAAATGGCCGACACATTGGAAATTACTGCATATGCCATGGATGAGTTAATCAGGATT

GTGCGGATGAAAGAACCGTTGTGGATAAAGTCTCCTCTAGACGGAAGATACATACTCCATCGTGACACCTACGAGCAGAT

ATTTTCTCGAAACTACCGTTTTAGGGGCTCTGGGGCTCGCCTGGAGACATCCAAGGAATCAGTTCTGGTGACGATGGAAC

CAAGCAGGCTGGTTAACATATTCCTGGATGTGCGCAAATGGGCGGATATGTTCCATACAATTGTCACAAAAGCTTCTTCT

ATCCCAGTTCTTGAAGAAGGAACACAACTTAATCACAGAATTTACTTGCAGCTGATACATGCACGAATGCACGTGCTTTC

TCCGCTAGTGCCGGCTCGGGAAGTCTACTTTCTTCGCCGTTGCCAACAGATTGAGCTAGGCATGTGGGTGATAGTGGATG

TGTCCTACAATTACATGACAGGTGATGCGTGCCCTTACAATACCTGGAGGCTTCCTTCTGGCTGCGTGATTCAGGACATG

GGTAATGGTTGCTCCAAGGTCACTTGGGTGGAACACGTAGAAGTGGACGACAAAAACCAATCTCATAAGCTTTTTAGGGA

TCTAGTGTGTGGCGGCCTCACGTACGGAGCAGAAAGATGGGCGATTACGCTCCAAAGAATGTGCGAGAGATTTGCTTACT

CCATGCTGGATGATGCGCCAACGCACGATGCTGTGGAAGTTGGTATTACAACTTCACCGGTCATTGTTGTTCCTGAAGGT

CGGAGAAATGTGATGAAGCTTGCTCATAGGATGGTGAAGAATTTCAGTGCGGGCTTGAGCATGTCCGGCAAATTAGATTT

CCCTCACCTGTCTGAGGTTAACAACAGTGGGGTCCGTGTCTGTGTACGAAAGAGCGAAGAACCAGGACAGCCCAGCGGCA

CGATTGTTAGCGCGGCCACATCCCTATGGCTTCCTGTCGCACCTCAAGCGGTCTTCAATTTCTTCAGAAACGAAAATACT

CGTGCTCAGTGGGATGTACTCTGCAATGGAAATCCAGTCCAAGAGATAGCACGCATACCGAGTGGCACTCATCATGGAAA

CTCAATCTCAATTATTCAGACTTCTCTGCATGCCGAGAACATGTTGATGCTTCAAGAGAGTTGCATAGACCCTTTGACGT

GTATGGTGGTTTATGCCCCTATCGATTCAACAGCGATGACCATCGCGTTGAGCGGCGAGGACACTTCCACGGTGCAAATT

CTTCCATCTGGCTTCACGATCTCCAGCGATGGCCGGGCCAATCCAGGTTCAAAACCTTCGACCAGCACAAGCTCCAGCAA

GCCAGCAGGTACACTTCTCACTGTGGCATTCCAGATATTGGTTTCCAGCCACTCAGGACCAGAGCAGCTCAACGTGGAAT

CCGTGGCGACCGTGAACACTCTCATTAGTGCGACCGTTCAGAAAGTTAAGGCTGCTCTAAATTGCTCTGCCCCGGAATGA

>EgHD-Zip39

ATGAAGATCTGGAAGTCCCACCAAACAAACTCCAAAACAAGGCCATTCTTCGGTGGAGTCGGCGTCAACCGAGACATACT

GGTGGTGGATTTGGATGATGATGATCATGTCTCATCACCTAACAGCACCGTGTCGAGCATAAGTGGGAAGAGAAGTGAGA

GAGACCCGCATGGAGAAAATGAAGTTGAGGCTGAGAGAACATCGTGCTCTTGTGAGAGCATCGAAGAAGATGGCAGTGGA

GGAGATGGAGCGAGGAAGAAGCTCAGGCTGTCTAGAGAACAGTCATTGGTGCTAGAGGAGACATTCAAAGAGCATAACAC

TCTTAACCGTGTAGAGGTCTGGTTTCAGAACAGGCGTGCTAGGACTAAGACGAAGCAAACTGAGGTGGACTGTGAGTACC

TTAAGAGATGCTGCAACAATCTTACACAGGAAAACAAGAGGCTGCAGAAGGAAGTGCAGGAGCTCAGAGCACTGAAGCTC

TCCCCACAGCTCTACATGCATTTGAACCCTCCCACCACGCTCACCATGTGCCCTTCATGTCAGCGAGTGTCTGTTCCTTC

AGCATCATTGTCGCCTCCATCTTCATCTTCAGTCACTGCCTCAGTGATTGGCCCCATAGGGCCAGTCCATAATCCTTTTA

GTCAGTCTGGACCATCCATCAACCCTTGGGCAAGGCTACAGATGCAACAAGGACTGAACAATTTGCACTCTTAA

>EgHD-Zip40

ATGGGCGAGAGAGATGATCTGGGCTTGAGCTTGAGCCTGAGCTTCCCTCAGGGTCACCTGCATCAGCAGCAGCAGCAGCA

GCAGCGGCAGTCCCTGCAGCTGAACCTCATGCCCTCCTTGGTCCCGTCCTCTGCTTCGTCTGCTCAATCTGGGTTCAATC

TTCAGAAGCGCTCCTGCAACGACGCCTTCCCTTCTTCTTCAGATCGGAACTCCGAGGCGCGATCGTTCCTCCGGGGGATC

GACGTGAACAGAGAGCCGTCGGCGGGGGCGGCGGCGGACTACGGCGAGGACGAGGCCGGGGTGTCGTCGCCGAACAGCAC

GGCGTCGACGGTGAGCGGGAAGCGGAGCGAGAGGGATCACCAGAGCCAGACCAACGGCGACGACCTCGACAACGAGAGGG

CGTCCTCCCGCGGCGGCGGCAGCGACGAGGAGGACGGCGACATGTCGAGGAAGAAGCTCCGGCTGTCGAAGGACCAGTCC

GCCGTCCTCGAGGAGAGCTTCAAAGAGCACAACACCCTCAATCCTTGGGTGCGTTTCATGCAGAAGCAAAAGCTGGCACT

GGCGAAGCAGCTGGGGCTGCGGCCCAGACAAGTGGAGGTCTGGTTCCAGAACAGGCGAGCCAGGACGAAGCTGAAGCAGA

CGGAGGTGGATTGCGAGTACCTGAAGCGGTGCTGCGAGAGCCTGACGGAGGAGAACCGGCGGCTGCAGAAGGAGGTGCAG

GAGCTGCGGGCGCTCAAGCTCTCCCCGCAGTTCTACATGCACCTCTCCCCTCCCACCACCCTCACCATGTGCCCCTCCTG

TGAGCGCGTCGCCGCCCCGTCTCCCCCCTCCGCCGTCGGCCGCCCCCTCGCCGCCGTCCCGGCCCACCCCCGCCCCGTGC

CCCTCATCAACCCATGGGCCCCTGCGGCCGCCCCACTGGCCCACGCCCCGTTCGACGCCCTCCGCTCCTGTTCGTGA

b) HD-Zip protein sequences used in current study.

>EgHD-Zip1

MDFGGGSGGDHDGSDHQKRKKRYHRHTAHQIQRLEAMFRECPHPDEKQRMELSRELGLAPRQIKFWFQNRRTQMKAQHER

ADNCALRAENDRIRCENIVIREALRNVICPSCGTVPSGEDSYFDEQKLRMENAHLKEELDRVSSIAAKYIGRPMSQLPPV

QPIHIASLDLTMSSLGAHGLAGPSLDLDLLPGSSSSVPHLPFQAVVFSDMDKSLMADIAANALDEFLRLAQTDEPLWMKS

TTDGRDNLNLESYERMFPRASSHLKNPDVRVEASRESRVVMMNGLALIDMFMNSNKWAELFPAIVSAAKTIEVISPGLLT

SQNGSLQLMYEELQVLSPLIPTREFYFLRYCQQIEPGLWAIVDVSFDLSRYDQFAFQSRSQRLPSGCLIQDLPNGYSKVT

WVEHVEIEDKTPVHRLYRDLIYSGLAFGAERWIATLERMCERIACLMVTGSSTRDTRGVIPSPDGKRSMMKLAQRMVNNF

CSSISTSSSRQQRWTTLSGSSEVGVRVALHKSTDPGQPNGVVLSAATTIWLPLSPQNVFNFFKDERTRAQWDVLSNGNAV

QEVAHLANGSHPGNCISVLRAFNTSQNNMLILQESCIDSSGSLIVYCPVDLPAINLAMSGEDPSYIPLLPSGFTISPDGG

PDPGDGASSSSAAAAGQGSTSRSAGSLMTVAFQILVSSLPSAKLNLESVTTVNNLISTTVHQIKAALNCPGS

>EgHD-Zip2

MEKSENCTAGNMKKKKGKSKNMKKGFSDEQIRLLETMFESEAKPDPRRKMELARELGLEPRQVAIWFQNRRARWKSKQVE

QEYRVLRASYEKLLAEFECLKTEKQDLENELQKLSGMLDKNHGGGRICREASNLEDEDTDCKNEANPFGPHESFDQSLIV

ASPDDQLNETEKSSEDVRESRDEENCSVALPEKWSNFGPSDILDYSCSNAAPWWNFWT

>EgHD-Zip3

MEGQSEIDRFGEPFEGSFLGRLKDEGSGSDNQLDGASGDDDQDDAVDEEAGVLPRKRKKYHRHSPHQIQELEAFFKECPH

PDEKQRLELSRRLGLESKQIKFWFQNRRTQMKTQMERHENIMLRDDNNRLRNENDAMRSKLANPICGNCGGTAIFNGAGM

VSYEEHQLRVENARLKEEVARVRALTDKFLGKQPPTALLPSSPSRSNRGPEFSAQGHVPNVSTTATAALPMGISSLDGMS

NASPVSPYARPAVAMGRNQLSTEKSAYVEVAIASMNELIKMAEPNSCLWLRTLDGAKEVLNHEEYSRLFTPLASARHSEV

VTEASRETSIVPINSLAVIEILMDVNRWVETFPCIVARSSTIEVMSSGIRGNRNGALQVMHAEFQVLSPLVPLRQVRFLR

FCKQHGEGMWAVVDVSVGMLQDSSDPCGSANCRRLPSGCVLQDMPNGCSKVTWIEHSEYDGTAVHPLYHSFLISGMGFSA

QRWIASLQRHCECLLTFMSSTYPNEDGSGMNPSGRRNMLKLAQRMTDNFCSGVCASPLHNWEVLHLGNMNGDVRVTTRKN

AGRPGEPPGIVLSAATSIWMPIAHETVFNFVRNEQLRSEWDILSHGGPMQEIVHVAKGPRRDNCVSLLRANALSASDTSM

VILQETWTDASGSLIAYAPVDITVIESVMSGGDPNSVALLPSGFALLPDSPSHIDGLKNPDESLIGTADSRGCLMTIGFQ

ILVSSTPAAKLTAESVETVHNLLSRTIQRIKTSLQMS

>EgHD-Zip4

MGVVDMSNNNPPTSRTKDFFASPALSLSLAGIFRDAGAAAAASASMDVEEGDEGSGGGGGRREDTVEISSENSGPARSRS

DDEFDPDGDNDEDGGDGDKSKKKKRKKYHRHTAEQIREMEALFKESPHPDEKQRQQLSKQLGLAPRQVKFWFQNRRTQLK

AIQERHENSLLKTEMEKLRDENKAMRDTIQKSCCPNCGSATTSRDTALTTQEQQLRIENARLKAEVEKLRTALGKYTPGT

ASPSCSAGNDQENRSSLDFYTGIFGLDKSKIMELVNQAMEELKKMATAGEPLWIRSVETGREILNYDEYVKEFKVEAPSE

GRPKRSIEASRETGVVFVDLPRLVQSFMDVNQWKEMFPCMISKAATVDVVCSGEGPNRNGAVQLMFAELQMLTPMVPTRE

VYFIRYCKQLSAEQWALVDVSIEKVEDNIDASLVKCRKRPSGCIIEDKSNGHCKVIWVEHLECQKTTVHPMYRTIVNSGL

AFGARHWMTTLQVQCERLVFFMATNVPTKDSNGVATLAGRKSILRLAQRLTQSFCQAIGASSYHSWTKVPTKTGEDIRVA

SRKNLNDPGEPLGVILCAVSSVLLPVSPHVLFDFLRDESRRSEWDIMASGSPVQSIANLAKGQDRGNAVNIQTMKNKDNS

MWVLQDCCTNAYESMVVYAPVDITGMQAVMTGCDSSNIAALPSGFSILPDGIESRPLVISSRHEEKSSEGGSLLTIAFQI

LTNTSPTAKLTVESVESVNTLISCTLRNIRTSLQCEDG

>EgHD-Zip5

MSFGGFLDTNSGGGGGGGGGARVVAADIPYAGKNDMAPSGAIGQPRLLSPSLSKSMFNNSPGLSLALQTGIENQQMGESY

EQMSVSNHHLRRSREEEHESRSGSDNLDGGGASGDDQDASADNPPRKKRYHRHTPQQIQELEALFKECPHPDEKQRLELS

KRLCLETRQVKFWFQNRRTQMKTQLERHENSLLRQENDKLRAENMSLRDAMREPICSNCGGPAVIGEISLEEQHLRIENA

RLKDELDRVCALAGKFLGRPVPSLAASIGPLMPNSSLELGVGTNNGFVGSLGPVAATTLPLGPDFGGGISGNSAMAVVNP

SRPPGAGGGMVSIERSILLELALAAMDELVKMAQTNEPLWIRSLEGGREMLNRDEYMRTFSPCIGMKPNGFVTEASRETG

MVIINSLALVETLMDSNRWAEMFPCMIARTSTADVICSGMGGTRNGALQLMQAELQVLSPLVPVREVTFLRFCKQHAEGV

WAVVDVSVDGIRETPGGGGGAASFPSCCRRLPSGCVVQDMPNGYSKVTWVEHAEYDENHIHQLYRPLISSGMGFGSQRWV

ATLQRQCQCLAILMSSTVSSRDHTAITPSGRRSMLKLAQRMTANFCAGVCASTVHKWNKLSGGNVDEDVRVMTRKSVDDP

GEPPGIVLSAATSVWLPVSPQRLFDFLRDEQLRSEWDILSNGGPMQEMAHIAKGQDHGNCVSLLRASAMNANQSSMLILQ

ETCIDAAGSLVVYAPVDIPAMHVVMNGGDSAYVALLPSGFAIVPDGPDGPGSHGGGAAPNGPAASNGGPPRVGGSLLTVA

FQILVNSLPTAKLTVESVETVNNLISCTIQKIKAALQCES

>EgHD-Zip6

MPAGIMIPARNMSSSPMIGSNGNASGYGSSSGLTLGQPNMMESLHHHQLHHPLDMAHQTPSESELARLRDEEFEISTKSG

SENQEGQSGDDMQDLQRPNKKKRYHRHTQHQIQEMEAFFKECPHPDDKQRKELSRQLGLEPLQVKFWFQNKRTQMKTQHE

RGENTQLRAENDKLRADNMRYREALSNATCPNCGGPTAIGEMSFDEHHLRLENARLREEIDRISAIAAKYVGKPVVNYPL

LSPSAPPRPLDLGVGGFGGQSSVGGEIYGAGDLLKSIGAPTEADKPMIIELAVAAMEELIRMAQMGEPLWSESLDGNSSS

VLNEDEYMKTFPRGIGPKPAGFNTEASRESAVVIMDHINLVEILMDVNQWSTVFSSIVSRAATLEVLSTGVAGNYNGALQ

VMTAEFQVPTPLVPTREVYFVRYCKQHGDGAWAVVDVSLDNVRPSPIVRCRRRPSGCVIQEMPNGYSKVTWVENVEVDDR

GVHSLYKQLVNSGHALGAKRWVATLDRQCERLASAMATNIPNTDVGVITSQEGRKSMLKLAERMVISFCAGVSASTAHTW

TTLSGTGADDVRVMTRKSIDDPGRPPGIVLSAATSFWLPVQPNRVFDFLRDENSRSEWDILSNGGVVQEMAHIANGRDTG

NCVSLLRVNSANSSQSNMLILQESCTDSAGSFVIYAPVDIVAMNVVLNGGDPDYVALLPSGFAILPDGSAMQGGNDQQGV

GSGGGSLLTVAFQILVDSVPTAKLSLGSVATVNNLIACTVERIRASLSCESA

>EgHD-Zip7

MEAGRFLFDPPALQGNILFLDKGSRSMMGMEESPKRRRFFCSPDELFDEEYYDEQMPEKKRRLTPEQVLLLEKSFEEENK

LEPERKTQLAKKLGLQPRQVAVWFQNRRARWKTKQLERDYDYLKSSYDSLLSDYDSILKENEKLKLEVYSLTEKLQGKEV

DGAPMTGPSEPAPLEEADVQAVQFSAKVEDRLSTRSGGSAVIDEEGPQLVDSGNSYLLCENYPGCVAQSEDDGSDDGRSY

FPGVFAAATEQPHHEEEEEPMDWWVWS

>EgHD-Zip8

MFGDCQVMSSMGGTGISAESLFSSPIRNPNFDFMSSLPFNAFAPLIPKEEEINGMLGGSSNSKEEMVDSGGSGSEHFLEE

KSSANELLDLDGGDQDQPANKKKRYHRHTARQIQEMEALFKECPHPDDKQRMRLSQELGLKPRQVKFWFQNRRTQMKAQQ

DRADNAILRAENDNLKNENYRLQAALRNIVCPNCGGPGMLGEMGFDEQQLRIENARLKEELERVCCLASRYSGQQIQSIP

VLPPLSLLPPSLDLEMTIYPPRHFPEAMSSCSDIMPMPLLPQDTPHHLSESTGLILMEDEKALALELAMSSMEELSKMCL

ASEPLWVQSSESGKVVLNFEEHARLFPWPLNNNLKQNSDKLRKEASRDSAVVIMNSITLVDAFMDAGKWMDLFPTIVSRA

KNIQVIASGVAGHARGSLNLMYAELQVLSPMVPTRETYFLRYCQQNVDEGSWAIVDFPMDSLHNNLASPPSMPRYIRQPS

GCVIQDMPNGYSRVTWVEHAEIEEKVGHPLFSQFVKCGMAFGAHRWLAVLQRQCERARKNLMRLAQRMIRTFSTNISTSG

GQSWTALSDSSDDTIRITTRKITEPGQPNGVVLAAVSTTWLPYPHHHVFELLKDERRRSQLDVLSNGNSLQEVAHIANGS

HPGNCISLLRINVASNSSQHVELMLQESCTDASGSLVVYSTIDVDSIQLAMNGEDPSHLPLLPIGFSIVPFGSGSPGSDS

GANDNLGLDACPQGEGCLLTVGLQVLASTIPTAKLSLSSVNAINNHLCMAVHQITAALASSSSNGNSSPAPCN

>EgHD-Zip9

MNDLSEDDMALISELYPGVYMRPSPPPQEAKPPRRRRKKSRGLENPGEEGGGTGGEGRGKRKLTAEQVELLEQNFGDEHK

LESERKDRLAAELGLDPRQVAVWFQNRRARWKSKKLEVEYAKLKSVHETVVVEKCRFESEVLKLKEQLSEAEMEIKRLSE

KADLGLSGNSPCSSVTADAATDDP

>EgHD-Zip10

MMDIFQQNLMEGGGGPPLHPLDMVQNPSESELARLREDDFDSTKSGSDNHEEPPSGDDLQDERRKKKRYHRHTQHQIQEM

EAFFKECPHPDDKQRKELGRQLGLEPLQVKFWFQNKRTQMKTQHERHENTQLRSENEKLRADNMRYREALTNASCPNCGG

PTAIGEMSFDEHHLRLENARLREEIDRISAIAAKYVGKPIANYPLLPSSVPPRPLELGFGSFSGQSGVGGDIYGSGDLFR

SIPAPTEAEKPMIIELAVAAMEELIRMAQEGEPMWIAGPDGSSSVLNEDEYIRAFPRGIVTNPTGFKREASRQTGVIIMN

HINLVEILMDVNQWSTIFSSIVSRAMTLEVLSTGVAGNYNGALQVVTAEFQVPSPLVPTREVYFVRYCKQHRDGTWAVVD

VSLDTLRPNPAVRCRRKPSGCLIQEMPNGYSKVTWLEHVEVDDRGVHNLYKQLVGSGHAFGAKRWVATLDRQCERLASAM

ATHIPSGDVGVITSPEGRKSMMKLAERMVISFCAGVSASTAQTWTTLSGTGADDVRVMTRKSVDDPGRPPGIVLCAATSF

WLPVSPKQVFDFLRDENSRSEWDILSNGGIVQEMGHIANGRDTGNCVSLLRVNSANSSQSNMLILQESCTDATGSFVVYA

PVDIVAMNVVLNGGDPDYVALLPSGFSILPDGSQGSGEVASGGSLLTVAFQILVDSVPTAKLSLGSVATVNNLIACTVER

IRASLW

>EgHD-Zip11

MGIEEATKRQSIFSYPEDLYNEEYYDDQAPEKKRRLTPEQVHLLEKSFEVENKLEPEKKTQLAKKLGMQPRQVAVWFQNR

RARWKTKQLERDYDVLKSSYDSLLLSYDSIKKENEKLKSEVNFLNEKLSAKDVAGAPASGQSSHALQVEETRDSPLGFVV

KVEDRLSSGSGGSAVVDEDGPQLVDSGHSYFHCNDYPGSLVAVNGLQSEDDGSDDSRGYCSEIFAAAEEPHQEGGVPMGW

WAWP

>EgHD-Zip12

MFQPNMFDSHHHLLDITGARSSESEVLKMREIEDFETKSGTETMEPDPSGDDVQDPNNDDNSNNNGQRQKRKRYHRHTQR

QIQELEAFFKESPHPDDKQRKELSRELGLEPLQVKFWFQNKRTQMKAQHERHENAILKAENEKLRAENMRYREALSTATC

PNCGGPAALGEMSFDEQHLRIENARLKEEIDRISVIASKYVGKPLASNYPHLPPHMSSRSPDQFPAQSGLVGEMYGGIDL

RRSVSMPSEADKPLIVELAVAAMEELIRMAQGGEPLWIPAGSGQPSEILNEDEYFRIFPRGIGPKPLGFKSEASRESAVV

IMNHINLVEILMDEHQWSGVFCGIVSRAMTIEVLSTGVAGNYNGALQVMTAEFQVPSPLVPSRENYFVRYCKQHGDGTWA

VVDVSLDNIRGNPILRSRRRPSGCLIQELPNGYSKVTWVEHVEVDDRAVHSIYRPLVNCGLAFGAKRWVATLDRQCERLA

SSMAINIPSGDLLITSPEGRRSMLKLAERMVLSFCSGVGASTAHAWTTLSAAGSDNNVRVMTRKSMDEPGRPPGIVLSAA

TSFWLPVPPKRVFDFLRDENSRNEWDILSNGGQVQEMAHIANGRDPGNSVSLLRVNNANSSQSNMLILQESCTDSVGAYV

IYAPVDIVAMNVVLNGGDPDYVALLPSGFAILPDGPEFGGGGGILEIGSGGSLLTVAFQILVDTVPSAKLSLGSVATVNN

LIKCTVERIKASVSCDNP

>EgHD-Zip13

MFSTGEYSAAAFEGMDSLPSPRKMKNQLVNRRRFSDEQIRSLESIFESESRLEPRKKLQLARELGLQPRQVAIWFQNKRA

RWKSKQLEREFAILRANYNSLYSQFESLKKEKHSLVTQIEKLNQLVEKPQGEGQSCGHDLATNSIDRESDNGVPKHEDSQ

PVFPDKLTRLMGIPCEDDYFGLEEEQSLLNMAEPDPEPAPVDSSLTSPEPWGSLETDDLLNQSSGSSQWWDFWS

>EgHD-Zip14

MAGGASASASAITTLLQNQRVPPPPPSDAFFLSGSSPFLGSRAMVSFEDAHRGNRLNRPFFRSIDPDENGEDDLEDYFHQ

PEKKRRLTVEQVQFLEKSFEVENKLEPDRKIQLAKDLGLQPRQVAIWFQNRRARWKTKQLEKDYETLQASFNTLKSDYDT

LIKERNDLKAEVLNLTDKLLHKGNEKESSESSSKSSQGLFQNPIADSVSEDEVSRVPIPTWPEDICSVKSDMFDSESPHY

TDAAHSSLLEPGDSSYAFEPDHSDLSQDEEDNLSKSLLSTRNYPKLENSDYAILPPNSCNFGFHAEDPAFWPWSY

>EgHD-Zip15

MDWHGDLRSFSNVSFPESSIGFLNPSFAQYPGMELKHHHHHHPPPLSSSMAEAHNDVVVAASMDKKKKLTSEQLEMLERS

FQEEIKLDPDRKMRLSRELGLQPRQITIWFQNRRARWKTKQLERLYDALKLEFDAVSREKLRLQEEVSRLKAMVSDIQAA

KKKQVSTGYTDISGDETVESARYSGDIRHCSDLTRGAGGGGGVTPTPSTTQHHPLAGSSYQLFTLQDYESNSYWGVLPSS

YP

>EgHD-Zip16

MGDLGDFPGSSSSLELTISVPGFSSSPPLPSSVRDLDINQVPSGGEEEWMTMSGGMEDEEESSHGGPPRKKLRLTKEQSR

LLEESFRQNHTLNPKQKEALAAQLKLRPRQVEVWFQNRRARSKLKQTEMECEYLKRWFGSLTEQNRRLQREVEELRALKV

GPPTVISPHTCQPLPASTLSMCPRCERVTSTAPPPTSTSAPSAATRAPSKASQLSPPSVHSRQPSAAS

>EgHD-Zip17

TNSKTRPFFGGVGVNRDILVVDLDDDNHVSSPNSTVSSISGKRSERDPHGENEVEAERTSCSCESIEEDGSGGDGARKKL

RLSGEQSLVLEETFKEHNTLNPKQKLALAKQLNLSPRQVEVWFQNRRARSKMKQTKVDCEYLKRCCDNLTQENKRLQKEV

QELRALKLSPQLYMHMNPPTTLTMCPSCQRVSVPSASLSPPSSSSVTASVIGPIGPVHNPFSQSGPSINSWARLQMQQGL

NNLHS

>EgHD-Zip18

MAGGGGCEGSAIASLLQNQRVSPSSDAFFFYGSSFSVGSRSMVSFEDASGANVSKNPFFQAFDPHEIGEEELDEYLHQPE

KKRRLTTEQVHFLEKNFELENKLEPERKIQLAKDLGLQPRQVAIWFQNRRARWKTKHLEKEYEDLQASYNSLKADCDGLL

KENDKLKTEVLVLTDKLLIKARGTQNSKLSNASSPGPPENPVACSKGEEERISTVPEDVCPGKSEISDSDSPNGGYSPLR

EHGDSSYAFEPDLSDSSQDEDYTRNENLQHLCVFPKLEETRDDYPILSTSSCHFGFPPEDQASWPWPY

>EgHD-Zip19

MATSCKEGKLGHSNSSNSLDNGKYVRYTPEQVEALERLYHECPKPSSLRRQQLIRECPILSNIEPKQIKVWFQNRRCREK

QRKEASRLQAVNRKLTAMNKLLMEENDRLQKQVSQLVYENGYFRQHTQNTTLATKDTSCESVVTSGQHQLTSQHPPRDAS

PAGLLSIAEETLAEFLSKATGTAVEWVQMPGMKPGPDSIGIVAISHGCAGVAARACGLVGLEPTRVAEILKDRPSWFRDC

RAVDVLNVLPTANGGTIELLYMQLYAPTTLAPARDFWLLRYTSVLEDGSLVVCERSLKNTQNGPSMPPVQPFVRAEMLPS

GYLVRPCEGGGSIIRIVDHLDLEPWSVPEVLRPLYESSTMLAQKTTMAALRQLRQIAQEVSQPNVSGWGRRPAALRALSQ

RLSRGFNEALNGFTDEGWSIMGNDGIDDVTILVNSSPDKLMGLNLSFSNGFPAVSNAVLCARASMLLQNVPPAVLLRFLR

EHRSEWADNSIDAYSAAAVKVGSCALPGSRIGSFGGQVILPLAHTIEHEEFLEVIKLEGMGHSPEDALMPRDIFFLQMCS

GVDENAVGTFAELIFAPIDASFADDAPLLPSGFRIIPLDSVKEASSPNRTLDLASSLEIGPAGNRSFNDINANSGCTRSV

MTIAFEFAFESHMQEHVASMARQYVRSIISSVQRVALALSPSNLGSHAGLRTPLGTPEAQTLARWICHSYRCYLGVDLLK

SSNEGSELILKNLWHHSDAIMCCSLKALPVFTFANQAGLDMLETTLVALQDITLEKIFDDHGRKTLCSEFPQIMQQGFAC

LQGGICLSSMGRPVSYERAVAWKVMNEEENAHCICFMFINWSFV

>EgHD-Zip20

MAVTSACKDKMGIDNGKYVRYTPEQVEALERLYHECPKPSSLRRQQLIRECPILSNIEPKQIKVWFQNRRCREKQRKEAS

RLQAVNRKLTAMNKLLMEENDRLQKQVSQLVYENSYFRQQTQNATLATTDTSCESVVTSGQHHLTPQHPPRDASPAGLLS

IAEETLTEFLSKATGTAVEWVQLPGMKPGPDSIGIIAISHGCTGVAARACGLVGLEPSRVAEILKDRPSWYRDCRAVDVA

NVLSSGNGGTVELLYMQLYAPTTLAPARDFWLLRYTSVMEDGSLVVCERSINNTQNGPSMPPVQHFVRAEMLPSGYLIRP

CEGGGSIIHIVDHMDLEPWSVPEVLRPLYESSTLLAQRTTMAALRNLRQISQEVSQPNVTGWGRRPAALRALAIYLDRGF

NEAVNGFMDDGWSMLESDGVDDVTLLINSSPAKMAGVNISYASGFPSMTSAVLCAKASMLLQNVPPAILLRFLREHRSEW

ADSSIDAYSAAAIKASPCNMPGTRIGGFGSQVILPLAHTIEHEEFMEVVKLENMGHYRDDMIMPSDIFLLQLCNGVDENA

VGTCAELLFAPIDASFSDDAPIIPSGFRIIPLDPGSDASSPNRTLDLASALDVGPTGNKAVGDNSGHSGNTKSVMTIAFQ

FAFELHLQENVASMARQYLRSIIASVQRVALALSPTNLGCHASLRPPPGSPEAHTLARWICQSYRSFLGVALLKNEAADS

LLKNLWHHSDAILCCSLRAAPVFTFANQAGLDMLETTLIALQDITLEKIFDDNGRKTLCSEFPQIMQQGFMCLQGGICLS

SMGRPISYERAVAWKVLNEEETAHCICFMFVNWSFV

>EgHD-Zip21

MRITMDNDHQLEEHMALISELYPGVYTQIVPPVKESKPRRRRKKSKGEGGAAGPRKRKLSEEQVNLLEQNFGSEHKLESE

RKDRLASELGLDPRQVAVWFQNRRARWKNKKLEEEYAKMKSAHEGTVIEKCHLESEVLKLKDQLIESEKEIQRLLERADG

PAASSNSPSSSMSMDASMDRRFPGEFAYEDVFYMQENVYNVYGMDWMNLFDSRQ

>EgHD-Zip22

MGIDDLCNTGLVLSLGLETPFKIEAQRQAKQRLNFEPSLTLCLSGTTKATRDEQPPADHLYRQASPHSHNSLSAVSSFSS

PRVKRERDLSSEEAEVETRVSSKASDEDDDGANARKKLRLTKEQSALLEESFKQHSTLNPKQKQALARQLNLRPRQVEVW

FQNRRARTKLKQTEVDCEFLKKCCETLTDENRRLQKELQELKALKLAQPFYMHMPAATLTMCPSCERIGAGPSVDGAAPT

KGPFSMTTKSHLYSHHFTNPSAAC

>EgHD-Zip23

MALAMHRECSSKQMDASKYVRYTPEQVEALERVYNECPKPSSLRRQQLIRECPILCNIEPKQIKVWFQNRRCREKQRKEA

YRLQTVNRKLNAMNKLLMEENDRLQKQVSQLVYENGYMRQQLHSQASTATTDNSCESVVMSGQHQQQHNPTPQHPEKDAS

SPAGLLAIAAETLTQFLAKATGTAVDWVQMIGMKPGPDSIGIVAVSRNCNGVAARVCGLVSLEPMKVAEILKDRPSWFRD

CRCLEVLNVIPAGNGGTIELVYMQTYAPTTLAAARDFWTVRYTTTLEDGSLVICERSLTTSTGGPSGPSSSSFVRAEVLP

SGYLIRPCEGGGAIIHIVDHVDLDAWSVPEVLRPLYESSKILAQKMTVAALRHIRQIAQESSGEIQYGGSRQPAVLRTFS

QKLCRGFNDAVNGFVDDGWSVLSSDGVEDVTIAVNSSPNKFLGSQYNATIFPNFGRGVLCAKASMLLQNVPPAVLVRFLR

EHRSEWADHGIDAYSAASLKTSSYAIPCVRPGGFPSSHVILPLAHTVEQEEFLEVVRLEGHAFSPEDIPLAHDMYLLQLC

SGVDENAVGACAQLVFAPIDESFADDAPLLPSGFRVIQLDPKTDGPAPTRTLDLASTLEVGSGGARPTCEADASTYNLRS

VLTIAFQFVFENHLRDTVAIMARQYVRSVVGSVQRVAMAIAPSRLGGHLGPKSLSGSPEALTLARWICRSYRICAGAELL

RGDSQAGDAVLKEFWHHSDAIMCCSVNTNVASPVFTFANQAGLDMLETTLVALQDIMLEKVLDEGGRKVLSSEFPKIMQQ

GIAYLPAGVCISSMGRPVAYEQAVAWKVLNDDDSHHCLAFMFMNWSFV

>EgHD-Zip24

MMVEREDLGLSLSLSFSDSSRPSQLGASPFGFNLYKPSHRDCETFASLDRISEADARPSLRGIDVNRPPPSAADCEEQEE

AGVSSPNSTISSVSGKRGEREMVSGGEDNEAERDCSRGGSDEEDGENSRKKLRLSKDQSAVLEESFREHNTLNPKQKLAL

AKQLGLRPRQVEVWFQNRRARTKLKQTEIDCEFLKRCCENLTEENRRLQKEVQELRALKLSPQFYMHMPPPTTLTVCPNC

ERVGAAAPPLPSAGGGGRPAHHREPVPMIPWAARPGPVSHGALRPRT

>EgHD-Zip25

MGDIEEACNTGLFLGLSVGGSTTKRKEEKKKNKQNNDAPSTVGLGLSFSINCSSSKVEADDHPWDSINNAEYPARSSFHN

SFVDQSPDDHISPPRTSNDDGGDFNGGRKKLRLTREQSALLEESFKLHTTLTPAQKQSLADQLKLKPRQVEVWFQNRRAR

SKLKQTEVDCQFLKKCCETLSDENRRLKKELQELRSSSSPYVQGPGTAAVMLCPSCEELTRGNSEATRKENCDI

>EgHD-Zip26

MAGEEPYSADTNSDTFADEETLIPSSSEALESAWVPTSSTAHHGSKSVVNFEDVCGGGDTNTAPRPYLRQIDLKEEAVEE

DYGDGNFQPPGKKRRLSADQVHFLERHFEVENKLEPERKIQLAKDLGLQPRQVAIWFQNRRARYKTKQLEKDYDSLKARF

ESLKADHDKLIKEKENLKGEVLSLRDKLRSRAKGSEEGSLEADDSLRGATPKPTTPSTLDDVSYVDATILKQEDVSSAKS

DVFDSDSLLEPANSSHVFEPDCSDFSQDEDDSFGKSLLPLDFLPRFDDGYAFQQNPPAISCNFAFPAEENPFWSWSY

>EgHD-Zip27

MGEQDDGLGLSLSLGCGKSALSLNLNLVEAPVQSRQSPGQKCSWNEIFHSPETNSKTRPLFGGVGVNRDILVVDLDDDNH

VSSPNSTVSSISGKRSERDPHGDNEVEAERTSCSCESIEEDGSGGDGARKKLRLSREQSLVLEETFKEHNTLNPKQKLAL

AKQLNLSPRQVEVWFQNRRARTKTKQTEVDCEYLKRCCDNLTQENKRLQKEVQELRALKLSPQLYMHMNPPTTLTMCPSC

QRVSVPSSSLSPPSSSSVTASVIGPVGPVHNPFSQSGPSINPWARLQMQQGLNNLHS

>EgHD-Zip28

MCPIDSGRSFDTSLSLGLGCYGDPEDHEIKIKKPLAKLSGNSTCLTIGLPGGEACGLGSASGDEVRNIPSRSASSFSNSS

SAKREKAEQGEEEAVERGTGSPRATINIEDEDEFSPRKKLRLSKAQSSILEESFKAHTTLNTKQKHDLANRLNLRPRQVE

VWFQNRRARTKLKQTEVECEMLKKCCETLKEENRRLKKELQELKSLKPTASVYRQIPAAALPLCPSCERIAHPEFPFSTE

SRLWPAHPSAAC

>EgHD-Zip29

MKRLGSSDSLGALMSICPPSEELQHSPRNGNPIYHSRDLQSMLELGLDEEGCVEDQSAGGGGHVGGEKKRRLSIDQVKAL

EKNFEVENKLEPERKVKLAQELGLQPRQVAVWFQNRRARWKTKQLERDYGVLKSSYEALKLSYDALKHDNEALHKEIKEL

KSKLREEDDNPESNLSVKEEVIIPSHDVSDKIRAADDGDDDTKRSPPPPITAPPRELSFNNGGLKDGSSDSDSSAIVNEE

NAATSSSSPNPAVQSHGGFLKFMGSSSSSASPPPPPPASFGGCFSFQFQRAYQPQPQPPHHHHHHSPYVKMEEHNFLGGE

EDCNFFSDEQAPTLHWYCPDQWN

>EgHD-Zip30

SISGKRSERDPHGENEVEAERTSCSCESIEEDGSRGDGARKKLRLSREQSLVLEETFKEHNTLNPKQKLALAKQLNLSPK

QVEVWFQNRRA

>EgHD-Zip31

MAMTMVPHRESSSGSINKHLTDSGKYVRYTAEQVEALERVYSECPKPSSLRRQQLIRECPILSNIEPKQIKVWFQNRRCR

EKQRKEASRLQTVNRKLTAMNKLLMEENDRLQKQVSQLVCENGYMRQQLHTTSATTTDASCDSVVTTPQHSLRDANNPAG

LLSIAEETLAEFLSKATGTAVDWVQMPGMKPGPDSVGIFAISQSCSGVAARACGLVSLEPTKIVEILKDRTSWFRDCRSL

EVFTMFPAGNGGTIELVYTQIYAPTTLAPARDLWTLRYTTTLENGSLVVCERSLSGSGAGPNPASATQFVRAEILPSGYL

IRPCEGGGSIIHIVDHLNLEAWSVPEVLRPLYESSKVVAQRITIAALRYIRQIAQETSGEVVYGLGRQPAVLRTFSQRLS

RGFNDAVNGFNDGGWSLMNGDGAEDVMIAVTFSKKLNTTSNPANPLSFVGGILCAKASMLLQNVPPAVLVRFLREHRSEW

ADFNVDAYSAASLKASPFGYPGMRPTRFTGSQIIMPLGHTIEHEEMLEVIRLEGHSLAQEDAFVSRDIHLLQICSGIDEN

AVGVCSELIFAPIDEMFPDDAPLLPSGFRIIPLDSKSSDVQDSLTTNRTLDLTSSLEVGPASTNCVGDVAPSHGARSVLT

IAFQFPFDANTQDNVAVMARQYVRSVISSVQRVAMVISPSGLGPSINPKLSQGSPEALTLANWICQSYSLYLGTELLGSD

LLGADSMLKTLWSHQDAILCCSLKSIPVFIFANQAGLDMLETTLVALQDITLDKIFDESVRKELSPEFAKLMQEGSAYLP

SGICMSTMGRHVSYEQAIAWKVLSAEENTVHCLAFSFVNWSFV

>EgHD-Zip32

MELGLSLGDAPFLEKEDLGFHLGLGSSFCGRPKEARDHGCGDVEDKSKRPRDAVALQLHLFPSRSPSLLPQLNDNNGDSS

DGLAEGKWLPSRLLATAAAAKTELVDAGAERLSSPNSGTSSFHQLEFRSGSGGRSKTVDALSLEGDNERACSKGSDEYDE

DNGLARKKLRLSKEQSAFLEESFKEHHTLNPKEKLALARQLNLRPRQVEVWFQNRRARTKLKQTEVDCEHLKRCCERLTE

ENKRLHKELQELRALKSSHHYSFMKHPVPATTLSMCPSCERISTSTAAATNMNKMPAPSSSLTSANPRTALIHQPLPNNT

QAQAAS

>EgHD-Zip33

MKRLSSSDSSDVWISMCSGKEEKVLKKSQGYSIEFQAMLDSLDQEDHSGEEAGLITEKKRRLSANQVKALEKNFEIENKL

EPERKARLAEELSLQPRQVAIWFQNRRARWKTKQLERDFGHLKASYDSLKLDFDSLEQEKESLAAELTELKVKLRRETSE

SSNHCAVKHESPLSESSEDGKPGSCSGSVKEDPNPTPELPPSSAAPPLPLRYGSCSTSPPPPPPPPPPPSSSRGTTAGRG

YYHQVRMEENHPSGFISEESCNFFSVDQPPTLHWYFP

>EgHD-Zip34

MELALTLGDTSKPFSFLDKAPKMAGDKESGNRDLGFCMGLGVSGRSSRGGGDEEEDGDGDKRESPATDRRHHQQHHPPST

LLQLHLLPFSPTNPRNSHHSHKPSTSQFRLPWLTDHLGSEPESSGGAGRGLDVNRFPAAEEAEQSSPNSALSSFQMEFGI

RSGGGAGFRSGSHKRVDMEGDNERDCSRGSDDDDNGLTRKKLRLSKEQSAFLEESFKEHNTLNPKQKLALAKQLNLRPRQ

VEVWFQNRRARTKLKQTEIDCEYLKRCCETLTEENRRLQKELQELRALKTSQPFYMQLPATTLTMCPSCERVATTTAPAS

AASSPAAAAAAPPLAKPKPRTYPFPHAPQLHHLPQAQPQAHQAAS

>EgHD-Zip35

MAFPPPSASSAASFMFQAHHHHHQEDPLPSIIPLNHSNGDVVNNVPFLMKRSMSFSGADHNQRSCGGGDDDDLSDDGSQL

LLGERKKRLSLEQVKALEKSFEIGNKLEPERKIQLARALGLQPRQIAIWFQNRRARWKTKQLERDYEVLKKQFEVLKAEN

DALHAQNEKLHAELLVLKGRDTNEAPSNLKKEIERSYWSNGSENSSDINLDISRTSVMNSPVSSSQFSNKHHFTSSRPS

>EgHD-Zip36

MAFFSPNFMLQSPHDQDHEHPHHQHQHQILSSCTPQDFHGVASLLGKRSMSFTGIDVGDDPNINNGNVNGEEDLSEDDGS

QPGGEKKRRLNMEQVKTLEKNFELGNKLEPERKMQLARALNLQPRQIAIWFQNRRARWKTKQLEKDYDLLKRQFDAVKAD

NETLQAQNQKLQTEILALKNTREPAESINLNKETDQGSCSNRSENSSEIRLDMTRTPPVESPVSGHALPAAGRQLFPASM

RPAASGGSVAQLFQNPSRPDLPMIVKEESSITNMFCGIEDHSGFWPWLEQQHY

>EgHD-Zip37

MSFSGADHNQRSCGGGDDNDLSDDGSQLLLGKKRLSLEQVKALEKSFEIGNKLEPERKIQLARALGLQPRQIAIWFQNWR

ARWKTKQLERDYEVLKKQFEVHAQNEKLHAELLVLKGRDTNEAPSNLKKEIERSYWSNGSENSSNINLDISRTSVMNSPV

SSSQFSNKHHFTSWRPSVMAQQLHGSTR

>EgHD-Zip38

MADSGGSGGSGEEHEHRQDSHPSPNPSSSSRKGKGKSKTYHRHTPHQTQRLEANFRTPDENQRRQLSRELGLDPKQIKFW

FQNKRTQRKTQNERADNTALRAENERIHCENLAIMEALKSVICPACGGPPFGEEERQRNLEKLKMENVQLKEEHAKVSKI

LAKYIGKPISQIDLLMPGPRSSADVSTASVPNQGFSGHNVDFVVAPPNPAPWSQLKVIPETEMADTLEITAYAMDELIRI

VRMKEPLWIKSPLDGRYILHRDTYEQIFSRNYRFRGSGARLETSKESVLVTMEPSRLVNIFLDVRKWADMFHTIVTKASS

IPVLEEGTQLNHRIYLQLIHARMHVLSPLVPAREVYFLRRCQQIELGMWVIVDVSYNYMTGDACPYNTWRLPSGCVIQDM

GNGCSKVTWVEHVEVDDKNQSHKLFRDLVCGGLTYGAERWAITLQRMCERFAYSMLDDAPTHDAVEVGITTSPVIVVPEG

RRNVMKLAHRMVKNFSAGLSMSGKLDFPHLSEVNNSGVRVCVRKSEEPGQPSGTIVSAATSLWLPVAPQAVFNFFRNENT

RAQWDVLCNGNPVQEIARIPSGTHHGNSISIIQTSLHAENMLMLQESCIDPLTCMVVYAPIDSTAMTIALSGEDTSTVQI

LPSGFTISSDGRANPGSKPSTSTSSSKPAGTLLTVAFQILVSSHSGPEQLNVESVATVNTLISATVQKVKAALNCSAPE

>EgHD-Zip39

MKIWKSHQTNSKTRPFFGGVGVNRDILVVDLDDDDHVSSPNSTVSSISGKRSERDPHGENEVEAERTSCSCESIEEDGSG

GDGARKKLRLSREQSLVLEETFKEHNTLNRVEVWFQNRRARTKTKQTEVDCEYLKRCCNNLTQENKRLQKEVQELRALKL

SPQLYMHLNPPTTLTMCPSCQRVSVPSASLSPPSSSSVTASVIGPIGPVHNPFSQSGPSINPWARLQMQQGLNNLHS

>EgHD-Zip40

MGERDDLGLSLSLSFPQGHLHQQQQQQQRQSLQLNLMPSLVPSSASSAQSGFNLQKRSCNDAFPSSSDRNSEARSFLRGI

DVNREPSAGAAADYGEDEAGVSSPNSTASTVSGKRSERDHQSQTNGDDLDNERASSRGGGSDEEDGDMSRKKLRLSKDQS

AVLEESFKEHNTLNPWVRFMQKQKLALAKQLGLRPRQVEVWFQNRRARTKLKQTEVDCEYLKRCCESLTEENRRLQKEVQ

ELRALKLSPQFYMHLSPPTTLTMCPSCERVAAPSPPSAVGRPLAAVPAHPRPVPLINPWAPAAAPLAHAPFDALRSCS
